# Comparative kinomics of human and chimpanzee reveal unique kinship and functional diversity generated by new domain combinations

**DOI:** 10.1186/1471-2164-9-625

**Published:** 2008-12-23

**Authors:** Krishanpal Anamika, Juliette Martin, Narayanaswamy Srinivasan

**Affiliations:** 1 Molecular Biophysics Unit, Indian Institute of Science, Bangalore 560012, India; 2 INRA UR1077, Unité Mathématique Informatique et Génome, F-78350 Jouy-en-Josas, France; 3 Université de Lyon, Lyon, France; Université Lyon 1; IFR 128; CNRS, UMR 5086; IBCP, Institut de Biologie et Chimie des Protéines, 7 passage du Vercors, Lyon, F-69367, France

## Abstract

**Background:**

Phosphorylation by protein kinases is a common event in many cellular processes. Further, many kinases perform specialized roles and are regulated by non-kinase domains tethered to kinase domain. Perturbation in the regulation of kinases leads to malignancy. We have identified and analysed putative protein kinases encoded in the genome of chimpanzee which is a close evolutionary relative of human.

**Result:**

The shared core biology between chimpanzee and human is characterized by many orthologous protein kinases which are involved in conserved pathways. Domain architectures specific to chimp/human kinases have been observed. Chimp kinases with unique domain architectures are characterized by deletion of one or more non-kinase domains in the human kinases. Interestingly, counterparts of some of the multi-domain human kinases in chimp are characterized by identical domain architectures but with kinase-like non-kinase domain. Remarkably, out of 587 chimpanzee kinases no human orthologue with greater than 95% sequence identity could be identified for 160 kinases. Variations in chimpanzee kinases compared to human kinases are brought about also by differences in functions of domains tethered to the catalytic kinase domain. For example, the heterodimer forming PB1 domain related to the fold of ubiquitin/Ras-binding domain is seen uniquely tethered to PKC-like chimpanzee kinase.

**Conclusion:**

Though the chimpanzee and human are evolutionary very close, there are chimpanzee kinases with no close counterpart in the human suggesting differences in their functions. This analysis provides a direction for experimental analysis of human and chimpanzee protein kinases in order to enhance our understanding on their specific biological roles.

## Background

Protein phosphorylation is a core process in many signal transduction pathways which regulates various aspects of cellular processes. Members of the family of protein kinases mediate protein phosphorylation. The family of Ser/Thr and Tyr protein kinases is one of the largest protein families. Members of this family are involved in regulation of a wide variety of cellular processes such as cell growth and differentiation, cell cycle control, apoptosis, transcription, cell motility and cell-cell communication. By covalent tethering of phosphate group from ATP, protein kinases modify the functional activity of the substrates to result in changes in the levels of enzyme activity, cellular location or association with various other proteins in the proximity [1]. The catalytic domain of protein kinases is approximately 250–270 amino acids long and shares a common three-dimensional fold [2]. Despite these common features, protein kinases interact specifically with varieties of substrates and are regulated by a variety of means such as binding of second messengers, phosphorylation within and outside catalytic kinase domain and association with adapter and regulatory subunits. The differing substrate specificities and modes of regulation are contributed mainly by specific sequence variations within the kinase domain as well as by the occurrence of characteristic non-kinase domains tethered to kinase domains in multi-domain kinases [3-5]. Since protein kinases have great impact on various cellular processes, their cellular activity is tightly regulated. Any perturbation in protein kinase activity results in various diseases such as cancer and tumor. As a result protein kinases are popular drug targets in cancer therapeutics.

The family of protein kinases studied in this work comprises of serine/threonine and tyrosine kinases. Various protein kinases involved in signaling pathway are conserved from yeast to human [6]. Explicit analysis of the human kinome made in our group [3] as well as in the groups of Hunter and coworkers [7] and Kostich et al [8] provided a comprehensive picture for the first time on the repertoire of human kinases [9]. The availability of draft version of chimpanzee (*Pan troglodytes*) genome by the Chimpanzee Sequencing and Analysis Consortium [10] has given us opportunity to decipher the role of protein kinases in signaling pathway of our closest evolutionary relative and to compare the protein kinases of chimpanzee with human in order to expand our understanding of human biology. The draft version of the chimpanzee genome revealed that the difference between chimpanzee and human genome is only 1.2% [10,11] and the major part of the genomic divergence could be attributed to insertions and deletions (indels) [12]. Human specific indels contribute towards human specific traits by changing the RNA and protein expression level [13] which might leads to huge difference in the areas such as brain size and behaviour. The divergence in the 3' UTR and 5' UTR of chimpanzee genes contribute towards differences in the gene regulation between human and chimpanzee genomes [14].

The comparison of chimpanzee and human genome can give valuable insight about the drastic differences in the physiology (e.g. reproductive biology), anatomy (e.g. unique vertebral column structure) and pathology (rare occurrence of HIV progression to AIDS, neurodegenerative diseases, epithelial cancer, and resistance to *Plasmodium falciparum *infection in chimpanzee) between the two genomes. The current analysis is, however, confined to recognizing kinases that are common and different between human and chimpanzee and the kinases that are unique for chimpanzee or human. Comparison of chimpanzee and human kinomes will help us to study the major differences and similarities between chimpanzee and human signal transduction pathways involving protein kinases.

Analysis of kinomes of several organisms performed in our group and in other groups [3,6,7,15-21] revealed that kinase complement represents 2–3% of the proteome. In the current study, using the sensitive bioinformatics approaches previously established by us in the studies on various kinomes [3,5,17,20,21] we have identified 587 putative protein kinases (PPKs) in chimpanzee and it corresponds to approximately 1.8% of the genome size. The PPKs are further classified based upon Hanks and Hunter classification scheme [22].

Classification of protein kinases into various Hanks and Hunter subfamilies [22] reveals many kinases that are conserved between chimpanzee and human, which reflects functional constraints of these PPKs in the core of signaling pathway. Apart from the kinase catalytic domain, we present our analysis on other (accessory) domains found tethered to the kinase domain. This information can be retrieved online from the KinG website [23] at . The final list of PPKs identified in the chimpanzee genome has been carefully arrived at after eliminating the redundant sequences (identity>95%) and the sequences lacking key functional residues like Aspartate (D) [acts as base – accepts the proton from the attacking substrate hydroxyl group during phosphotransfer mechanism] in the catalytic loop. Short and truncated sequences, which are less than 200 amino acids long, have not been considered in the present analysis. Hence all the sequences present in the current analysis are likely to be active gene products.

The objective of this study is to identify, classify and annotate the protein kinases in chimpanzee genome and to compare this preliminary kinome set with human which is the closest relative of chimp.

## Results and discussion

Chimpanzee and human putative protein kinases (PPKs) are identified using the approach described in the *Methods *section. Based upon Hanks and Hunter classification scheme, these PPKs are further classified into various protein kinase subfamilies. Accessory domains, transmembrane segments if any, have also been identified. Additional file 1 lists the kinases encoded in chimpanzee genome with details of subfamily association for kinase catalytic domain and domain architecture. Here, we present repertoire of chimpanzee protein kinases, which has been identified and analyzed for the first time, to the best of our knowledge. Further, it has been compared with the human kinome.

**Figure 1:**
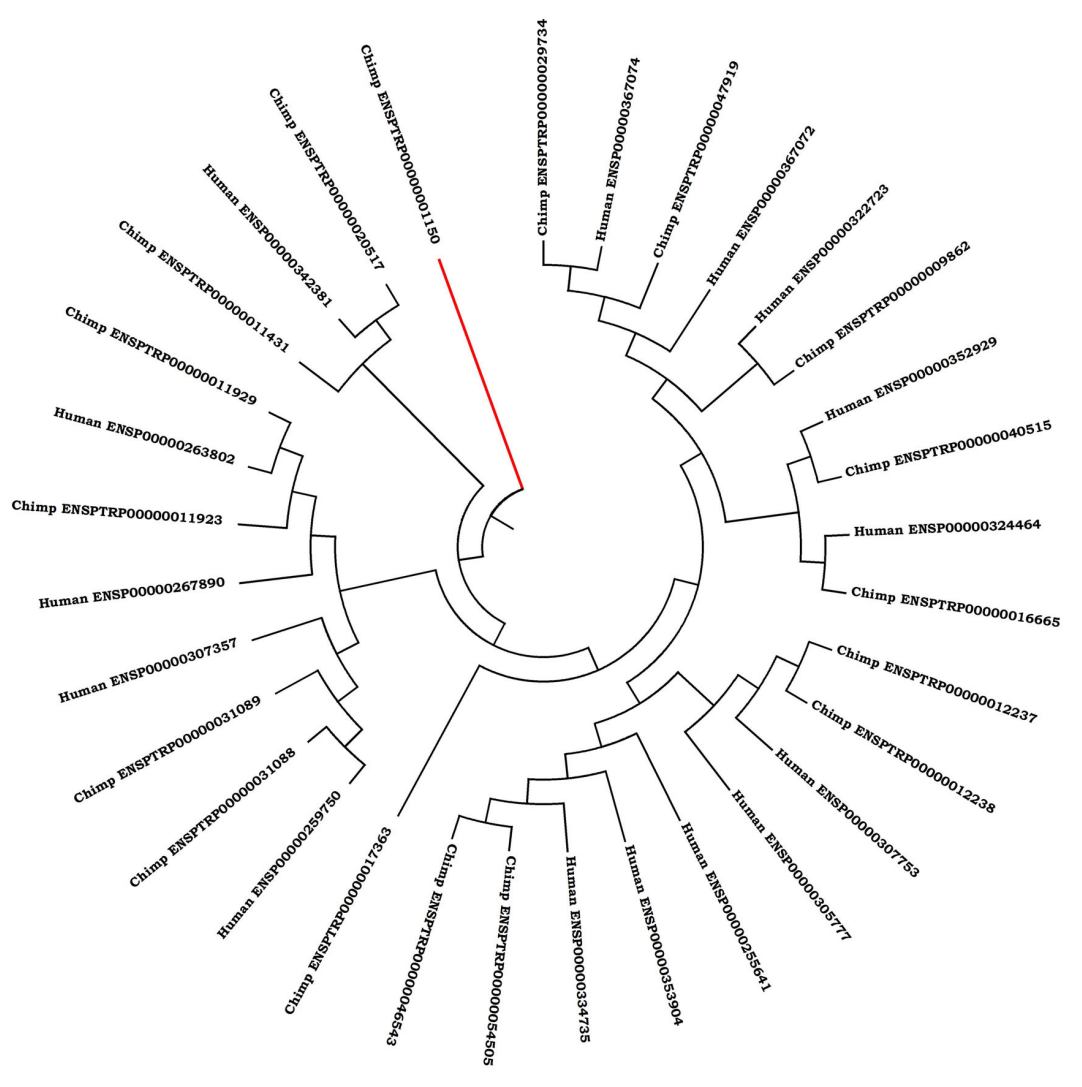
**Dendrogram showing putative casein kinase 1 members from chimpanzee and human**. The diverged member of chimpanzee ENSPTRP00000001150 is shown in red colour. This sequence is closely related to polo kinase in terms of domain combination. However the close human homologue PLK3_001 does not belong to the CK1 family.

We have identified 587 kinases from chimp genome using various sophisticated sequence analysis and classification procedures described in the section on Methods. We have classified these kinases into various subfamilies and compared the distribution with that of human kinases (Table 1). As can be seen in Table 1 almost all the subfamilies of protein kinases are represented in comparable population in human and chimpanzee. This is in contrast to mouse kinome in which there is dramatic expansion of Microtubule affinity-regulating kinase (MARK) gene, loss of a few protein kinase genes as compared to human kinome and lineage specific protein kinases derived from retrotransposition [24]. These observations are consistent with our notion of relatively higher evolutionary divergence between human and mouse as compared to human and chimp. However some of the chimpanzee kinases are radically different from the nearest human kinases (Table 2). For example, a chimpanzee kinase classified as casein kinase 1 (ENSPTRP00000001150) on the basis of significant sequence similarity (31%) of the catalytic domain and excellent e-value (2e-16) with the casein kinase 1 from human. However this chimp kinase has a POLO BOX tethered to the kinase catalytic domain. Thus this chimp kinase represents a hybrid CK1_POLO kinase. Interestingly ENSEMBL reports that ENSPTRP00000001150 has a high similarity with the human kinase ENSP00000361275. However, according to our classification protocol ENSP00000361275 is classified as a POLO kinase on the basis of 52% sequence identity with classical POLO kinases and excellent e-value of e-112. Figure 1 shows the dendrogram of the CK1 sub-family of kinases and it highlights the significant divergence of chimp homologue from its counterparts in other organisms.

**Table 1:** Number of PPKs, in various Hanks and Hunter subfamilies, identified from human and chimpanzee genomes

**Protein Kinase Subfamily**	**Chimpanzee**	**Human**
Agc1	14	15

Agc2	16	16

Agc3	2	3

Agc4	5	6

Agc5	0	0

Agc6	11	11

Agc7	0	0

Agc8	0	0

Agc other	33	36

Camk1	60	66

Camk2	29	36

Camk other	2	2

Cmgc1	20	22

Cmgc2	15	18

Cmgc3	2	2

Cmgc4	2	2

Cmgc5	24	25

CmgC other	12	15

Ck1(csnk)	17	15

Mekk/ste11	10	13

Mek/ste7	8	8

Mlk	4	5

Nima	14	14

Pak	6	10

Pkn	0	0

Plantrk	6	6

Polo	4	5

Ptk1	4	3

Ptk2	6	7

Ptk3	5	3

Ptk4	3	3

Ptk5	3	3

Ptk6	3	3

Ptk7	4	4

Ptk8	13	19

Ptk9	8	5

Ptk10	3	3

Ptk11	13	14

Ptk12	3	3

Ptk13	3	3

Ptk14	7	8

Ptk15	8	12

Ptk16	3	3

Ptk17	3	3

Ptk18	8	8

Ptk19	9	9

Ptk20	3	3

Ptk21	3	3

Ptk22	0	0

Ptk23	1	1

Raf	14	13

Tgfb	16	16

Translationk	6	6

Wee1	2	2

Unclassified Kinase	117	83

**Table 2:** Chimp protein kinases with significant difference compared to their closest human homologues.

**Chimp protein kinase and kinase subfamily assignment**	**Nearest human protein kinase and kinase subfamily assignment**	**Sequence identity between Chimp sequence & human sequence**	**Remarks**
ENSPTRP00000038601(CAMK1)	ENSP00000359161 (CAMK1)	86.07%	The N-terminal part of the Chimp sequence containing glycine motif in the kinase domain is missing as compared to the human kinase domain

ENSPTRP00000044216 (AGC_other)	ENSP00000302750 (AGC_other)	75.57%	The human sequence has DMPK_coil domain which is lacking in its closest Chimp sequence. However, domain organization given below is present in other Chimp kinases. **Human sequence: **Pkinase, Pkinase_C, DMPK_coil, C1, PH, CNH **Chimp sequence: **Pkinase, Pkinase_C, C1, PH, CNH

ENSPTRP00000000076 (AGC2)	ENSP00000367830 (AGC2)	87.5%	Human sequence has C1 domain which is lacking in the Chimp sequence **Human sequence: **PB1, C1, Pkinase, Pkinase_C **Chimp sequence: **PB1, Pkinase, Pkinase_C

ENSPTRP00000037507 (RAF)	ENSP00000290277 (RAF)	67.47%	Human sequence has C1 domain is which is lacking in the Chimp sequence **Human sequence: **RBD, C1, Pkinase **Chimp sequence: **RBD, Pkinase

ENSPTRP00000011569 (CAMK1)	ENSP00000270202 (AGC3)	99.06%	The Chimp kinase which belongs to CAMK1 is having PH domain N-terminal to the kinase domain which is very unusual as PH domain is not notmally seen seen with CAMK1. Moreover, the domain organization of these two protein kinases are different. Human protein kinase has Pkinase_C domain in the C-terminal which is lacking in the Chimp protein kinase. Human sequence: PH, Pkinase, Pkinase_C Chimp sequence: PH, Pkinase

ENSPTRP00000022133 (Unclassified)	ENSP00000311684 (CAMK1)	95.2%	The close Chimp homologue of this human sequence is PKLNK **Human sequence: **I-set X 6, fn3, I-set X 2, Pkinase, I-set, fn3, Pkinase **Chimp sequence: **I-set X 6, fn3, I-set X 2, Pkinase, I-set, fn3, PKLNK

ENSPTRP00000027141	ENSP00000371341 (PTK8)	59%	The close Chimp homologue of this human sequence is PKLNK **Human sequence: **Pkinase, SH3, GTPase binding domain **Chimp sequence: **PKLNK, SH3, GTPase binding domain

ENSPTRP00000027139	ENSP00000323216 (PTK8)	70.6%	The close Chimp homologue of this human sequence is PKLNK **Human sequence: **Pkinase, SH3, GTPase binding domain **Chimp sequence: **Pkinase, SH3, GTPase binding domain

ENSPTRP00000027136	ENSP00000329425 (PTK8)	57.1%	The close Chimp homologue of this human sequence is PKLNK **Human sequence: **Pkinase, SH3, GTPase binding domain, UBA **Chimp sequence: **PKLNK, SH3, GTPase binding domain, UBA

ENSPTRP00000001150 (CK1)	ENSP00000361275 (Polo)	65.5%	The Chimp kinase is closely related to CK1 but it has POLO box domain C-terminal to the kinase domain

ENSPTRP00000020259	ENSP00000306717	94.6%	The Human kinase has Ldl receptor A domain in the N-terminus which is lacking in the Chimp homologue **Human sequence: **Ldl receptor A, MAM, Pkinase **Chimp sequence: **MAM, Pkinase

ENSPTRP00000019171	ENSP00000291270	94.2%	The Chimp kinase sequence has putative transmembrane region in the C-terminus which is absent in the Human kinase. **Human sequence: **Pkinase, Pkinase_C, DMPK_coil **Chimp sequence: **Pkinase, Pkinase_C, DMPK_coil, TM

Abbreviations followed in the table: Pkinase, Protein kinase; PH, Pleckstrin homology domain; CNH, Citron homology domain; DMPK_coil, Dystrophy myotonic protein kinase coil domain; C1, Phorbol esters/Diacylglycerol binding domain; PKLNK, Protein kinase-like non-kinase; fn3, Fibronectin type III domain; Ig, Immunoglobulin domain; I-set, Immunoglobulin I-set domain;RhoGeF, Guanine nucleotide exchange factor for Rho/Rac/Cdc42-like GTPase domain; PB1, Phox and Bem1p domain; TM, Transmembrane; SH2, Src homology 2; RBD, Raf-like Ras-binding domain; SH3, Src homology 3; UBA, Ubiquitin associated domain; Ldl receptor A, Low-density lipoprotein receptor domain class A.

**Figure 2:**
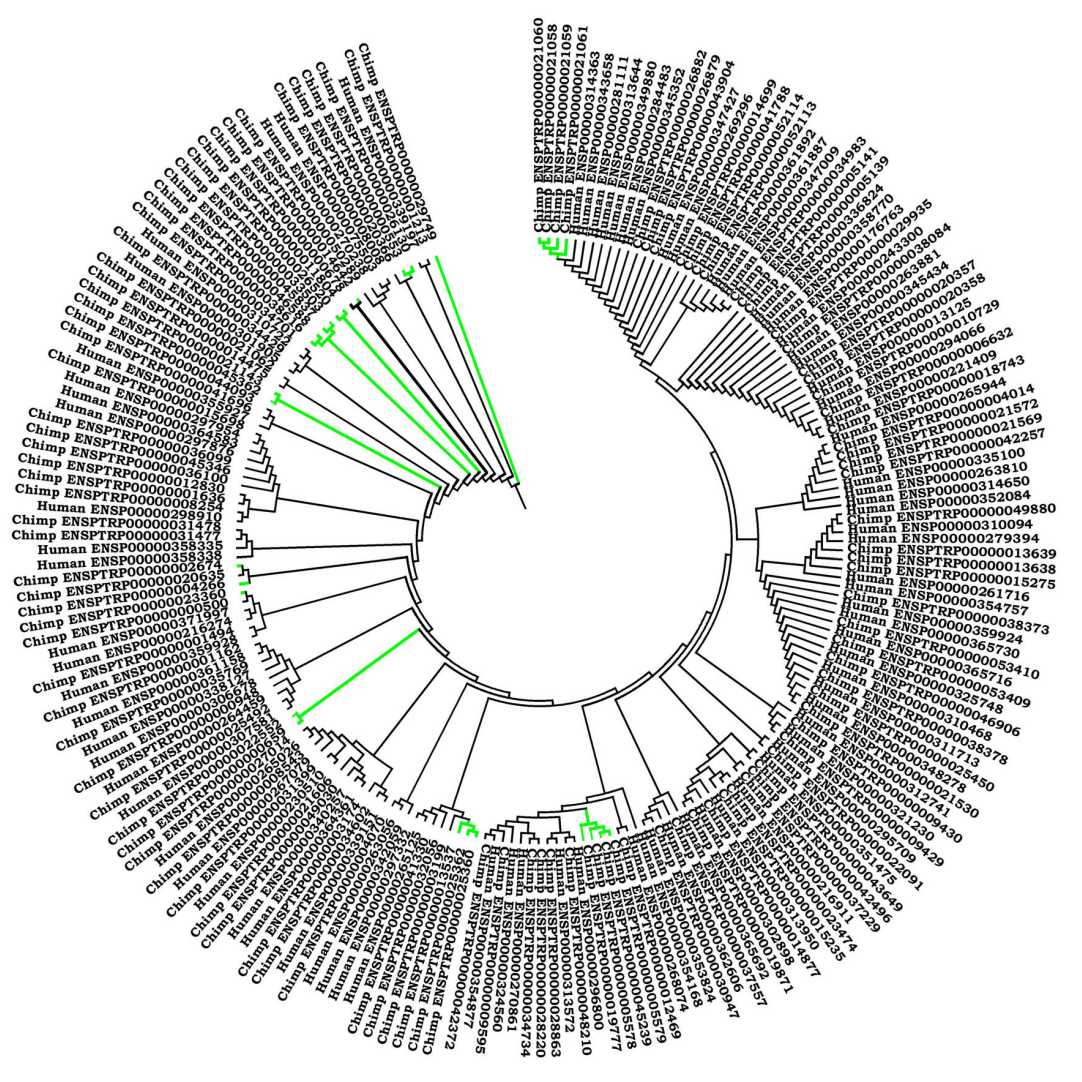
**The dendrogram showing putative unclassified protein kinase members from chimpanzee and human**. Chimpanzee specific unclassified protein kinases with no closely-related human homologues are represented by green colour. Clusters which are represented in black colour are showing protein kinases from chimp and human which are closely related e.g. chimp proteins kinase ENSPTRP00000034429 represented in this figure is closely related to the human protein PBK-001 (ENSP00000301905).

Many chimp kinases are unclassified as the extent of similarity of the catalytic regions to any of the previously known kinase sub-families is very low. However many of these unclassified chimp kinases have corresponding closely-related human kinases. A dendrogram of unclassified chimp and human kinases (Figure 2) demonstrates close relationship between unclassified kinases between chimp and human. However a few chimp kinases without a closely-related human counterpart can also be noticed.

Though many kinases between chimp and human are highly similar the cases of significant difference emerge mainly due to difference in domain architecture of multi-domain kinases of chimp and human. Following sections discuss the differences between some of the chimp and human kinases arising out of difference in domain tethering features. Additional file 2 shows amino acid sequence alignments for some of the pairs of chimp and nearest human kinases that are characterized by pronounced differences in terms of domain architecture.

### Domain composition of chimpanzee kinome

Many of the protein kinases in higher eukaryote are multi-domain proteins; the kinase domain is tethered to various non-kinase domains. We examined the global domain composition of 587 putative kinases encoded in the chimpanzee genome. Globally, 82 different domains, other than kinase domains and Pkinase_C (the protein kinase C terminal) domain, are seen in the 587 chimpanzee kinases. A list of the most frequently found accessory domains is provided in Table 3. The most frequent domain tethered to protein kinase domain is the immunoglobulin I-set domain, a member of the immunoglobulin superfamily. The I-set domain is found in a variety of protein families. It is seen 96 times, but only in 34 proteins and it means that it often occurs as repeats. The second most frequent domain, Fibronectin type III domain (fn3), belongs to the Ig-like fold superfamily. It also occurs in repeats: it is seen 67 times in 27 PPKs. It is involved in cell surface binding. Ankyrin repeat is the most common protein-protein interaction motifs in nature. It is seen 51 times in six chimpanzee PPKs. The immunoglobulin domain (Ig), a member of the immunoglobulin superfamily, which is present 46 times in 29 PPKs is also found tethered to protein kinase domain as repeats. SH2 domain acts as a regulatory module, by interacting with phosphotyrosine-containing peptides; it is seen 32 times in 29 PPKs. C1_1 is the phorbol esters/diacylglycerol binding domain, found tethered to diacylglycerol dependent protein kinases C (PKC). It is found 30 times in 22 PPKs. In the case of human protein kinases also, the above mentioned non-kinase domains are most frequently occurring as accessory domains to the protein kinase domain.

**Table 3:** Most frequent accessory domains found in the chimpanzee and human PPKs.

	Chimpanzee		Human	
Domain, *short domain id*, PFAM id	Freq^a^	Nb_prot_^b^	Freq	Nb_prot_

Immunoglobulin I-set, *I-set*, PF07679	96	34	106	37

Fibronectin type 3, *Fn3*, PF00041	67	27	69	28

Ankytin repeat, *Ank*, PF00023	51	6	50	6

Immunoglobulin, *Ig*, PF00047	46	29	47	30

Src homology 2, *SH2*, PF00017	32	29	37	34

Phorbol esters/diacylglycerol binding domain, *C1_1*, PF00130	30	22	36	26

Only domains seen more than 30 times are listed. ^a^: domain frequency in the given kinome, ^b^: number of proteins containing this domain.

### Domain architecture analysis – chimpanzee-specific and human-specific

In the recent Pfam version 22, the protein kinase domain (PF00069) is seen in 771 different architectures, and the protein tyrosine kinase domain (PF07714) is seen in 333 different architectures. In the repertoire of chimpanzee protein kinases, out of 587 PPKs, 286 PPKs have accessory domains tethered to the kinase domain. The rest of the protein kinases do not have any other domain tethered to the kinase domain.

**Figure 3:**
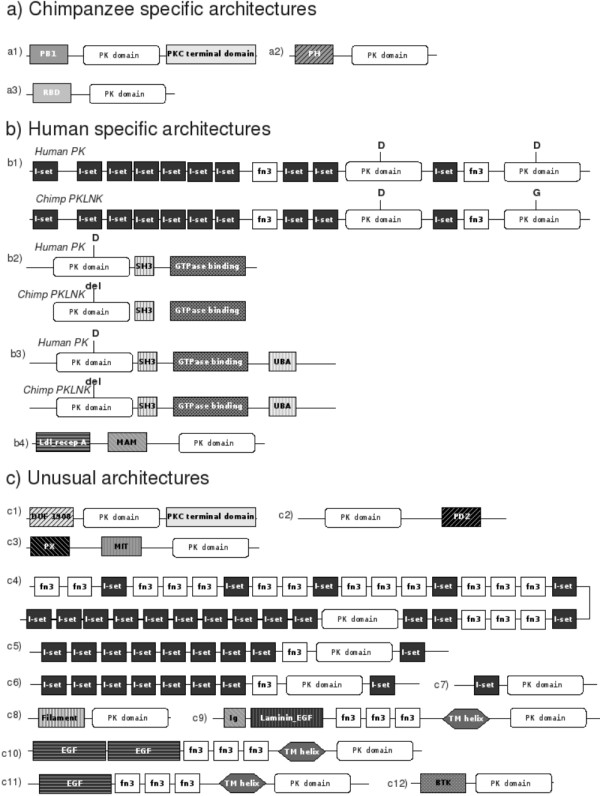
**Organism specific and unusual domain architectures in chimpanzee and human kinomes**. Abbreviation followed in the figure: PK, Protein kinase; PH, Pleckstrin homology; RBD, Ras binding domain; I-set, Immunoglobulin I-set; fn3, Fibronectin 3; SH3, Src homology 3; UBA, Ubiquitin associated domain; DUF 1908, Domain of unknown function 1908; PX, Phosphoinositides binding domain; MIT, Microtubule interacting and transport; Ldl recep A, Low density lipoprotein receptor domain class A; MAM, MAM domain; Ig, Immunoglobulin; Laminin_EGF, Laminin epidermal growth factor like; BTK, Bruton's tyrosine kinase; EGF, Epidermal growth factor; TM, Transmembrane; D, Aspartate; G, Glycine; Del, Deletion in the alignment.

The comparison of chimpanzee and human kinomes revealed some organism-specific domain architectures (see *Methods *section). We have also identified unusual domain architectures. These domain architectures are common only to human and chimpanzee with no or poor representation in other organisms with known genomic data at the present time. We describe here the chimpanzee and human specific architectures and the unusual domain architectures common to chimpanzee and human (Figure 3).

### Chimpanzee specific multi-domain architectures of kinases

We have identified three PPKs with chimpanzee specific domain architectures, depicted in figure 3a. These PPKs belong to the AGC group, the CAMK group and the Raf kinase.

A protein (ENSPTRP00000000076), classified under PKC subfamily, is composed of a PB1 domain followed by the protein kinase domain which is followed by a protein kinase C terminal domain (Figure 3a1). The PB1 domain is present in many eukaryotic cytoplasmic signalling proteins and is responsible, although not systematically, in the formation of PB1 dimers [25]. It thus serves as a molecular recognition module. This architecture is known so far only in an atypical PKC of *Phallusia mammilata*, a sea squirt. Our analysis identified two chimpanzee PKCs and a human PKC with a similar architecture, in which a phorbol esters/diacylglycerol binding domain is inserted between the PB1 and the protein kinase domain. The presence of the phorbol esters/diacylglycerol binding domain in combination with the protein kinase and a PKC terminal domain indicates that it is probably responsible for the recruitment of diacylglycerol, which in turns might be involved in activation of the kinase. The deletion of this domain in chimpanzee PKC (ENSPTRP00000000076) implies that the recruitment of diacylglycerol might be achieved by an external interacting module.

In a chimpanzee PPK classified under the camk1 subfamily (ENSPTRP00000011569), the pleckstrin homology (PH) domain is followed by the protein kinase domain (Figure 3a2). The PH domain is seen in a variety of intracellular signaling proteins. It can perform various functions including binding of phosphorylated serine/threonine residues [26], and interacting with calcium ion and calmodulin. This protein, annotated as novel peptide, is closely related to a human PKC (ENSP00000270202) which has an inserted PKC domain at the C-terminal and a longer protein kinase domain. We thus identified a chimpanzee camk1 that is closely related to, but clearly distinct from the nearest human counterpart.

A chimpanzee PPK belonging to the raf subfamily (ENSPTRP00000037507), a novel peptide displays a domain architecture with a RBD (Ras Binding Domain) followed by the catalytic kinase domain (Figure 3a3). This particular protein departs from the typical architecture because it lacks a C1_1 domain (Phorbol esters/diacylglycerol binding domain).

### Chimp counterparts of a few human kinases are kinase-like non kinases

In the human kinome, we have identified a few multi-domain protein kinases having high sequence identity (more than 57%) with chimp homologues over the entire length. These human kinases and the chimp counterparts share almost the same domain architecture. Most interestingly these chimp homologues are unlikely to function as a protein kinase (PKLNK) as they all lack the catalytic base residue (Asp) (Figure 3b) compared to the nearest human kinases.

We identified a human camk1 (ENSP00000311684) with an architecture in which 6 Immunoglobulin domains (I-set) are followed by a fibronectin 3 domain (fn3), followed by two I-set domains, the protein kinase domain, an I-set domain, a fn3 domain, and a second kinase catalytic domain. The close chimp homologue annotated as a known peptide (ENSPTRP00000022133) has PKLNK domain instead of the C-terminal protein kinase domain in the human homologue. This chimp homologue lacks the catalytic aspartate in the catalytic loop (Figure 3b1). I-set and fn3 domains are involved in various biological functions such as cell-cell recognition and immune system, and are found in many protein families, including receptor tyrosine kinases.

In the two human PPKs belonging to ptk8 subfamily (ENSP00000371341 and ENSP00000323216) the protein kinase domain is followed by a Src homology 3 (SH3) domain and a GTPase binding domain. The SH3 domain is seen in signaling proteins related to cytoskeletal organisation [27]. Both these sequences are annotated as 'known-ccds peptide' and are present in Refseq and Genbank databanks as non-receptor tyrosine kinases. These two human receptor protein kinases are very similar to chimpanzee PKLNKs ENSPTRP00000027141 and ENSPTRP00000027139 respectively (Figure 3b2) in terms of identity between the protein kinase and PKLNK domain and domain architecture.

A related and unique architecture, with an additional C-terminal ubiquitin associated domain, is seen in human PPK (ENSP00000329425) which belongs to ptk8 subfamily. The ubiquitin associated domain is seen in many signaling proteins [28]. ENSP00000329425 is also annotated as a known-cdds peptide and is highly similar to a chimpanzee PKLNK, ENSPTRP00000027136 (Figure 3b3). The fact that these human PPKs with unique architectures are related to non functional chimpanzee kinases is intriguing since they indicate a loss of kinase function in chimpanzee and gain of kinase function in human during the course of primate evolution. These PKLNKs in chimpanzee might be involved in various cellular processes through protein-protein interaction.

A human putative tyrosine kinase, ENSP00000306717 (belonging to ptk17 subfamily), has an unusual architecture which is human specific, where the protein kinase domain is following an Ldl receptor A domain (Low density lipoprotein receptor domain class A) and a MAM domain (Figure 3b4). The Ldl receptor A domain is a short domain that is able to bind Ldl and calcium [29] and the MAM domain is commonly found in receptors and is thought to have an adhesive function. These domains could thus be implicated in the binding of the ligand to the extracellular part of the protein. No close chimp homologue to this human kinase could be identified.

### Unusual domain architectures: Domain architectures conserved only between chimpanzee and human kinomes

We have identified domain architectures in human and chimpanzee kinomes that are poorly or not represented in kinomes of other organisms. These architectures, represented in Figure 3c, fall into the AGC, CK1 and the PTK groups.

#### AGC group

Two AGC PPKs, ENSPTRP00000001185 from chimpanzee and ENSP00000361078 from human, both annotated as 'novel peptide', display an architecture in which a domain of unknown function (DUF 1908) is followed by the protein kinase domain and the PKC terminal domain. DUF 1908 is present in 29 structural eukaryotic proteins in Pfam. Additionally, 14 sequences in Pfam display a similar architecture with an additional C-terminal PDZ domain. Our current survey identified 3 chimpanzee PPKs and 3 human PPKs with this architecture. The PDZ domain that is present in these 6 sequences, but absent from chimpanzee (ENSPTRP00000001185) and human (ENSP00000361078), is 80 to 90 amino-acid long domain and found tethered to diverse signaling proteins. It is involved in protein-protein interaction, and has the capability to bind the C-terminal part of its partner [30]. This suggests that PDZ domains could be involved in ligand recruitment. The lack of PDZ domains in one chimpanzee kinase and its relative in human implies that a compensatory external binding module would be needed to perform the function.

We identified, in human and chimpanzee kinomes, domain architecture of catalytic protein kinase domain which is closely related to AGC group followed by PDZ domain. Such a domain combination is not found in other kinomes. The human PPK (ENSP00000261569), which is the counterpart of ENSPTRP00000029005 of the chimpanzee kinome, is the microtubule-associated serine/threonine-protein kinase 4 (Swiss prot entry: O15021) which is shown to be highly expressed in the brain [31].

A combination of PX, MIT and the protein kinase domain is seen in chimpanzee PPK (ENSPTRP00000003319), a novel peptide, and one human PPK (ENSP00000355927), labeled as known-cdds. PX domain is a phosphoinositides binding domain and MIT is the microtubule interacting and transport domain. Human PPK (ENSP00000355927) is highly similar to the Ribosomal protein S6 kinase delta-1 (swiss-prot entry Q96S38) which is known to be expressed in brain [32,33].

#### CAMK group

CAMK kinases have fibronectin 3 (fn3) and immunoglobulin I-set domains in a variety of combinations. We identified 4 types of architectures in PPKs which are closely related to camk1 subfamily, and are not present in kinomes of other organisms:

- *'fn3 x 2, I-set, fn3 x 3, I-set, fn3 x 2, I-set, fn3 x 3, I-set, fn3 x 3, I-set x 2, fn3 x 2, I-set x 2, fn3, Pkinase, I-set x 10' *: this architecture is seen in a chimpanzee PPK (novel peptide ENSPTRP00000021693) and a human PPK (novel peptide ENSP00000343764).

- *'I-set x 8, fn3, Pkinase, I-set'*: in two chimpanzee PPKs (novel peptides ENSPTRP00000046634 and ENSPTRP00000045642) and two human PKKs (ENSP00000353452, a novel peptide and ENSP00000354004, a known peptide). The latter corresponds to myosin light chain kinase (swissprot entry Q15746).

- *'I-set x 7, fn3, Pkinase, I-set' *: in a chimpanzee PPK (novel peptide ENSPTRP00000026393) and in a human PPK (known peptide ENSP00000320622). The latter sequence corresponds to the entry NP_444254 in the RefSeq database of NCBI and it is the isoform 2 of Myosin light chain kinase.

- *'I-set, Pkinase'*: in a chimpanzee PPK (novel peptide ENSPTRP00000028713) and a human PPK (known peptide ENSP00000339291).

#### CK1 group

A Filament domain is seen associated to the protein kinase domain of chimpanzee PPK (ENSPTRP00000011929) and human PPK (ENSP00000263802). Filament domains are components of the cytoskeleton and the nuclear envelope [34]. To our knowledge, this kind of domain composition having protein kinase domain and Filament domain together is identified here for the first time.

#### PTK group

Four domain architectures seen in PTK group are poorly or not represented in kinomes of other organisms. A chimpanzee PPK (the novel peptide ENSPTRP00000001085) and a human PPK (the known-ccds ENSP00000361554) have an architecture composed of an immunoglobulin domain, followed by a Laminin EGF-like domain, an EGF domain, three fibronectin 3 domains and the catalytic protein kinase domain. A transmembrane helix is predicted with high confidence between the fibrnonectin 3 domain and the kinase domain. Laminin EGF-like domain is found in laminin which is the constituent of basement membranes. The functional role of EGF (epidermal growth factor) domain is unclear; it is seen in a variety of membrane and secreted proteins [35]. This architecture is not seen in kinomes of other organisms but a related architecture lacking the fn3 domains is reported for 6 sequences. It is related to a tyrosine-protein kinase receptor Tie-1 (corresponds to swissprot entry P35590), and is specifically known to express in developing vascular endothelial cells.

A combination of two EGF hand domains with three fibronectin 3 domains, followed by a transmembrane domain and the protein kinase domain is seen in a chimpanzee PPK (ENSPTRP00000035632, a novel peptide), and in a human PPK (ENSP00000343716, labeled as known-ccds) and corresponding to the Tyrosine-protein kinase receptor TIE-2 (swissprot entry Q02763). This architecture is present in a sequence of mimivirus. A related architecture with only one EGF domain is seen in chimpanzee PPK (ENSPTRP00000035635), and in a human PPK (ENSP00000369375, embl entry CAI16055) which is the TEK tyrosine kinase. Such architecture is not yet known to occur in kinomes of other organisms.

ENSPTRP00000040876, a chimpanzee PPK annotated as a novel peptide in the genome dataset and the human PPK ENSP00000350224 which is also labeled a novel peptide, are composed of a Bruton's tyrosine kinase (BTK) domain and a protein kinase domain. This architecture has not known to occur in other kinomes. BTK domain is a Zinc-binding motif domain [36], usually found associated to other domains like, pleckstrin homology and Src homology domains.

### Orthologous protein kinase pairs of chimpanzee and human

In the current analysis, we have compared 587 PPKs in the chimpanzee genome with human kinases and identified orthologous kinases. To compare genes across these two organisms and to identify the orthologues, we have considered two features: 1) The full length of the two gene products have been compared with the condition that the difference in number of residues of orthologues should not be more than 50. 2) The classification of kinase catalytic domains of orthologues should correspond to same subfamilies of kinases under Hanks and Hunter classification scheme. There are 470 orthologous pairs which we have identified in the current analysis. This information can be found in Additional file 3. A comprehensive comparison of the predicted protein kinases shared by the chimpanzee and human indicates that nearly 81% of the chimpanzee protein kinases have putative orthologs in human genome. Interestingly 427 out of 470 orthologous pairs are characterized by a high sequence identity of at least 95%. For 117 chimpanzee kinases we did not detect human orthologues based on the criteria we have used. Many of these kinases have substantially different lengths and are characterized by differences in domain tethering preferences discussed above.

### Comparison of human and chimp transcriptome

The comparison of humans and chimpanzees with respect to differences in expression levels and protein-coding sequences for genes active in brain, heart, liver, kidney, and testis is available [37]. This data on expression levels was extensively studied by Khaitovich *et al *[38] who have analyzed the difference in the expression levels of various human and chimp proteins. From these papers we have obtained the information pertaining to the difference in the expression level of protein kinase genes between human and chimp in five different tissue types and this data is presented in Additional file 4.

The Additional file 4 highlights the 100 human protein kinase genes which have different expression level as compared to the corresponding chimp protein. As can be seen from this Additional file for many kinases difference in the expression levels in various organs are rarely above 1. So, as concluded in the original analysis [37,38] the expression levels of most chimp and human proteins at various organs are highly comparable. However the most pronounced difference corresponds to expression of a CaMK (ENSG00000071575) in brain (difference of more than 7). But difference in expression of the same kinase in other organs is not as pronounced as in brain. Considering kinases with differences in domain architecture, a human protein kinase gene (ENSG00000104936) which encodes for agc_other subfamily is lacking putative transmembrane domain as compared to its close chimp homologue (Table 2) and the difference of the gene expression level as compared to chimp is more in brain as compared to liver, kidney, heart and testis suggesting variation of gene expression level of this gene in the brain tissues of the two mammals.

## Conclusion

In this report we have made an effort to understand the repertoire of protein kinases in the chimpanzee which has close kinship with human. The comprehensive list and classification of protein kinases in chimpanzee genome presented in this report is a valuable resource for future signaling research for these closely related organisms.

Identification of various PK subfamilies indicates that the core signaling pathway involving PK is well conserved between chimpanzee and human. This implies conserved steps in the signaling pathways in chimpanzee and human involving protein kinase as central player. However, substantial number of chimpanzee kinases deviates markedly in terms of the domain combination/architecture resulting in unique varieties of functional domain combinations. Considerations of functions of modules which are tethered to the protein kinase domain enable us to predict the functional role of PPKs in the chimpanzee genome. Results obtained from this study adds new facets to the contribution of protein kinases in signal transduction of chimpanzee and comparative kinome gives us insights about evolution of protein kinases in two closely related species. Given the importance of protein kinases in signaling, experimental analysis would be fruitful to dissect the signaling system of chimpanzee and will provide valuable insight into the similarities and differences between chimpanzee and human signaling pathways.

## Methods

In this paper, we have identified and classified chimpanzee protein kinases on the basis of the amino acid sequences of putative proteins encoded in the chimpanzee genome. We have compared them with updated list of human protein kinases.

### Data

The chimpanzee and human proteome data are retrieved from the ENSEMBL database [39] release 46 (August 2007). The chimpanzee proteome data is based on the 2.1 genome assembly released by the Chimpanzee Sequencing Consortium in March 2006 [10]. The genome coverage is more than 94% for euchromatic genome . Chimpanzee proteome is composed of 33,167 protein sequences, generated from an automatic annotation system based on biological evidences [40]. The human genome was used as a guide for annotation, by projecting the human gene models onto the chimpanzee genome. The number of experimentally characterized proteins in chimpanzee is very low. In the present data set, 3,373 protein sequences (10% of the data) are labeled as "known peptides", which means that these proteins were mapped to entries in Swiss-Prot, RefSeq or SPTrEMBL during the annotation process. The remaining 29,794 (90% of the data), that could not be mapped to known proteins, are annotated as "novel peptides".

The human proteome data is based on the NCBI 36 assembly of the human genome, released in November 2005. It is composed of 43,570 protein sequences: 3,614 "novel peptides", 18,642 "known peptides" and 21,314 "known-ccds peptides". The "known-ccds" annotation means that these sequences are part of a core set consistently annotated, high quality data in the frame of the Consensus Coding Sequence (CCDS) project.

### Protein kinase detection and analysis

**Figure 4:**
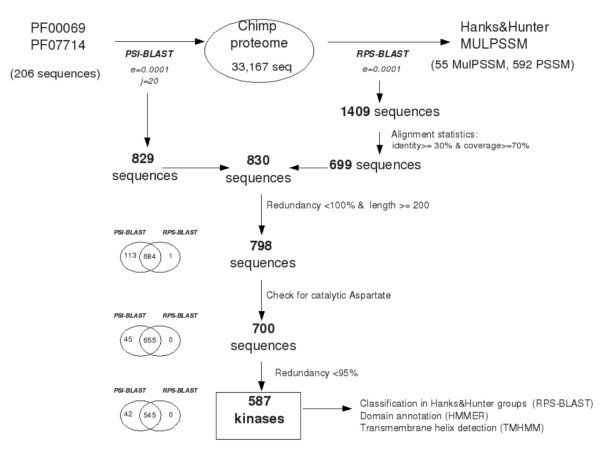
**Procedure involved in the detection of putative protein kinases (PPKs) in the chimpanzee genome**. The number of selected proteins at each step is indicated in the diagram.

Protein kinases are identified using a combination of profile-based search methods such as PSI-BLAST [41] and RPS-BLAST [42] using multiple profiles (MulPSSM) [43,44]. MulPSSM approach has been previously benchmarked and has been used in our previous kinome analysis for several other genomes [3,5,17,20,21]. Summary of the main steps involved in the current analysis is presented in Figure 4.

On the one hand, the proteome of interest is searched for the protein sequences of Pfam families PF00069 (Protein kinase domain, 54 sequences) and PF007714 (Protein tyrosine kinase, 152 sequences). On the other hand, we use multiple PSSMs created for the 55 protein kinase classes [45] represented in Hanks and Hunter classification scheme [22] as query to search against the proteome using RPS-BLAST. For associating a kinase into its Hanks and Hunter subfamily we consider only those hits with sequence identity greater than 30% with bonafide members of the subfamily and the profile alignment coverage greater than 70%.

The results of PSI-BLAST and RPS-BLAST searches have been merged into a single list of putative protein kinases which are further refined using several criteria. Sequences shorter than 200 amino-acids are excluded, as it is well known that the usual length of catalytic region is approximately 250 to 300 amino-acids [22]. Redundancy level is reduced to 100% using cd-hit program [46]. The presence of the catalytic aspartate, essential for the catalytic activity [22], is checked to distinguish between Putative Protein Kinases (PPKs) and Protein Kinase-Like Non-Kinases (PKLNK). Catalytic aspartate has been detected from the multiple sequence alignment of PPK kinase domain generated by clustalW [47] and by manually checking the RPS-BLAST and PSI-BLAST outputs. Suspicious cases were submitted for fold prediction using the PHYRE [48]. After reducing the redundancy level to 95%, we obtain a final set of 587 chimpanzee PPKs. These PPKs are classified according to Hanks and Hunter classification scheme [22]. The population and distribution of chimpanzee protein kinases into various subfamilies has been described in Additional file 5. Further, non kinase domains were analyzed using HMMER [49,50]. Transmembrane regions were detected using TMHMM program [51].

### Identification of organism specific kinase architectures and unusual domain architectures

Protein kinase domain is commonly found tethered to accessory domains; the ordered list of domains along a sequence defines its domain architecture. Here, we compare domain architectures seen in human and chimpanzee kinomes and extract organism-specific and unusual domain architectures. A domain architecture is said to be *organism-specific *if it is found to belong to a particular kinase sub-family in an organism but not in the same kinase family of the other organisms. We also define *unusual *domain architecture as domain architectures identified only in human and chimpanzee kinases, but not present or poorly represented in kinomes of other organisms.

Domains are detected using HMMER and Pfam domain library, and families are defined based upon the Hanks and Hunter classification scheme. Unique and unusual architectures are further checked to ensure that the uniqueness (or unusualness) is not the result of family misclassification or domain mis-assignment. Only significant cases are reported here.

### Kinase regulatory module detection

We searched for the regulatory domains of various PK families. The detection is achieved by RPS-BLAST using multiple profiles of regulatory domains. Sequences of PKA regulatory domains, calmodulins and cyclins were retrieved from the well-annotated sequence data base swiss-prot [52] using the hierarchical classification of Expasy  at the family level. Multiple profiles were generated for each regulatory protein as described in [43] with 44, 89 and 296 sequences of PKA regulatory domains, calmodulins and cyclins respectively. The resulting multiple PSSMs were used to detect the putative protein kinase's regulatory elements.

### Orthology detection

In order to detect orthologous protein kinase pairs between chimpanzee and human, we compared the predicted protein kinases by carrying out all against all BLAST. Firstly, chimpanzee PPK sequences have been used as query against the database of predicted human kinome and secondly predicted human PPK sequences have been used as query against the database of predicted chimpanzee kinome. If the two given sequences are almost of equal length (length difference should not be more than 50 amino acids) and belonging to the same subfamily of protein kinase, then we have considered the two genes as orthologues. Close orthologues have been identified by using a further condition of sequence identity should be greater than 95%.

### Phylogenetic analysis

For the phylogenetic analysis MEGA software [53] has been used to generate dendrogram showing the various subfamilies of predicted protein kinases. Multiple sequence alignment of the kinase domains (generated using clustalW) has been used as input. Maximum parsimony method has been used to generate the dendrogram.

## Authors' contributions

All authors contributed equally to the research. All authors read and approved the final manuscript.

## Supplementary Material

Additional file 1 **Chimpanzee kinases with their subfamily classification, number of residues in the gene product and domains present.** Click here for file

Additional file 2 **Sequence alignment between some of the closest chimp and human kinase pairs with difference in domain architecture.** Click here for file

Additional file 3 **List of chimpanzee protein kinases and their putative orthologues in human.** The Hanks and Hunter protein kinase subfamily to which chimp kinases belong is also mentioned. Click here for file

Additional file 4 **The table representing different gene expression levels for human and chimp protein kinases in five different tissue types.** Click here for file

Additional file 5 **Population and distribution of chimpanzee protein kinases into various subfamilies.** Click here for file
