# Supplementary material for: Comparative kinomics of human and chimpanzee reveal unique kinship and functional diversity generated by new domain combinations
Source: BMC Genomics. 2008 Dec 23;9:625. doi: 10.1186/1471-2164-9-625 (PMC2651890; doi:10.1186/1471-2164-9-625)
Supplement: Additional file 1 — Chimpanzee kinases with their subfamily classification, number of residues in the gene product and domains present. [file 1471-2164-9-625-S1.rtf]

Additional file 1: Chimpanzee kinases with their subfamily classification, number of residues in the gene product and domains present

Protein ID	Length	Protein kinase subfamily	Domain name and boundary	E-value	
ENSPTRP00000014648 	527	p78(camk2)	 Pkinase, 155 431 2.4e-76*	
ENSPTRP00000014651 	515	YCL24(camk2)	 Pkinase, 128 442 7.5e-67*	
ENSPTRP00000009430 	588	KIN1_SCHPO/125-395(kinase)	 Pkinase, 165 446 5.3e-81*	
ENSPTRP00000009429 	545	KIN1_SCHPO/125-395(kinase)	 Pkinase, 165 444 3.8e-75*	
ENSPTRP00000017320 	433	SNF1(camk2)	 Pkinase, 49 309 8.8e-60*	
ENSPTRP00000007593 	476	SNF1(camk2)	 Pkinase, 9 265 7.8e-78*	
ENSPTRP00000037557 	311	PIM1_HUMAN/38-290(kinase)	 Pkinase, 32 286 1.3e-71*	
ENSPTRP00000030947 	404	PIM1_HUMAN/38-290(kinase)	 Pkinase, 129 381 1.3e-94*	
ENSPTRP00000043649 	1330	KAB7_YEAST/1096-1354(kinase)	 Pkinase, 999 1258 5.5e-67*	
ENSPTRP00000055043 	346	cAPKb(agc1)	 Pkinase, 37 291 5.1e-88*	
ENSPTRP00000037059 	277	cAPKb(agc1)	 Pkinase, 49 277 1.3e-67*	
ENSPTRP00000038756 	318	EcAPKa(agc1)	 Pkinase, 49 263 1.3e-46*	
ENSPTRP00000042330 	398	cAPKb(agc1)	 Pkinase, 91 345 6.5e-95*	
ENSPTRP00000052142 	355	cAPKb(agc1)	 Pkinase, 48 302 6.5e-95*	
ENSPTRP00000052141 	322	cAPKb(agc1)	 Pkinase, 48 269 1.8e-75*	
ENSPTRP00000018062 	335	agc1(agc1)	 Pkinase, 28 282 5.0e-93*	
ENSPTRP00000035898 	351	cAPKb(agc1)	 Pkinase, 44 298 2.1e-92*	
ENSPTRP00000004325 	671	PKG-I(agc1)	 cNMP_binding, 121 206 3.3e-20* cNMP_binding, 239 330 3.0e-22* Pkinase, 360 619 8.8e-96*	
ENSPTRP00000004326 	686	PKG-I(agc1)	 cNMP_binding, 136 221 3.3e-20* cNMP_binding, 254 345 3.0e-22* Pkinase, 375 634 8.8e-96*	
ENSPTRP00000027855 	762	PKG-II(agc1)	 cNMP_binding, 186 271 3.1e-18* cNMP_binding, 304 396 2.3e-23* Pkinase, 453 711 1.9e-90*	
ENSPTRP00000023198 	427	Sgk(agc_other)	 Pkinase, 95 352 8.6e-92* Pkinase_C, 372 421 1.3e-12*	
ENSPTRP00000034767 	496	Sgk(agc_other)	 PX, 11 120 9.8e-27* Pkinase, 162 419 1.6e-88* Pkinase_C, 439 491 5.9e-12*	
ENSPTRP00000034768 	464	Sgk(agc_other)	 PX, 11 120 9.8e-27* Pkinase, 162 387 1.6e-53* Pkinase_C, 407 459 5.9e-12*	
ENSPTRP00000049596 	526	Sgk(agc_other)	 Pkinase, 193 450 2.0e-89* Pkinase_C, 470 522 2.4e-11*	
ENSPTRP00000031791 	445	Sgk(agc_other)	 Pkinase, 112 369 2.0e-89* Pkinase_C, 389 441 2.4e-11*	
ENSPTRP00000031790 	431	Sgk(agc_other)	 Pkinase, 98 355 2.0e-89* Pkinase_C, 375 427 2.4e-11*	
ENSPTRP00000050982 	479	agc3(agc3)	 PH, 6 107 3.7e-19* Pkinase, 148 405 1.7e-110* Pkinase_C, 425 477 8.5e-12*	
ENSPTRP00000018854 	481	agc3(agc3)	 PH, 6 108 3.5e-24* Pkinase, 152 409 1.3e-111* Pkinase_C, 429 479 1.0e-10*	
ENSPTRP00000011569 	379	MPhKg(camk1)	 PH, 6 108 5.1e-22* Pkinase, 150 371 4.9e-57*	
ENSPTRP00000001607 	984	1+pck(agc2)	 HR1, 47 119 1.1e-23* HR1, 136 213 1.9e-22* HR1, 217 294 6.0e-21* Pkinase, 657 916 6.1e-86* Pkinase_C, 936 983 1.2e-16*	
ENSPTRP00000048957 	941	1+pck(agc2)	 HR1, 37 110 4.8e-22* HR1, 126 203 7.2e-24* HR1, 213 290 7.0e-22* Pkinase, 614 873 4.8e-84* Pkinase_C, 893 940 2.6e-15*	
ENSPTRP00000036652 	869	1+pck(agc2)	 HR1, 18 90 6.5e-22* HR1, 105 177 2.6e-19* HR1, 178 255 1.2e-20* Pkinase, 539 798 5.4e-81* Pkinase_C, 818 865 1.4e-09*	
ENSPTRP00000013479 	635	bPKC(agc2)	 C1_1, 1 51 3.8e-16* C1_1, 64 116 4.9e-26* C2, 135 222 6.7e-35* Pkinase, 304 562 9.5e-76* Pkinase_C, 582 627 2.7e-13*	
ENSPTRP00000016236 	672	aPKC(agc2)	 C1_1, 37 89 8.5e-22* C1_1, 102 154 1.4e-24* C2, 173 260 2.9e-38* Pkinase, 339 597 1.8e-84* Pkinase_C, 617 662 2.2e-13*	
ENSPTRP00000019651 	668	agc2(agc2)	 C1_1, 36 88 5.4e-23* C2, 144 231 1.2e-32* Pkinase, 322 585 1.8e-79* Pkinase_C, 605 650 2.1e-12*	
ENSPTRP00000010886 	683	ePKC(agc2)	 C2, 12 102 6.6e-16* C1_1, 172 225 5.0e-21* C1_1, 246 298 4.6e-23* Pkinase, 355 614 3.8e-86* Pkinase_C, 634 680 3.0e-18*	
ENSPTRP00000020411 	736	1BCePKC(agc2)	 C2, 7 98 4.3e-08* C1_1, 169 222 1.1e-21* C1_1, 242 294 2.2e-24* Pkinase, 407 667 3.0e-87* Pkinase_C, 687 733 5.8e-14*	
ENSPTRP00000041216 	706	thPKC(agc2)	 C1_1, 160 212 1.9e-19* C1_1, 232 284 4.7e-26* Pkinase, 380 634 5.1e-85* Pkinase_C, 654 700 7.2e-17*	
ENSPTRP00000025952 	601	dPKC(agc2)	 C1_1, 156 208 5.6e-27* Pkinase, 274 528 1.0e-84* Pkinase_C, 548 594 3.4e-18*	
ENSPTRP00000026868 	587	iPKC(agc2)	 PB1, 16 99 1.5e-16* C1_1, 132 184 4.3e-21* Pkinase, 245 513 2.9e-88* Pkinase_C, 533 579 7.0e-13*	
ENSPTRP00000000076 	519	zPKC(agc2)	 PB1, 15 98 1.0e-25* Pkinase, 208 445 4.8e-72* Pkinase_C, 465 511 6.0e-13*	
ENSPTRP00000006810 	484	agc6(agc6)	 Pkinase, 67 330 7.1e-90* Pkinase_C, 350 395 9.5e-16*	
ENSPTRP00000006811 	296	agc6(agc6)	 Pkinase, 67 288 5.7e-53*	
ENSPTRP00000016085 	525	agc6(agc6)	 Pkinase, 91 352 1.4e-92* Pkinase_C, 372 417 2.8e-14*	
ENSPTRP00000011290 	767	agc6(agc6)	 Pkinase, 14 283 1.1e-87* Pkinase_C, 303 346 1.1e-12* Pkinase, 391 652 2.3e-88*	
ENSPTRP00000006610 	772	agc6(agc6)	 Pkinase, 33 301 6.6e-90* Pkinase_C, 321 365 1.8e-12* Pkinase, 411 674 3.4e-81*	
ENSPTRP00000037953 	716	2RSKN(agc6)	 Pkinase, 73 302 2.1e-78* Pkinase_C, 322 366 1.1e-09* Pkinase, 398 654 5.0e-101*	
ENSPTRP00000000680 	738	2RSKN(agc6)	 Pkinase, 71 322 2.1e-93* Pkinase_C, 344 388 1.0e-12* Pkinase, 421 678 1.8e-114*	
ENSPTRP00000049453 	742	2RSKN(agc6)	 Pkinase, 130 384 1.2e-92* Pkinase_C, 387 431 1.2e-12* Pkinase, 464 681 9.5e-84*	
ENSPTRP00000045305 	554	agc1(agc1)	 Pkinase, 80 340 6.3e-85*	
ENSPTRP00000046231 	530	agc1(agc1)	 Pkinase, 80 316 4.5e-65*	
ENSPTRP00000049774 	663	2bARK(agc4)	 RGS, 29 149 3.5e-30* Pkinase, 166 428 5.5e-98* PH, 534 627 7.5e-14*	
ENSPTRP00000006793 	669	1bARK(agc4)	 RGS, 34 154 4.2e-27* Pkinase, 171 433 8.5e-98* PH, 539 632 8.8e-18*	
ENSPTRP00000027312 	578	IT11(agc4)	 RGS, 52 171 8.7e-34* Pkinase, 187 449 5.0e-69*	
ENSPTRP00000005279 	527	5GRK(agc4)	 RGS, 52 158 1.2e-19* Pkinase, 155 417 9.7e-75*	
ENSPTRP00000026645 	553	6GRK(agc4)	 RGS, 55 175 1.0e-07* Pkinase, 191 454 5.4e-70*	
ENSPTRP00000005404 	400	DdK2(agc_other)	 Pkinase, 93 348 4.0e-58*	
ENSPTRP00000005403 	294	DdK2(agc_other)	 Pkinase, 31 285 1.1e-58*	
ENSPTRP00000029704 	358	cAPKg(agc1)	 Pkinase, 23 281 3.8e-72*	
ENSPTRP00000053729 	387	DdK2(agc_other)	 Pkinase, 23 271 2.2e-59*	
ENSPTRP00000001186 	1267	Mast205(agc_other)	 DUF1908, 199 476 3.0e-194* Pkinase, 512 785 2.1e-80* Pkinase_C, 803 848 9.8e-07* PDZ, 1104 1189 3.8e-04*	
ENSPTRP00000045381 	1174	Mast205(agc_other)	 DUF1908, 199 406 1.5e-109* Pkinase, 442 715 2.1e-80* Pkinase_C, 733 778 9.8e-07* PDZ, 1011 1096 3.8e-04*	
ENSPTRP00000001185 	787	Mast205(agc_other)	 DUF1908, 84 361 3.0e-194* Pkinase, 397 670 2.1e-80* Pkinase_C, 688 733 9.8e-07*	
ENSPTRP00000029005 	2136	Mast205(agc_other)	 Pkinase, 84 357 1.8e-79* PDZ, 654 740 1.3e-06*	
ENSPTRP00000018003 	1170	Mast205(agc_other)	 DUF1908, 59 338 8.9e-208* Pkinase, 374 647 5.7e-79* Pkinase_C, 665 710 1.3e-04* PDZ, 967 1052 3.5e-08*	
ENSPTRP00000018244 	1093	Mast205(agc_other)	 DUF1908, 1 162 2.7e-65* Pkinase, 197 470 4.2e-82*	
ENSPTRP00000004033 	878	Sgk(agc_other)	 Pkinase, 35 834 8.0e-38*	
ENSPTRP00000004034 	840	Sgk(agc_other)	 Pkinase, 35 291 1.3e-25*	
ENSPTRP00000019172 	644	DM(agc_other)	 Pkinase, 118 386 2.6e-58* Pkinase_C, 404 451 1.5e-06* DMPK_coil, 512 572 9.0e-25*	
ENSPTRP00000019171 	600	DM(agc_other)	 Pkinase, 71 339 2.6e-58* Pkinase_C, 357 409 8.6e-06* DMPK_coil, 470 530 9.0e-25*	571 590*	
ENSPTRP00000019170 	626	DM(agc_other)	 Pkinase, 97 365 2.6e-58* Pkinase_C, 383 435 8.6e-06* DMPK_coil, 496 556 9.0e-25*	597 616*	
ENSPTRP00000003469 	1653	DM(agc_other)	 Pkinase, 77 343 9.2e-62* Pkinase_C, 361 408 1.0e-14* DMPK_coil, 798 858 4.3e-33* C1_1, 952 1004 1.5e-13* PH, 1022 1140 7.5e-05* CNH, 1167 1438 7.9e-105*	
ENSPTRP00000051082 	1631	DM(agc_other)	 Pkinase, 77 343 9.2e-62* Pkinase_C, 361 408 1.0e-14* DMPK_coil, 798 858 4.3e-33* C1_1, 930 982 1.5e-13* PH, 1000 1118 7.5e-05* CNH, 1145 1416 7.9e-105*	
ENSPTRP00000003470 	1537	DM(agc_other)	 Pkinase, 77 343 9.2e-62* Pkinase_C, 361 408 1.0e-14* DMPK_coil, 717 777 4.3e-33* C1_1, 836 888 1.5e-13* PH, 906 1024 7.5e-05* CNH, 1051 1322 7.9e-105*	
ENSPTRP00000051080 	1590	DM(agc_other)	 Pkinase, 77 343 9.2e-62* Pkinase_C, 361 408 1.0e-14* DMPK_coil, 798 858 4.3e-33* C1_1, 889 941 1.5e-13* PH, 959 1077 7.5e-05* CNH, 1104 1375 7.9e-105*	
ENSPTRP00000011507 	1652	DM(agc_other)	 Pkinase, 17 283 5.3e-63* Pkinase_C, 301 348 1.1e-09* DMPK_coil, 819 880 3.2e-31* C1_1, 967 1019 5.7e-16* PH, 1037 1155 5.3e-05* CNH, 1182 1454 7.5e-103*	
ENSPTRP00000044216 	1406	DM(agc_other)	 Pkinase, 70 336 1.4e-65* Pkinase_C, 354 401 1.5e-12* C1_1, 759 811 6.5e-11* PH, 828 946 2.9e-10* CNH, 973 1246 2.5e-52*	
ENSPTRP00000016847 	1354	DM(agc_other)	 Pkinase, 76 338 4.5e-73* Pkinase_C, 358 403 9.8e-08* HR1, 458 542 3.7e-10* Rho_Binding, 948 1014 3.9e-37* PH, 1119 1317 1.1e-04*	
ENSPTRP00000020039 	1388	DM(agc_other)	 Pkinase, 92 354 3.9e-71* Pkinase_C, 374 419 3.3e-09* HR1, 475 559 1.2e-13* Rho_Binding, 978 1046 1.2e-41* PH, 1151 1258 7.9e-05* C1_1, 1261 1313 1.0e-04*	
ENSPTRP00000009705 	1088	agc_other(agc_other)	 UBA, 99 136 1.3e-04* Pkinase, 668 973 8.6e-68* Pkinase_C, 992 1046 1.0e-07*	
ENSPTRP00000031907 	1130	agc_other(agc_other)	 UBA, 101 138 1.7e-10* Pkinase, 705 1010 5.4e-74* Pkinase_C, 1029 1084 3.2e-08*	
ENSPTRP00000008170 	464	agc_other(agc_other)	 Pkinase, 90 383 1.0e-82* Pkinase_C, 401 447 2.4e-11*	
ENSPTRP00000030929 	465	agc_other(agc_other)	 Pkinase, 89 382 2.7e-69* Pkinase_C, 400 449 1.1e-10*	
ENSPTRP00000009370 	2027	DM(agc_other)	 Pkinase, 97 360 5.0e-59* Pkinase_C, 378 425 2.9e-11* ATG16, 919 1045 5.3e-03* C1_1, 1363 1414 1.0e-06* PH, 1444 1563 1.3e-12* CNH, 1593 1883 8.1e-98*	
ENSPTRP00000019871 	309	FUSE_DROME/4-254(kinase)	 Pkinase, 43 293 1.3e-84*	
ENSPTRP00000014877 	344	KAKT_MLVAT/171-429(kinase)	 Pkinase, 77 327 3.0e-85*	
ENSPTRP00000023474 	403	FUSE_DROME/4-254(kinase)	 Pkinase, 133 383 7.2e-87*	
ENSPTRP00000028937 	685	polo(polo)	 Pkinase, 82 334 4.1e-88* POLO_box, 510 573 1.5e-30* POLO_box, 606 677 1.9e-34*	
ENSPTRP00000001150 	427	4547_Tt_CK1-Unclassified(ck1)	 Pkinase, 1 169 2.3e-26* POLO_box, 348 418 7.0e-18*	
ENSPTRP00000013475 	603	polo(polo)	 Pkinase, 53 305 3.4e-88* POLO_box, 417 480 1.0e-32* POLO_box, 515 584 3.6e-38*	
ENSPTRP00000028220 	966	FUSE_DROME/4-254(kinase)	 Pkinase, 12 265 4.0e-87* Sak_Polo, 844 961 1.6e-97*	
ENSPTRP00000024020 	786	p78(camk2)	 Pkinase, 27 281 1.4e-99*	
ENSPTRP00000007339 	881	p78(camk2)	 Pkinase, 1 226 9.4e-79*	
ENSPTRP00000047628 	1284	p78(camk2)	 Pkinase, 29 280 3.7e-101*	
ENSPTRP00000024019 	682	p78(camk2)	 Pkinase, 42 293 1.7e-105* UBA, 313 349 1.6e-05* KA1, 633 682 1.2e-11*	
ENSPTRP00000019126 	445	p78(camk2)	 Pkinase, 42 323 1.2e-94* UBA, 343 379 1.6e-05*	
ENSPTRP00000019125 	618	p78(camk2)	 Pkinase, 42 293 1.7e-105* UBA, 313 349 1.6e-05*	
ENSPTRP00000042756 	752	p78(camk2)	 Pkinase, 56 330 6.1e-95* UBA, 350 386 8.8e-05* KA1, 703 752 7.0e-24*	
ENSPTRP00000041728 	776	p78(camk2)	 Pkinase, 56 330 6.1e-95* UBA, 350 386 8.8e-05* KA1, 727 776 7.0e-24*	
ENSPTRP00000040804 	744	p78(camk2)	 Pkinase, 56 307 1.4e-105* UBA, 327 363 8.8e-05* KA1, 695 744 7.0e-24*	
ENSPTRP00000003352 	758	p78(camk2)	 Pkinase, 60 289 2.2e-80* UBA, 309 345 1.3e-05* KA1, 709 758 1.1e-25*	
ENSPTRP00000003354 	795	p78(camk2)	 Pkinase, 60 311 1.6e-103* UBA, 331 367 1.3e-05* KA1, 746 795 1.1e-25*	
ENSPTRP00000028863 	436	CDR1_SCHPO/12-258(kinase)	 Pkinase, 74 325 1.6e-86*	
ENSPTRP00000025528 	782	p78(camk2)	 Pkinase, 33 286 3.2e-96*	
ENSPTRP00000009145 	661	SNF1(camk2)	 Pkinase, 55 306 5.0e-107*	
ENSPTRP00000003178 	602	SNF1(camk2)	 Pkinase, 53 303 2.6e-100*	
ENSPTRP00000048213 	565	AKIN10(camk2)	 Pkinase, 18 285 2.8e-96*	
ENSPTRP00000048210 	207	KIN1_SCHPO/125-395(kinase)	 Pkinase, 18 203 3.0e-45*	
ENSPTRP00000001359 	552	AKIN10(camk2)	 Pkinase, 16 268 6.8e-99*	
ENSPTRP00000035809 	651	p78(camk2)	 Pkinase, 11 263 2.8e-97* KA1, 602 651 5.8e-23*	
ENSPTRP00000024203 	358	AKIN10(camk2)	 Pkinase, 12 272 2.2e-74*	
ENSPTRP00000000847 	268	AKIN10(camk2)	 Pkinase, 10 265 1.3e-80*	
ENSPTRP00000010533 	338	SNF1(camk2)	 Pkinase, 25 303 1.3e-65*	
ENSPTRP00000023839 	714	1+kin(camk2)	 Pkinase, 62 320 1.5e-89*	
ENSPTRP00000043392 	513	IIaCaMK(camk1)	 Pkinase, 116 380 5.2e-88*	
ENSPTRP00000049753 	586	DdMLCK(camk1)	 FHA, 156 235 5.3e-13* Pkinase, 263 529 2.2e-92*	
ENSPTRP00000024456 	543	DdMLCK(camk1)	 FHA, 113 192 5.3e-13* Pkinase, 220 486 2.2e-92*	
ENSPTRP00000049751 	452	DdMLCK(camk1)	 Pkinase, 129 395 2.2e-92*	
ENSPTRP00000010577 	914	mPKC(agc2)	 C1_1, 147 199 1.3e-16* C1_1, 271 323 1.4e-19* PH, 423 541 1.6e-12* Pkinase, 583 841 4.8e-81*	
ENSPTRP00000020330 	890	mPKC(agc2)	 C1_1, 155 207 5.2e-17* C1_1, 272 324 1.1e-17* PH, 417 532 1.1e-10* Pkinase, 574 832 1.5e-74*	
ENSPTRP00000019204 	732	mPKC(agc2)	 C1_1, 59 111 9.3e-17* C1_1, 185 237 4.4e-18* Pkinase, 405 661 3.0e-79*	
ENSPTRP00000017368 	355	PSK-H1(camk1)	 Pkinase, 83 309 3.0e-26*	
ENSPTRP00000017370 	406	PSK-H1(camk1)	 Pkinase, 83 309 3.0e-26*	
ENSPTRP00000001211 	465	PSK-H1(camk1)	 Pkinase, 49 374 9.0e-72*	
ENSPTRP00000001210 	424	PSK-H1(camk1)	 Pkinase, 49 333 2.1e-76*	
ENSPTRP00000001213 	329	K6A1_MOUSE/407-664(kinase)	 Pkinase, 1 238 2.3e-33*	
ENSPTRP00000003207 	400	MAPKAP2(camk_other)	 Pkinase, 64 325 1.6e-80*	
ENSPTRP00000025842 	382	MAPKAP2(camk_other)	 Pkinase, 43 304 1.6e-77*	
ENSPTRP00000009277 	461	IVCaMK(camk1)	 Pkinase, 10 292 1.6e-77*	
ENSPTRP00000013693 	406	TPhKg(camk1)	 Pkinase, 24 291 2.0e-112*	
ENSPTRP00000032825 	359	MPhKg(camk1)	 Pkinase, 1 260 8.0e-90*	
ENSPTRP00000004591 	534	IIgCaMK(camk1)	 Pkinase, 1 250 1.2e-88* CaMKII_AD, 402 529 2.7e-87*	
ENSPTRP00000004592 	505	IIgCaMK(camk1)	 Pkinase, 1 250 1.2e-88* CaMKII_AD, 373 500 2.7e-87*	
ENSPTRP00000004589 	507	IIgCaMK(camk1)	 Pkinase, 1 250 1.2e-88* CaMKII_AD, 375 502 2.7e-87*	
ENSPTRP00000032700 	505	IIbCaMK(camk1)	 Pkinase, 14 272 2.5e-96* CaMKII_AD, 373 500 8.3e-87*	
ENSPTRP00000032705 	480	IIbCaMK(camk1)	 Pkinase, 14 272 2.5e-96* CaMKII_AD, 348 475 8.3e-87*	
ENSPTRP00000032699 	456	IIbCaMK(camk1)	 Pkinase, 14 272 2.5e-96* CaMKII_AD, 324 451 8.3e-87*	
ENSPTRP00000032702 	455	IIbCaMK(camk1)	 Pkinase, 14 272 2.5e-96* CaMKII_AD, 349 450 4.5e-53*	
ENSPTRP00000029757 	455	IIaCaMK(camk1)	 Pkinase, 13 251 1.3e-83* CaMKII_AD, 323 450 5.6e-93*	
ENSPTRP00000028140 	499	IIdCaMK(camk1)	 Pkinase, 14 272 9.5e-102* CaMKII_AD, 346 473 1.9e-94*	
ENSPTRP00000032698 	344	IIdCaMK(camk1)	 Pkinase, 14 272 3.5e-102*	
ENSPTRP00000047442 	905	IIgCaMK(camk1)	 Pkinase, 1 255 1.4e-77* L27, 325 380 1.1e-18* L27, 384 437 1.5e-19* PDZ, 469 547 6.3e-22* SH3_2, 595 659 8.1e-16* Guanylate_kin, 754 858 2.5e-48*	
ENSPTRP00000047441 	876	IIgCaMK(camk1)	 Pkinase, 1 255 1.4e-77* L27, 319 374 1.1e-18* L27, 378 431 1.5e-19* PDZ, 463 541 6.3e-22* SH3_2, 566 630 8.1e-16* Guanylate_kin, 725 829 2.5e-48*	
ENSPTRP00000014082 	424	PSK-H1(camk1)	 Pkinase, 98 355 2.9e-94*	
ENSPTRP00000003885 	385	ICamK(camk1)	 Pkinase, 23 279 2.1e-105*	
ENSPTRP00000025169 	370	ICamK(camk1)	 Pkinase, 20 276 7.0e-104*	
ENSPTRP00000003266 	476	PSK-H1(camk1)	 Pkinase, 23 277 8.6e-107*	
ENSPTRP00000038601 	317	ICamK(camk1)	 Pkinase, 2 244 2.8e-63*	
ENSPTRP00000029317 	473	IVCaMK(camk1)	 Pkinase, 46 300 1.2e-117*	
ENSPTRP00000009841 	740	ICamK(camk1)	 DCX, 74 138 4.1e-25* DCX, 203 264 4.9e-26* Pkinase, 390 647 8.1e-101*	
ENSPTRP00000009840 	729	ICamK(camk1)	 DCX, 74 138 4.1e-25* DCX, 203 264 4.9e-26* Pkinase, 390 647 8.1e-101*	
ENSPTRP00000028331 	783	DmCamKII(camk1)	 DCX, 89 153 2.2e-26* DCX, 214 275 1.9e-24* Pkinase, 411 668 8.3e-94*	
ENSPTRP00000025422 	608	ICamK(camk1)	 Pkinase, 316 573 8.2e-106*	
ENSPTRP00000013797 	779	M-MLCK(camk1)	 Pkinase, 475 730 2.0e-85*	
ENSPTRP00000030173 	389	M-MLCK(camk1)	 Pkinase, 106 361 5.7e-88*	
ENSPTRP00000022910 	596	K-MLCK(camk1)	 Pkinase, 285 540 3.2e-83*	
ENSPTRP00000046634 	1914	M-MLCK(camk1)	 I-set, 33 123 5.5e-33* I-set, 161 250 4.8e-33* I-set, 414 504 7.7e-29* I-set, 514 600 2.3e-24* I-set, 623 712 4.8e-30* I-set, 721 811 1.1e-18* I-set, 1098 1187 3.7e-26* I-set, 1238 1327 4.5e-20* fn3, 1331 1416 1.9e-21* Pkinase, 1464 1719 2.7e-103* I-set, 1809 1899 4.9e-48*	
ENSPTRP00000042821 	1847	Titen(camk1)	 I-set, 33 123 5.5e-33* I-set, 161 250 4.8e-33* I-set, 414 504 7.7e-29* I-set, 514 600 2.3e-24* I-set, 623 712 4.8e-30* I-set, 721 811 1.1e-18* I-set, 1098 1187 3.7e-26* I-set, 1238 1327 4.5e-20* Pkinase, 1402 1652 8.9e-54* I-set, 1742 1832 4.9e-48*	
ENSPTRP00000026393 	1845	M-MLCK(camk1)	 I-set, 33 123 5.5e-33* I-set, 161 250 4.8e-33* I-set, 445 531 2.3e-24* I-set, 554 643 4.8e-30* I-set, 652 742 1.1e-18* I-set, 1029 1118 3.7e-26* I-set, 1169 1258 4.5e-20* fn3, 1262 1347 1.9e-21* Pkinase, 1395 1650 2.7e-103* I-set, 1740 1830 4.9e-48*	
ENSPTRP00000045642 	1864	M-MLCK(camk1)	 I-set, 33 123 5.5e-33* I-set, 161 250 4.8e-33* I-set, 414 504 7.7e-29* I-set, 514 600 2.3e-24* I-set, 623 712 4.8e-30* I-set, 721 811 1.1e-18* I-set, 1098 1187 3.7e-26* I-set, 1238 1327 4.5e-20* fn3, 1331 1416 1.9e-21* Pkinase, 1465 1669 6.8e-66* I-set, 1759 1849 4.9e-48*	
ENSPTRP00000049246 	1455	M-MLCK(camk1)	 Pkinase, 13 275 6.9e-92* Ank, 378 410 7.5e-06* Ank, 444 476 7.7e-11* Ank, 477 509 3.4e-08* Ank, 510 542 2.8e-09* Ank, 543 575 2.7e-07* Ank, 576 608 2.7e-07* Ank, 609 641 3.4e-07* Death, 1336 1421 5.0e-20*	
ENSPTRP00000041316 	1431	M-MLCK(camk1)	 Pkinase, 13 275 6.9e-92* Ank, 378 410 7.5e-06* Ank, 444 476 5.9e-03* Ank, 478 510 3.4e-08* Ank, 511 543 2.8e-09* Ank, 544 576 2.7e-07* Ank, 577 609 2.7e-07* Ank, 610 642 3.4e-07* Death, 1312 1397 5.0e-20*	
ENSPTRP00000017483 	454	IIdCaMK(camk1)	 Pkinase, 13 275 6.9e-93*	
ENSPTRP00000012230 	370	M-MLCK(camk1)	 Pkinase, 23 285 6.1e-88*	
ENSPTRP00000032664 	414	DdMLCK(camk1)	 Pkinase, 61 321 1.2e-80*	
ENSPTRP00000021817 	372	DdMLCK(camk1)	 Pkinase, 33 293 5.4e-75*	
ENSPTRP00000021693 	4647	Titen(camk1)	 fn3, 67 153 6.0e-14* fn3, 164 255 1.7e-14* I-set, 272 363 1.9e-10* fn3, 367 450 2.5e-06* fn3, 464 551 7.3e-16* fn3, 566 653 3.3e-19* I-set, 670 758 3.3e-17* fn3, 762 847 1.9e-10* fn3, 859 945 2.0e-18* I-set, 962 1052 3.3e-10* fn3, 1056 1142 6.1e-16* fn3, 1156 1242 3.1e-18* fn3, 1257 1343 2.4e-22* I-set, 1360 1448 2.3e-07* fn3, 1452 1540 5.7e-14* fn3, 1553 1640 4.1e-20* fn3, 1655 1741 1.8e-18* I-set, 1757 1846 1.3e-27* I-set, 1850 1943 8.3e-04* fn3, 1947 2031 3.0e-12* fn3, 2049 2135 3.7e-15* I-set, 2152 2243 3.0e-22* I-set, 2252 2342 7.5e-09* fn3, 2346 2430 2.8e-16* Pkinase, 2475 2729 8.3e-58* I-set, 2792 2882 1.9e-07* I-set, 2914 3006 3.4e-28* I-set, 3019 3109 5.7e-24* I-set, 3598 3687 2.1e-12* I-set, 3785 3874 9.3e-37* I-set, 3942 4030 3.3e-09* I-set, 4076 4165 3.4e-30* I-set, 4263 4350 3.7e-05* I-set, 4358 4447 4.7e-34* I-set, 4553 4644 1.6e-23*	
ENSPTRP00000028715 	3017	DdMLCK(camk1)	 Spectrin, 165 285 2.6e-03* Spectrin, 287 393 9.3e-07* Spectrin, 513 617 9.4e-04* Spectrin, 854 959 8.8e-05* Spectrin, 1085 1191 7.8e-09* RhoGEF, 1243 1413 9.4e-30* PH, 1427 1538 1.5e-14* SH3_1, 1606 1666 1.4e-05* RhoGEF, 1920 2091 9.2e-41* PH, 2105 2218 4.7e-09* I-set, 2605 2696 2.7e-24* Pkinase, 2716 2970 1.7e-66*	
ENSPTRP00000028713 	596	DdMLCK(camk1)	 I-set, 184 275 2.7e-24* Pkinase, 295 549 1.7e-66*	
ENSPTRP00000045109 	2924	DmCamKII(camk1)	 Spectrin, 126 246 1.7e-03* Spectrin, 248 354 5.0e-06* Spectrin, 474 580 3.0e-04* Spectrin, 828 942 1.4e-03* Spectrin, 1068 1174 4.5e-06* RhoGEF, 1223 1393 3.3e-32* PH, 1413 1518 2.2e-11* SH3_1, 1587 1647 2.7e-04* RhoGEF, 1871 2041 3.9e-40* PH, 2055 2164 1.5e-06* I-set, 2409 2503 6.5e-25* fn3, 2507 2592 7.1e-15* Pkinase, 2622 2876 6.5e-68*	
ENSPTRP00000026398 	1257	DmCamKII(camk1)	 RhoGEF, 204 374 3.9e-40* PH, 388 497 1.5e-06* I-set, 742 836 6.5e-25* fn3, 840 925 7.1e-15* Pkinase, 955 1209 6.5e-68*	
ENSPTRP00000003516 	1213	TWITCH(camk1)	 I-set, 810 900 4.8e-18* Pkinase, 978 1199 2.5e-17*	
ENSPTRP00000040692 	427	Jnk1(cmgc2)	 Pkinase, 26 321 2.2e-75*	
ENSPTRP00000027906 	426	Jnk1(cmgc2)	 Pkinase, 26 321 1.1e-75*	
ENSPTRP00000030121 	424	Jnk1(cmgc2)	 Pkinase, 26 321 1.0e-75*	
ENSPTRP00000030913 	360	HOG1(cmgc2)	 Pkinase, 24 308 1.5e-86*	
ENSPTRP00000030914 	307	HOG1(cmgc2)	 Pkinase, 24 271 8.2e-66*	
ENSPTRP00000030916 	365	1FpMPK(cmgc2)	 Pkinase, 25 308 1.4e-88*	
ENSPTRP00000044900 	312	Jnk1(cmgc2)	 Pkinase, 25 255 5.9e-78*	
ENSPTRP00000030917 	257	prp1+(cmgc5)	 Pkinase, 25 256 3.0e-34*	
ENSPTRP00000025056 	218	HOG1(cmgc2)	 Pkinase, 1 202 9.1e-29*	
ENSPTRP00000053615 	359	1ERK(cmgc2)	 Pkinase, 14 310 2.4e-81*	
ENSPTRP00000053614 	315	1ERK(cmgc2)	 Pkinase, 14 266 3.4e-75*	
ENSPTRP00000024313 	360	cmgc2(cmgc2)	 Pkinase, 25 313 3.4e-88*	
ENSPTRP00000015133 	793	7FpMPK(cmgc2)	 Pkinase, 55 347 6.8e-91*	
ENSPTRP00000015201 	515	6FpMPK(cmgc2)	 Pkinase, 126 415 3.3e-83*	
ENSPTRP00000017040 	555	3ERK(cmgc2)	 Pkinase, 20 311 2.5e-95*	
ENSPTRP00000012102 	721	3ERK(cmgc2)	 Pkinase, 20 316 1.9e-89*	
ENSPTRP00000032646 	1512	CHED(cmgc_other)	 Pkinase, 705 998 7.6e-98*	
ENSPTRP00000036573 	490	KIN28(cmgc1)	 Pkinase, 137 433 2.9e-96*	
ENSPTRP00000014478 	322	Dcdrk(cmgc_other)	 Pkinase, 39 288 4.6e-67*	
ENSPTRP00000014476 	276	Dcdrk(cmgc_other)	 Pkinase, 10 259 4.6e-67*	
ENSPTRP00000000061 	711	GTAp58(cmgc_other)	 Pkinase, 354 639 8.7e-88*	
ENSPTRP00000000063 	699	GTAp58(cmgc_other)	 Pkinase, 342 627 8.7e-88*	
ENSPTRP00000008632 	264	2Cdk(cmgc1)	 Pkinase, 4 252 1.1e-73*	
ENSPTRP00000008631 	298	2Cdk(cmgc1)	 Pkinase, 4 286 6.5e-117*	
ENSPTRP00000016424 	305	3Cdk(cmgc1)	 Pkinase, 4 286 4.7e-112*	
ENSPTRP00000004353 	297	CDC2Hs(cmgc1)	 Pkinase, 4 287 1.4e-111*	
ENSPTRP00000034032 	294	5Cdk(cmgc1)	 Pkinase, 4 288 5.2e-97*	
ENSPTRP00000009043 	523	2PCTAIRE(cmgc1)	 Pkinase, 192 473 2.4e-97*	
ENSPTRP00000037496 	496	1PCTAIRE(cmgc1)	 Pkinase, 165 446 1.4e-96*	
ENSPTRP00000042674 	500	3PCTAIRE(cmgc1)	 Pkinase, 174 451 6.9e-82*	
ENSPTRP00000033165 	469	3PCTAIRE(cmgc1)	 Pkinase, 135 419 4.0e-81*	
ENSPTRP00000033166 	453	3PCTAIRE(cmgc1)	 Pkinase, 119 403 4.0e-81*	
ENSPTRP00000021901 	384	3PCTAIRE(cmgc1)	 Pkinase, 52 336 3.8e-77*	
ENSPTRP00000048865 	345	KIN28(cmgc1)	 Pkinase, 103 325 2.8e-50*	
ENSPTRP00000039845 	303	cmgc1(cmgc1)	 Pkinase, 6 295 1.2e-82*	
ENSPTRP00000033192 	326	6Cdk(cmgc1)	 Pkinase, 13 300 4.3e-90*	
ENSPTRP00000036039 	399	3PCTAIRE(cmgc1)	 Pkinase, 1 216 1.5e-11*	
ENSPTRP00000036043 	295	3PCTAIRE(cmgc1)	 Pkinase, 1 237 2.3e-34*	
ENSPTRP00000029020 	346	CAK/MO15(cmgc1)	 Pkinase, 12 295 2.8e-105*	
ENSPTRP00000009752 	464	FpCdc2(cmgc1)	 Pkinase, 20 335 1.7e-72*	
ENSPTRP00000031595 	459	PHO85(cmgc1)	 Pkinase, 1 292 3.2e-58*	
ENSPTRP00000026315 	429	3bGSK(cmgc3)	 Pkinase, 56 349 1.2e-99*	
ENSPTRP00000018979 	483	3bGSK(cmgc3)	 Pkinase, 119 403 6.3e-105*	
ENSPTRP00000010728 	358	KKIALRE(cmgc_other)	 Pkinase, 5 288 3.8e-121*	
ENSPTRP00000052022 	314	KKIALRE(cmgc_other)	 Pkinase, 4 285 4.3e-105*	
ENSPTRP00000029494 	592	KKIALRE(cmgc_other)	 Pkinase, 4 286 3.2e-94*	
ENSPTRP00000037204 	959	KKIALRE(cmgc_other)	 Pkinase, 13 296 1.3e-79*	
ENSPTRP00000031242 	634	MAK(cmgc_other)	 Pkinase, 4 284 6.4e-110*	
ENSPTRP00000042420 	623	MAK(cmgc_other)	 Pkinase, 4 284 5.8e-110*	
ENSPTRP00000052464 	419	MHK(cmgc_other)	 Pkinase, 4 285 6.6e-78*	
ENSPTRP00000022508 	363	DdCKIIa(cmgc4)	 Pkinase, 39 321 2.1e-94*	
ENSPTRP00000053525 	315	a'CKII(cmgc4)	 Pkinase, 5 290 7.4e-95*	
ENSPTRP00000023923 	754	H2-PSK(cmgc5)	 Pkinase, 150 470 1.6e-61*	
ENSPTRP00000018823 	601	H2-PSK(cmgc5)	 Pkinase, 111 403 1.9e-62*	
ENSPTRP00000018824 	629	YAK1(cmgc5)	 Pkinase, 111 431 3.2e-63*	
ENSPTRP00000008819 	578	H2-PSK(cmgc5)	 Pkinase, 199 512 1.3e-70*	
ENSPTRP00000003206 	588	H2-PSK(cmgc5)	 Pkinase, 209 522 4.8e-80*	
ENSPTRP00000007788 	520	H2-PSK(cmgc5)	 Pkinase, 104 400 3.7e-66*	
ENSPTRP00000018861 	616	YAK1(cmgc5)	 Pkinase, 11 347 9.1e-54*	
ENSPTRP00000045357 	541	YAK1(cmgc5)	 Pkinase, 11 272 1.1e-56*	
ENSPTRP00000006052 	1215	H2-PSK(cmgc5)	 Pkinase, 197 525 8.8e-58*	
ENSPTRP00000048177 	1194	H2-PSK(cmgc5)	 Pkinase, 197 525 8.8e-58*	
ENSPTRP00000033846 	1191	H2-PSK(cmgc5)	 Pkinase, 192 520 1.5e-60*	
ENSPTRP00000047612 	913	H2-PSK(cmgc5)	 Pkinase, 192 520 1.5e-60*	
ENSPTRP00000001943 	1214	H2-PSK(cmgc5)	 Pkinase, 190 520 7.7e-54*	
ENSPTRP00000001941 	1169	H2-PSK(cmgc5)	 Pkinase, 190 520 3.0e-53*	
ENSPTRP00000030194 	374	prp1+(cmgc5)	 Pkinase, 176 371 3.2e-08*	
ENSPTRP00000030195 	1007	YAK1(cmgc5)	 Pkinase, 687 1003 6.6e-43*	
ENSPTRP00000035593 	566	G1-PSK(cmgc5)	 Pkinase, 232 548 2.0e-66*	
ENSPTRP00000012458 	467	G1-PSK(cmgc5)	 Pkinase, 142 449 6.8e-50*	
ENSPTRP00000012456 	490	G1-PSK(cmgc5)	 Pkinase, 156 472 2.0e-66*	
ENSPTRP00000051728 	499	G1-PSK(cmgc5)	 Pkinase, 163 479 1.1e-73*		
ENSPTRP00000021858 	484	cmgc5(cmgc5)	 Pkinase, 161 477 1.3e-90*		
ENSPTRP00000043617 	455	cmgc5(cmgc5)	 Pkinase, 132 448 2.7e-88*		
ENSPTRP00000030075 	481	cmgc5(cmgc5)	 Pkinase, 159 475 1.1e-82*		
ENSPTRP00000020055 	343	IIdCaMK(camk1)	 Pkinase, 64 308 1.4e-25*		
ENSPTRP00000035184 	372	nim1+(camk2)	 Pkinase, 104 338 1.2e-19*		
ENSPTRP00000022498 	356	nim1+(camk2)	 Pkinase, 71 313 6.0e-11*		
ENSPTRP00000003319 	1066	agc6(agc6)	 PX, 9 128 7.2e-14* MIT, 239 307 6.4e-24* Pkinase, 838 1056 1.6e-09*		
ENSPTRP00000042372 	1036	SPK1_YEAST/198-466(kinase)	 Pkinase, 9 271 3.0e-82*		
ENSPTRP00000009595 	860	KCC4_MOUSE/42-296(kinase)	 Pkinase, 1 196 2.3e-30*		
ENSPTRP00000012469 	257	SPK1_YEAST/198-466(kinase)	 Pkinase, 57 255 3.2e-46*		
ENSPTRP00000022091 	1297	FUSE_DROME/4-254(kinase)	 Pkinase, 4 254 7.9e-104*		
ENSPTRP00000008798 	729	K-MLCK(camk1)	 Pkinase, 9 301 4.5e-39*		
ENSPTRP00000003199 	592	MKK1_YEAST/221-488(kinase)	 Pkinase, 9 292 1.9e-32*		
ENSPTRP00000034602 	738	SPK1_YEAST/198-466(kinase)	 Pkinase, 15 312 3.2e-49*		
ENSPTRP00000034600 	483	1KIN(camk2)	 Pkinase, 1 263 1.1e-18*		
ENSPTRP00000016137 	772	IIdCaMK(camk1)	 Pkinase, 462 741 1.1e-68*		
ENSPTRP00000016134 	750	IIdCaMK(camk1)	 Pkinase, 440 719 1.1e-68*		
ENSPTRP00000016135 	718	IIdCaMK(camk1)	 Pkinase, 408 687 1.1e-68*		
ENSPTRP00000042220 	787	DdMLCK(camk1)	 Pkinase, 477 755 9.3e-66*		
ENSPTRP00000040410 	766	DdMLCK(camk1)	 Pkinase, 456 734 9.3e-66*		
ENSPTRP00000016665 	365	HRR25(ck1)	 Pkinase, 9 223 6.2e-18*		
ENSPTRP00000040515 	416	HRR25(ck1)	 Pkinase, 9 273 1.7e-43*		
ENSPTRP00000029734 	336	HRR25(ck1)	 Pkinase, 17 281 3.5e-34*		
ENSPTRP00000047919 	364	HRR25(ck1)	 Pkinase, 17 309 6.3e-33*		
ENSPTRP00000009862 	337	HRR25(ck1)	 Pkinase, 17 281 4.7e-33*		
ENSPTRP00000012237 	422	CKId(ck1)	 Pkinase, 44 311 3.3e-38*		
ENSPTRP00000012238 	393	CKId(ck1)	 Pkinase, 44 311 3.3e-38*		
ENSPTRP00000054505 	455	YCK2(ck1)	 Pkinase, 43 308 3.2e-37*		
ENSPTRP00000046543 	423	YCK2(ck1)	 Pkinase, 43 308 3.2e-37*		
ENSPTRP00000017363 	353	CK1g3_Hs_CK1-G(ck1)	 Pkinase, 46 352 6.4e-11*		
ENSPTRP00000011923 	1244	TTBK2_Mm(ck1)	 Pkinase, 21 279 9.3e-21*		
ENSPTRP00000011929 	1651	TTBK2_Mm(ck1)	 Pkinase, 13 278 1.0e-08* Filament, 343 592 7.4e-58*		
ENSPTRP00000031088 	1320	TTBK2_Hs(ck1)	 Pkinase, 34 293 2.9e-23*		
ENSPTRP00000031089 	621	TTBK2_Hs(ck1)	 Pkinase, 1 242 1.7e-10*		
ENSPTRP00000011431 	396	VRK1_Hs(ck1)	 Pkinase, 37 303 9e-07*		
ENSPTRP00000020517 	484	VRK2_Hs(ck1)	 Pkinase, 29 268 8.4e-07*	463 482*	
ENSPTRP00000015250 	622	Nek1(nima)	 Pkinase, 4 284 1.6e-61* RCC1, 509 558 2.0e-05*		
ENSPTRP00000003293 	445	KIN3(nima)	 Pkinase, 8 271 1.6e-73*		
ENSPTRP00000028467 	1261	Nek1(nima)	 Pkinase, 4 258 2.3e-88*		
ENSPTRP00000028468 	1217	Nek1(nima)	 Pkinase, 4 258 2.3e-88*		
ENSPTRP00000045541 	1289	Nek1(nima)	 Pkinase, 4 258 2.3e-88*		
ENSPTRP00000040121 	708	Nek1(nima)	 Pkinase, 4 259 1.1e-82*		
ENSPTRP00000010028 	507	Nek1(nima)	 Pkinase, 4 255 9.7e-87*		
ENSPTRP00000025935 	841	Nek1(nima)	 Pkinase, 6 261 2.4e-82*		
ENSPTRP00000025934 	781	Nek1(nima)	 Pkinase, 6 261 2.4e-82*		
ENSPTRP00000026525 	647	Nek1(nima)	 Pkinase, 29 287 6.0e-83*		
ENSPTRP00000048967 	347	Nek1(nima)	 Pkinase, 79 340 4.6e-80*		
ENSPTRP00000048966 	245	NrkA(nima)	 Pkinase, 15 238 3.7e-46*		
ENSPTRP00000003028 	302	NrkA(nima)	 Pkinase, 34 291 1.4e-77*		
ENSPTRP00000011128 	937	Nek1(nima)	 Pkinase, 52 266 6.4e-29* RCC1, 402 453 8.3e-05* RCC1, 458 505 2.2e-04*		
ENSPTRP00000013108 	499	wee1(wee1)	 Pkinase, 110 359 5.9e-51*		
ENSPTRP00000005852 	646	HsWee1(wee1)	 Pkinase, 299 569 4.3e-76*		
ENSPTRP00000011798 	1649	GCN2(translationk)	 RWD, 18 134 2.3e-33* Pkinase, 315 539 1.4e-10* Pkinase, 590 1001 9.5e-53* tRNA-synt_2b, 1063 1223 3.4e-03*		
ENSPTRP00000011800 	1621	GCN2(translationk)	 RWD, 18 134 2.3e-33* Pkinase, 315 539 1.4e-10* Pkinase, 590 973 3.7e-51* tRNA-synt_2b, 1035 1195 3.4e-03*		
ENSPTRP00000020876 	1115	PKR(translationk)	 Pkinase, 592 1071 2.9e-38*	7 29*	
ENSPTRP00000032290 	602	translationk(translationk)	 Pkinase, 167 555 1.3e-28*		
ENSPTRP00000020319 	552	PKR(translationk)	 dsrm, 10 75 8.5e-19* dsrm, 101 165 6.9e-14* Pkinase, 267 537 1.2e-62*		
ENSPTRP00000020323 	511	PKR(translationk)	 dsrm, 10 75 8.5e-19* dsrm, 101 165 6.9e-14* Pkinase, 171 496 6.6e-38*		
ENSPTRP00000053553 	677	CDC5(polo)	 Pkinase, 53 316 1.8e-34*		
ENSPTRP00000027232 	1311	polo_real(polo)	 Pkinase, 40 313 2.0e-52*		
ENSPTRP00000022716 	719	STE20(pak)	 PBD, 10 67 1.4e-21* Pkinase, 449 700 3.4e-85*		
ENSPTRP00000011806 	640	STE20(pak)	 PBD, 11 67 1.3e-19* Pkinase, 366 617 2.6e-81*		
ENSPTRP00000047028 	507	STE20(pak)	 PBD, 74 132 2.9e-31* Pkinase, 270 490 2.1e-70*		
ENSPTRP00000041645 	559	STE20(pak)	 PBD, 69 142 3.2e-26* Pkinase, 283 534 6.6e-100*		
ENSPTRP00000038191 	580	STE20(pak)	 PBD, 69 163 3.9e-22* Pkinase, 304 555 6.6e-100*		
ENSPTRP00000027164 	523	STE20(pak)	 PBD, 73 131 5.6e-31* Pkinase, 249 499 4.7e-95*		
ENSPTRP00000025450 	527	ST20_YEAST/620-871(kinase)	 Pkinase, 17 291 5.4e-71*		
ENSPTRP00000021530 	545	ST20_YEAST/620-871(kinase)	 Pkinase, 63 337 7.2e-69*		
ENSPTRP00000052114 	475	ST20_YEAST/620-871(kinase)	 Pkinase, 18 269 4.1e-93*		
ENSPTRP00000052113 	450	ST20_YEAST/620-871(kinase)	 Pkinase, 18 269 4.1e-93*		
ENSPTRP00000034983 	491	ST20_YEAST/620-871(kinase)	 Pkinase, 27 278 7.5e-94*		
ENSPTRP00000053410 	443	ST20_YEAST/620-871(kinase)	 Pkinase, 36 286 7.0e-94*		
ENSPTRP00000053409 	360	ST20_YEAST/620-871(kinase)	 Pkinase, 1 183 2.3e-39*		
ENSPTRP00000046906 	426	ST20_YEAST/620-871(kinase)	 Pkinase, 20 270 1.9e-93*		
ENSPTRP00000038373 	349	ST20_YEAST/620-871(kinase)	 Pkinase, 24 274 1.2e-92*		
ENSPTRP00000038378 	287	CC15_YEAST/25-272(kinase)	 Pkinase, 24 262 7.2e-49*		
ENSPTRP00000041788 	1217	NINC_DROME/16-282(kinase)	 Pkinase, 1 187 4.4e-22* CNH, 904 1195 6.7e-100*		
ENSPTRP00000014699 	1188	NINC_DROME/16-282(kinase)	 Pkinase, 1 187 4.4e-22* CNH, 875 1166 6.7e-100*		
ENSPTRP00000021061 	1224	NINC_DROME/16-282(kinase)	 Pkinase, 6 270 5.9e-84* CNH, 903 1202 1.7e-106*		
ENSPTRP00000021059 	1220	NINC_DROME/16-282(kinase)	 Pkinase, 6 270 5.9e-84* CNH, 907 1198 6.2e-110*		
ENSPTRP00000021060 	1302	NINC_DROME/16-282(kinase)	 Pkinase, 6 270 5.9e-84* CNH, 989 1280 6.2e-110*		
ENSPTRP00000021058 	1147	NINC_DROME/16-282(kinase)	 Pkinase, 6 270 5.9e-84* CNH, 826 1125 1.7e-106*		
ENSPTRP00000043904 	1360	NINC_DROME/16-282(kinase)	 Pkinase, 25 289 2.6e-85* CNH, 1047 1338 2.1e-99*		
ENSPTRP00000026879 	1331	NINC_DROME/16-282(kinase)	 Pkinase, 25 289 2.6e-85* CNH, 1018 1309 2.1e-99*		
ENSPTRP00000026882 	1305	NINC_DROME/16-282(kinase)	 Pkinase, 25 289 2.6e-85* CNH, 992 1283 2.1e-99*		
ENSPTRP00000038084 	1498	NINC_DROME/16-282(kinase)	 Pkinase, 25 313 6.3e-66* CNH, 1122 1472 1.3e-50*		
ENSPTRP00000042257 	1275	NINC_DROME/16-282(kinase)	 Pkinase, 27 293 1.9e-79* Myosin_head, 345 1046 8.0e-232* IQ, 1088 1108 4.1e-06*		
ENSPTRP00000021569 	1341	NINC_DROME/16-282(kinase)	 Pkinase, 27 293 1.9e-79* Myosin_head, 345 1046 8.0e-232* IQ, 1088 1108 4.1e-06*		
ENSPTRP00000021572 	1314	NINC_DROME/16-282(kinase)	 Pkinase, 27 293 1.9e-79* Myosin_head, 345 1046 8.0e-232*		
ENSPTRP00000004014 	1488	NINC_DROME/16-282(kinase)	 Pkinase, 21 287 7.5e-79* Myosin_head, 340 1041 1.5e-224* IQ, 1083 1103 7.9e-04* IQ, 1347 1367 5.2e-03*		
ENSPTRP00000020357 	879	ST20_YEAST/620-871(kinase)	 Pkinase, 16 273 2.3e-82* CNH, 562 859 5.8e-80*		
ENSPTRP00000020358 	858	ST20_YEAST/620-871(kinase)	 Pkinase, 16 273 2.3e-82* CNH, 541 838 5.8e-80*		
ENSPTRP00000010729 	845	ST20_YEAST/620-871(kinase)	 Pkinase, 20 277 2.7e-74* CNH, 512 825 1.0e-116*		
ENSPTRP00000006632 	820	ST20_YEAST/620-871(kinase)	 Pkinase, 16 273 5.9e-73* CNH, 488 800 3.4e-101*		
ENSPTRP00000018743 	778	ST20_YEAST/620-871(kinase)	 Pkinase, 17 254 1.1e-49* CNH, 481 778 5.4e-82*		
ENSPTRP00000005141 	1205	ST20_YEAST/620-871(kinase)	 Pkinase, 34 292 4.3e-92*		
ENSPTRP00000005139 	1236	ST20_YEAST/620-871(kinase)	 Pkinase, 34 292 4.3e-92*		
ENSPTRP00000029935 	957	ST20_YEAST/620-871(kinase)	 Pkinase, 36 277 3.6e-76*		
ENSPTRP00000013638 	1232	CC15_YEAST/25-272(kinase)	 Pkinase, 28 281 7.4e-76*	952 974*981 1003*1013 1031*1038 1060*	
ENSPTRP00000013639 	901	CC15_YEAST/25-272(kinase)	 Pkinase, 28 281 7.4e-76*		
ENSPTRP00000015275 	1001	CC15_YEAST/25-272(kinase)	 Pkinase, 28 281 1.6e-79*		
ENSPTRP00000049880 	830	CC15_YEAST/25-272(kinase)	 Pkinase, 24 277 1.7e-77*		
ENSPTRP00000004070 	467	ST20_YEAST/620-871(kinase)	 Pkinase, 136 388 2.2e-51*		
ENSPTRP00000016161 	628	NPK1(mekk_ste11)	 PB1, 75 154 8.2e-20* Pkinase, 393 624 4.6e-57*		
ENSPTRP00000016162 	597	NPK1(mekk_ste11)	 PB1, 44 123 8.2e-20* Pkinase, 362 593 4.6e-57*		
ENSPTRP00000021280 	618	NPK1(mekk_ste11)	 PB1, 43 122 2.7e-19* Pkinase, 355 615 1.2e-82*		
ENSPTRP00000044542 	1215	byr2(mekk_ste11)	 Pkinase, 948 1211 4.3e-81*		
ENSPTRP00000049168 	510	byr2(mekk_ste11)	 Pkinase, 243 506 4.3e-81*	
ENSPTRP00000049167 	1328	byr2(mekk_ste11)	 Pkinase, 1061 1324 4.3e-81*		
ENSPTRP00000032056 	1522	BYR2_SCHPO/394-658(kinase)	 Pkinase, 1257 1515 4.8e-77*		
ENSPTRP00000041330 	1568	BYR2_SCHPO/394-658(kinase)	 Pkinase, 1303 1561 4.8e-77*		
ENSPTRP00000042496 	558	BYR2_SCHPO/394-658(kinase)	 Pkinase, 43 265 1.1e-35*		
ENSPTRP00000037229 	705	BYR2_SCHPO/394-658(kinase)	 Pkinase, 309 559 6.7e-38*		
ENSPTRP00000044443 	1374	NPK1(mekk_ste11)	 Pkinase, 675 938 1.4e-77*		
ENSPTRP00000049582 	1252	NPK1(mekk_ste11)	 Pkinase, 755 1018 1.4e-77*		
ENSPTRP00000000706 	1288	NPK1(mekk_ste11)	 Pkinase, 649 906 6.9e-73*		
ENSPTRP00000048240 	1347	mekk_ste11(mekk_ste11)	 SWIM, 177 205 3.4e-05* Pkinase, 1078 1343 5.6e-88*		
ENSPTRP00000017496 	369	MEK2(mek_ste7)	 Pkinase, 41 338 4.9e-71*		
ENSPTRP00000012302 	393	mek(mek_ste7)	 Pkinase, 68 361 5.5e-73*		
ENSPTRP00000012335 	258	mek(mek_ste7)	 Pkinase, 4 229 2.2e-48*		
ENSPTRP00000012334 	474	mek(mek_ste7)	 PB1, 18 97 6.2e-15* Pkinase, 166 409 4.8e-61*		
ENSPTRP00000039533 	351	PBS2(mek_ste7)	 Pkinase, 68 329 5.1e-53*		
ENSPTRP00000039686 	334	PBS2(mek_ste7)	 Pkinase, 53 314 2.8e-56*		
ENSPTRP00000014968 	399	MEK2(mek_ste7)	 Pkinase, 102 367 9.6e-63*		
ENSPTRP00000017707 	441	MEK2(mek_ste7)	 Pkinase, 142 402 2.1e-60*		
ENSPTRP00000022925 	508	sp|Q95KR7|LCK_SAISC(PTK8)	 SH3_1, 81 136 9.1e-27* SH2, 144 226 1.9e-43* Pkinase, 262 493 7.0e-130*		
ENSPTRP00000046608 	512	sp|Q95KR7|LCK_SAISC(PTK8)	 SH3_1, 66 121 2.0e-26* SH2, 129 211 2.1e-41* Pkinase, 247 497 1.6e-153*		
ENSPTRP00000000842 	490	sp|Q95KR7|LCK_SAISC(PTK8)	 SH3_1, 26 81 9.4e-20* SH2, 89 160 8.8e-27* Pkinase, 196 475 3.2e-143*		
ENSPTRP00000045934 	467	sp|Q95KR7|LCK_SAISC(PTK8)	 SH3_1, 84 139 9.4e-20* SH2, 147 218 8.8e-27* Pkinase, 211 452 1.8e-132*		
ENSPTRP00000048521 	492	sp|Q95KR7|LCK_SAISC(PTK8)	 SH3_1, 61 116 2.3e-23* SH2, 124 205 5.0e-38* Pkinase, 228 477 4.3e-145*		
ENSPTRP00000039389 	492	ptk1(ptk1)	 SH3_1, 87 149 4.1e-21* SH2, 157 215 5.5e-05* Pkinase, 226 475 8.3e-151*		
ENSPTRP00000016724 	543	sp|Q95KR7|LCK_SAISC(PTK8)	 SH3_1, 94 150 7.7e-27* SH2, 158 240 2.4e-43* Pkinase, 277 526 2.0e-148*		
ENSPTRP00000054137 	482	sp|Q95KR7|LCK_SAISC(PTK8)	 SH3_1, 85 141 8.1e-30* SH2, 149 231 1.6e-44* Pkinase, 223 465 2.8e-136*		
ENSPTRP00000031615 	537	sp|Q95KR7|LCK_SAISC(PTK8)	 SH3_1, 85 141 8.1e-30* SH2, 149 231 1.6e-44* Pkinase, 271 520 9.9e-154*		
ENSPTRP00000031617 	489	FYN(ptk1)	 SH2, 104 186 1.6e-44* Pkinase, 223 472 8.1e-153*		
ENSPTRP00000000718 	465	c-Fgr(ptk1)	 SH3_1, 80 136 3.7e-28* SH2, 144 226 3.5e-46* Pkinase, 263 448 3.0e-68*		
ENSPTRP00000031633 	505	sp|Q95KR7|LCK_SAISC(PTK8)	 SH3_1, 45 108 1.3e-18* SH2, 116 193 1.1e-38* Pkinase, 234 487 5.1e-147*		
ENSPTRP00000023665 	249	Matk(ptk3)	 Pkinase, 27 239 1.1e-64*		
ENSPTRP00000023666 	489	sp|Q95KR7|LCK_SAISC(PTK8)	 SH3_2, 55 110 5.3e-04* SH2, 120 197 5.2e-27* Pkinase, 230 480 6.0e-138*		
ENSPTRP00000036748 	1149	ARG(ptk5)	 SH3_1, 83 138 2.6e-19* SH2, 146 221 1.4e-34* Pkinase, 261 512 9.3e-157* F_actin_bind, 972 1149 1.0e-140*		
ENSPTRP00000002874 	1064	ARG(ptk5)	 SH3_1, 95 150 4.5e-19* SH2, 158 233 1.7e-37* Pkinase, 273 524 3.9e-154* F_actin_bind, 902 1064 3.0e-122*		
ENSPTRP00000051478 	1182	ARG(ptk5)	 SH3_1, 110 165 4.5e-19* SH2, 173 248 1.7e-37* Pkinase, 288 539 3.9e-154* F_actin_bind, 1020 1182 3.0e-122*		
ENSPTRP00000038032 	659	ptk2(ptk2)	 PH, 4 133 1.2e-15* BTK, 135 171 2.7e-24* SH3_1, 217 272 6.1e-26* SH2, 281 362 6.9e-32* Pkinase, 402 651 7.2e-148*		
ENSPTRP00000047337 	208	Dsrc28(ptk2)	 Pkinase, 4 200 6.2e-65*	
ENSPTRP00000040876 	535	ptk2(ptk2)	 BTK, 78 114 6.1e-22* Pkinase, 279 526 2.3e-101*		
ENSPTRP00000029829 	620	Itk/Tsk(ptk2)	 PH, 5 111 2.5e-17* BTK, 113 149 2.5e-22* SH3_1, 174 229 1.8e-20* SH2, 239 323 1.6e-30* Pkinase, 363 612 8.0e-142*		
ENSPTRP00000027601 	631	ptk2(ptk2)	 PH, 5 111 1.8e-16* BTK, 113 149 6.8e-24* SH3_1, 182 237 1.9e-22* SH2, 247 330 1.1e-29* Pkinase, 370 619 4.4e-147*		
ENSPTRP00000027599 	478	sp|Q95KR7|LCK_SAISC(PTK8)	 SH3_1, 85 140 4.2e-20* SH2, 150 231 3.5e-35* Pkinase, 271 471 1.7e-89*		
ENSPTRP00000042453 	365	ptk3(ptk3)	 Pkinase, 96 336 5.1e-123*		
ENSPTRP00000017469 	364	ptk3(ptk3)	 Pkinase, 95 335 5.1e-123*		
ENSPTRP00000012464 	441	ptk3(ptk3)	 SH3_1, 12 68 1.5e-21* SH2, 82 156 3.0e-37* Pkinase, 195 431 8.1e-128*		
ENSPTRP00000012756 	822	ptk4(ptk4)	 FCH, 1 94 3.1e-30* SH2, 460 530 1.3e-27* Pkinase, 561 814 2.3e-149*		
ENSPTRP00000029300 	822	FER(ptk4)	 FCH, 1 92 9.7e-32* SH2, 460 531 1.9e-33* Pkinase, 563 814 9.8e-154*		
ENSPTRP00000017670 	1359	INS.R(ptk16)	 Recep_L_domain, 52 164 1.1e-44* Furin-like, 179 340 1.1e-95* Recep_L_domain, 359 474 1.5e-48* fn3, 831 914 1.1e-03* Pkinase, 1000 1267 5.1e-154*	9 31*934 956*	
ENSPTRP00000012792 	1364	ptk16(ptk16)	 Recep_L_domain, 51 161 4.8e-44* Furin-like, 175 333 1.5e-95* Recep_L_domain, 352 467 1.5e-46* Pkinase, 999 1266 2.2e-153*	936 958*	
ENSPTRP00000002493 	1292	ptk16(ptk16)	 Recep_L_domain, 47 159 2.8e-38* Furin-like, 173 329 2.2e-91* Recep_L_domain, 346 460 1.1e-30* Pkinase, 974 1241 2.8e-143*	917 939*	
ENSPTRP00000011851 	802	ALK(ptk17)	 Pkinase, 449 716 3.6e-136*	365 387*	
ENSPTRP00000011852 	863	ALK(ptk17)	 Pkinase, 510 777 3.6e-136*	426 448*	
ENSPTRP00000020259 	1429	ALK(ptk17)	 MAM, 436 592 7.5e-29* Pkinase, 1072 1326 9.3e-121*	993 1015*	
ENSPTRP00000031658 	2347	ptk18(ptk18)	 fn3, 99 183 8.7e-04* fn3, 194 277 3.9e-10* fn3, 558 661 4.6e-03* fn3, 1041 1140 2.6e-05* fn3, 1659 1745 5.6e-03* Pkinase, 1945 2215 3.4e-151*	1860 1882*	
ENSPTRP00000012676 	812	TrkC(ptk19)	 LRRNT, 4 31 8.7e-06* ig, 197 259 5.5e-07* Pkinase, 511 797 4.3e-137*	402 424*	
ENSPTRP00000036008 	838	TrkB(ptk19)	 LRRNT, 31 60 3.8e-07* I-set, 197 283 8.8e-10* Pkinase, 554 823 1.5e-148*	10 32*432 454*	
ENSPTRP00000002495 	760	ptk19(ptk19)	 I-set, 164 252 6.0e-03* Pkinase, 474 745 5.6e-140*	381 403*	
ENSPTRP00000002496 	796	ptk19(ptk19)	 I-set, 194 282 6.0e-03* Pkinase, 510 781 5.6e-140*	417 439*	
ENSPTRP00000049076 	875	TorRTK(ptk19)	 I-set, 28 117 2.5e-15* I-set, 121 208 7.7e-34* I-set, 212 299 5.6e-05* Fz, 317 454 3.1e-47* Pkinase, 581 862 1.3e-149*	502 524*644 666*	
ENSPTRP00000036065 	704	Ror2(ptk19)	 Fz, 28 161 3.1e-47* Kringle, 176 254 4.8e-34* Pkinase, 333 606 3.2e-135*	263 285*	
ENSPTRP00000036064 	928	Ror2(ptk19)	 I-set, 47 137 3.8e-21* Fz, 153 286 3.1e-47* Kringle, 301 379 4.8e-34* Pkinase, 458 731 3.2e-135*	388 410*	
ENSPTRP00000052236 	906	Ror1(ptk19)	 I-set, 27 117 2.8e-18* Fz, 133 266 6.7e-51* Kringle, 282 360 7.3e-34* Pkinase, 442 715 1.4e-142*	373 395*644 666*	
ENSPTRP00000030613 	876	ptk20(ptk20)	 F5_F8_type_C, 46 182 2.5e-32* Pkinase, 573 868 7.2e-122*	417 439*	
ENSPTRP00000030610 	913	ptk20(ptk20)	 F5_F8_type_C, 46 182 2.5e-32* Pkinase, 610 905 7.2e-122*	417 439*	
ENSPTRP00000002684 	855	TKT(ptk20)	 F5_F8_type_C, 45 182 3.8e-34* Pkinase, 563 849 1.9e-120*	399 421*	
ENSPTRP00000018925 	863	Ark(ptk12)	 ig, 49 119 3.1e-06* fn3, 225 321 3.8e-08* fn3, 334 418 6.4e-11* Pkinase, 527 772 2.1e-113*	441 463*	
ENSPTRP00000021171 	999	c-Eyk(ptk12)	 ig, 108 177 3.4e-06* I-set, 197 280 9.0e-03* fn3, 284 368 2.6e-08* Pkinase, 587 854 6.1e-141*	502 524*	
ENSPTRP00000011856 	833	Brt/Sky(ptk12)	 ig, 57 119 1.5e-12* fn3, 168 253 1.7e-08* fn3, 265 349 5.9e-06* Pkinase, 461 729 9.9e-143*	372 394*	
ENSPTRP00000025788 	1400	RON(ptk21)	 Sema, 58 507 6.0e-162* PSI, 526 568 4.5e-11* TIG, 569 670 6.4e-19* TIG, 684 766 1.4e-18* TIG, 770 859 2.1e-12* Pkinase, 1082 1341 2.1e-133*	960 982*	
ENSPTRP00000025789 	1351	RON(ptk21)	 Sema, 58 507 6.0e-162* PSI, 526 568 4.5e-11* TIG, 569 670 6.4e-19* TIG, 684 766 1.4e-18* TIG, 770 859 2.1e-12* Pkinase, 1033 1292 2.1e-133*	911 933*	
ENSPTRP00000033588 	1408	ptk21(ptk21)	 Sema, 55 500 4.3e-174* PSI, 519 562 2.2e-13* TIG, 563 654 2.8e-21* TIG, 657 738 1.7e-21* TIG, 762 853 9.3e-05* Pkinase, 1096 1355 7.2e-147*	951 973*	
ENSPTRP00000026572 	574	Nyk/RYK(ptk23)	 WIF, 65 159 7.6e-40* Pkinase, 297 563 2.1e-117*	193 215*	
ENSPTRP00000009773 	998	bPDGFR(ptk14)	 ig, 265 332 8.1e-08* Pkinase, 610 948 1.8e-151*	542 564*674 696*	
ENSPTRP00000029750 	1068	ckit(ptk14)	 ig, 47 102 3.7e-05* ig, 228 293 2.0e-06* Pkinase, 562 920 5.6e-154*	496 518*	
ENSPTRP00000027635 	1049	bPDGFR(ptk14)	 ig, 42 102 8.7e-03* ig, 228 292 2.7e-05* Pkinase, 553 910 7.6e-157*	487 509*553 575*	
ENSPTRP00000027637 	976	ckit(ptk14)	 ig, 226 292 4.0e-10* Pkinase, 589 924 2.2e-153*	521 543*653 675*	
ENSPTRP00000009777 	1338	Flt1(ptk14)	 ig, 245 313 1.6e-06* I-set, 332 426 2.0e-04* ig, 570 638 1.5e-04* I-set, 661 748 6.5e-23* Pkinase, 827 1154 2.1e-155*	
ENSPTRP00000027639 	1356	Flk1(ptk14)	 V-set, 224 325 2.0e-08* I-set, 330 417 3.5e-11* I-set, 667 754 4.1e-20* Pkinase, 834 1160 4.7e-154*	734 756*763 785*	
ENSPTRP00000045284 	1294	Flt4(ptk14)	 ig, 245 312 8.0e-07* I-set, 678 765 7.0e-12* Pkinase, 845 1157 6.6e-103*	776 798*	
ENSPTRP00000005306 	819	Bek(ptk15)	 ig, 55 109 9.1e-04* ig, 172 233 1.8e-11* I-set, 256 357 4.5e-05* Pkinase, 482 758 3.2e-159*	376 398*	
ENSPTRP00000005308 	822	FGFR-4(ptk15)	 ig, 55 109 9.1e-04* ig, 172 233 1.8e-11* I-set, 256 357 4.5e-05* Pkinase, 482 758 3.2e-159*	376 398*	
ENSPTRP00000005303 	707	FGFR-4(ptk15)	 ig, 57 118 1.8e-11* I-set, 141 242 4.5e-05* Pkinase, 367 643 3.2e-159*	261 283*	
ENSPTRP00000005302 	705	FGFR-4(ptk15)	 ig, 55 109 9.1e-04* ig, 172 233 1.8e-11* Pkinase, 365 641 3.2e-159*	
ENSPTRP00000005305 	709	FGFR-4(ptk15)	 ig, 55 109 9.1e-04* ig, 172 233 1.8e-11* Pkinase, 369 645 3.2e-159*	263 285*	
ENSPTRP00000043214 	823	Flg(ptk15)	 ig, 48 103 3.6e-05* I-set, 160 248 4.8e-10* I-set, 256 359 6.0e-08* Pkinase, 479 755 1.7e-160*	376 398*	
ENSPTRP00000027268 	808	Bek(ptk15)	 ig, 54 111 4.3e-05* I-set, 157 245 2.5e-10* I-set, 253 355 2.2e-04* Pkinase, 474 750 2.7e-160*	375 397*539 561*	
ENSPTRP00000027269 	694	Bek(ptk15)	 ig, 54 111 4.3e-05* I-set, 157 245 2.5e-10* Pkinase, 360 636 2.7e-160*	
ENSPTRP00000027267 	870	ptk3(ptk3)	 ig, 133 190 4.3e-05* I-set, 236 324 2.5e-10* ig, 347 419 1.4e-06* Pkinase, 551 815 4.8e-57*	451 473*616 638*	
ENSPTRP00000030013 	729	ptk9(ptk9)	 I-set, 153 241 2.6e-11* ig, 264 335 7.4e-06* Pkinase, 467 670 4.2e-65*	
ENSPTRP00000030012 	689	ptk9(ptk9)	 I-set, 153 241 2.6e-11* ig, 264 335 7.4e-06* Pkinase, 427 630 4.2e-65*	
ENSPTRP00000040099 	959	ptk9(ptk9)	 I-set, 269 357 2.6e-11* ig, 380 451 7.4e-06* Pkinase, 697 900 4.2e-65*	12 34*501 523*600 622*	
ENSPTRP00000004184 	1028	sp|P35546|RET_MOUSE(PTK8)	 Cadherin, 147 238 1.9e-08* Pkinase, 643 919 1.9e-127*	
ENSPTRP00000035632 	1109	Tek(ptk13)	 EGF_2, 224 251 1.3e-04* EGF_2, 315 340 5.1e-04* fn3, 444 529 2.3e-10* fn3, 543 626 4.4e-14* fn3, 639 724 5.4e-18* Pkinase, 824 1077 2.3e-115*	748 770*	
ENSPTRP00000035635 	1066	Tek(ptk13)	 EGF_2, 224 251 1.3e-04* fn3, 401 486 2.3e-10* fn3, 500 583 4.4e-14* fn3, 596 681 5.4e-18* Pkinase, 781 1034 2.3e-115*	705 727*	
ENSPTRP00000001085 	1138	Tek(ptk13)	 ig, 139 197 9.1e-03* Laminin_EGF, 231 272 3.2e-03* EGF_2, 319 344 1.6e-06* fn3, 446 533 7.3e-12* fn3, 546 632 1.8e-10* fn3, 644 729 4.2e-20* Pkinase, 839 1107 1.8e-134*	764 786*	
ENSPTRP00000049016 	1122	ptk7(ptk7)	 Pkinase, 524 801 1.6e-54* Pkinase, 832 1104 3.6e-123*	
ENSPTRP00000001425 	1156	JAK1(ptk7)	 SH2, 441 526 1.2e-03* Pkinase, 583 847 3.4e-77* Pkinase, 877 1151 2.0e-130*	
ENSPTRP00000048901 	1130	JAK2(ptk7)	 SH2, 377 457 4.0e-03* Pkinase, 521 777 1.7e-63* Pkinase, 822 1080 2.3e-94*	
ENSPTRP00000035490 	1132	JAK2(ptk7)	 SH2, 401 481 1.0e-04* Pkinase, 545 805 2.0e-80* Pkinase, 849 1123 5.9e-128*	
ENSPTRP00000034416 	1009	sp|Q14289|FAK2_HUMAN(PTK9)	 FERM_M, 143 265 3.9e-08* Pkinase, 425 679 1.3e-139* Focal_AT, 870 1008 1.1e-107*	
ENSPTRP00000034417 	967	sp|Q14289|FAK2_HUMAN(PTK9)	 FERM_M, 143 265 3.9e-08* Pkinase, 425 679 1.3e-139* Focal_AT, 828 966 1.1e-107*	
ENSPTRP00000041743 	878	sp|Q14289|FAK2_HUMAN(PTK9)	 Pkinase, 240 523 1.1e-133* Focal_AT, 740 878 8.0e-106*	
ENSPTRP00000035268 	554	ptk9(ptk9)	 Pkinase, 240 523 1.1e-133*	
ENSPTRP00000035264 	1052	sp|Q14289|FAK2_HUMAN(PTK9)	 FERM_M, 139 258 6.2e-03* Pkinase, 422 676 1.3e-146* Focal_AT, 914 1052 8.0e-106*	
ENSPTRP00000033400 	892	HEK2(ptk11)	 Ephrin_lbd, 17 102 6.1e-14* GCC2_GCC3, 163 207 7.1e-04* fn3, 229 319 1.2e-09* fn3, 339 424 1.7e-18* Pkinase, 520 779 5.5e-141* SAM_1, 810 874 2.6e-29*	446 468*	
ENSPTRP00000052704 	1034	HEK2(ptk11)	 Ephrin_lbd, 1 176 5.8e-139* GCC2_GCC3, 238 282 1.1e-04* fn3, 304 400 2.3e-16* fn3, 415 499 2.8e-22* Pkinase, 600 859 1.1e-149* SAM_1, 890 954 5.5e-31*	522 544*	
ENSPTRP00000042082 	947	HEK2(ptk11)	 Ephrin_lbd, 19 196 3.8e-140* fn3, 323 414 9.4e-10* fn3, 434 518 2.3e-20* Pkinase, 619 841 5.5e-116* SAM_1, 872 936 1.7e-28*	541 563*	
ENSPTRP00000027008 	997	HEK2(ptk11)	 Ephrin_lbd, 38 211 1.8e-136* GCC2_GCC3, 273 319 3.8e-03* fn3, 339 434 5.6e-16* fn3, 452 534 3.4e-20* Pkinase, 632 891 4.7e-144* SAM_1, 922 986 3.6e-33*	559 581*	
ENSPTRP00000027683 	1037	Ehk-1(ptk11)	 Ephrin_lbd, 60 233 3.3e-140* fn3, 358 454 2.3e-12* fn3, 469 552 1.1e-17* Pkinase, 675 932 8.1e-149* SAM_1, 963 1027 1.1e-30*	
ENSPTRP00000027681 	1015	Ehk-1(ptk11)	 Ephrin_lbd, 60 233 3.3e-140* fn3, 358 454 2.3e-12* fn3, 469 552 1.1e-17* Pkinase, 653 910 8.1e-149* SAM_1, 941 1005 1.1e-30*	574 596*	
ENSPTRP00000026114 	983	HEK(ptk11)	 Ephrin_lbd, 29 202 4.7e-144* fn3, 326 419 3.3e-13* fn3, 437 521 2.1e-19* Pkinase, 621 878 9.2e-152* SAM_2, 908 975 1.2e-18*	542 564*	
ENSPTRP00000022158 	986	Ehk-1(ptk11)	 Ephrin_lbd, 30 204 5.1e-146* fn3, 329 420 7.3e-13* fn3, 441 525 6.6e-20* Pkinase, 621 878 1.2e-145* SAM_2, 908 975 9.5e-25*	548 570*	
ENSPTRP00000026129 	1036	Ehk-2(ptk11)	 Ephrin_lbd, 34 207 2.3e-140* GCC2_GCC3, 268 312 4.9e-03* fn3, 332 425 8.6e-07* fn3, 440 527 9.1e-16* Pkinase, 631 930 8.3e-146* SAM_1, 959 1023 4.2e-28*	549 571*	
ENSPTRP00000031479 	997	Ehk-1(ptk11)	 Ephrin_lbd, 32 205 1.1e-141* GCC2_GCC3, 268 312 3.0e-05* fn3, 332 427 8.3e-10* fn3, 443 527 5.1e-20* Pkinase, 632 889 5.0e-149* SAM_1, 920 984 6.8e-32*	555 577*	
ENSPTRP00000000400 	930	ptk11(ptk11)	 Ephrin_lbd, 28 201 1.4e-126* fn3, 329 424 1.6e-13* fn3, 436 506 6.0e-10* Pkinase, 600 825 1.7e-116* SAM_1, 856 920 1.1e-30*	523 545*	
ENSPTRP00000033933 	976	EPH(ptk11)	 Ephrin_lbd, 27 204 2.1e-116* fn3, 333 431 1.2e-14* fn3, 448 528 4.3e-04* Pkinase, 624 880 1.8e-144* SAM_1, 911 975 8.5e-32*	548 570*	
ENSPTRP00000014805 	666	ptk8(ptk8)	 Pkinase, 116 377 7.1e-116*	
ENSPTRP00000015508 	1247	ErbB2(ptk10)	 Recep_L_domain, 52 173 2.2e-43* Furin-like, 189 343 4.1e-94* Recep_L_domain, 366 486 7.2e-31* Pkinase, 720 976 1.4e-133*	653 675*772 794*	
ENSPTRP00000032807 	1210	ptk10(ptk10)	 Recep_L_domain, 57 168 2.9e-42* Furin-like, 184 338 2.4e-101* Recep_L_domain, 361 481 1.8e-43* Pkinase, 712 968 1.4e-117*	646 668*773 795*	
ENSPTRP00000022026 	1308	ErbB4(ptk10)	 Recep_L_domain, 55 167 1.2e-46* Furin-like, 183 335 1.2e-78* Recep_L_domain, 358 478 2.7e-39* Pkinase, 718 974 2.2e-142*	653 675*780 799*	
ENSPTRP00000036060 	544	ptk6(ptk6)	 SH2, 15 72 7.0e-04* SH2, 77 153 5.9e-32* Pkinase, 279 535 1.9e-127*	
ENSPTRP00000036059 	521	ptk6(ptk6)	 SH2, 15 72 7.0e-04* SH2, 77 153 5.9e-32* Pkinase, 256 512 1.9e-127*	
ENSPTRP00000021008 	628	ZAP70(ptk6)	 SH2, 10 87 2.0e-28* SH2, 163 239 1.1e-29* Pkinase, 338 602 2.9e-127*	
ENSPTRP00000016601 	742	ptk18(ptk18)	 Pkinase, 76 315 8.1e-40*	
ENSPTRP00000016600 	1193	ptk18(ptk18)	 Pkinase, 28 267 8.1e-40*	
ENSPTRP00000047818 	1503	Dror(ptk19)	 Pkinase, 137 407 1.1e-47*	12 34*44 66*145 167*	
ENSPTRP00000031080 	1070	sp|Q13308|PTK7_HUMAN(PTK18)	 ig, 46 103 9.8e-08* I-set, 128 217 5.2e-13* ig, 239 303 2.2e-04* ig, 336 393 3.4e-06* I-set, 412 498 7.2e-17* I-set, 502 588 2.2e-14* I-set, 592 681 4.0e-11* Pkinase, 796 1061 2.9e-116*	13 35*704 726*	
ENSPTRP00000031079 	1030	sp|Q13308|PTK7_HUMAN(PTK18)	 ig, 46 103 9.8e-08* I-set, 128 217 5.2e-13* ig, 239 303 2.2e-04* ig, 336 393 3.4e-06* I-set, 412 498 7.2e-17* I-set, 552 641 4.0e-11* Pkinase, 756 1021 2.9e-116*	13 35*664 686*	
ENSPTRP00000031081 	940	sp|Q13308|PTK7_HUMAN(PTK18)	 ig, 46 103 9.8e-08* I-set, 128 217 5.2e-13* ig, 239 303 2.2e-04* ig, 336 393 3.4e-06* I-set, 462 551 4.0e-11* Pkinase, 666 931 2.9e-116*	13 35*574 596*	
ENSPTRP00000039569 	1015	sp|Q13308|PTK7_HUMAN(PTK18)	 ig, 46 103 9.8e-08* I-set, 128 217 5.2e-13* ig, 239 303 2.2e-04* ig, 336 393 3.4e-06* I-set, 412 498 7.2e-17* I-set, 502 588 2.2e-14* Pkinase, 741 1006 2.9e-116*	13 35*649 671*	
ENSPTRP00000031083 	396	ptk18(ptk18)	 ig, 12 69 3.4e-06* Pkinase, 122 387 1.4e-108*	
ENSPTRP00000008008 	387	BFR2_HUMAN/367-643(kinase)	 Pkinase, 99 345 2.7e-48*	26 48*	
ENSPTRP00000023967 	786	pelle(plantrk)	 Pkinase, 22 282 7.5e-43* Ank, 439 471 1.3e-06* Ank, 472 504 1.8e-06* Ank, 505 537 4.4e-07* Ank, 538 570 1.7e-08* Ank, 571 604 2.6e-04* Ank, 605 637 4.1e-09* Ank, 638 670 5.2e-05* Ank, 671 703 4.3e-09* Ank, 704 734 2.9e-04* Ank, 736 768 1.0e-06*	
ENSPTRP00000023968 	834	pelle(plantrk)	 Pkinase, 22 293 6.3e-44* Ank, 487 519 1.3e-06* Ank, 520 552 1.8e-06* Ank, 553 585 4.4e-07* Ank, 586 618 1.7e-08* Ank, 619 652 2.6e-04* Ank, 653 685 4.1e-09* Ank, 686 718 5.2e-05* Ank, 719 751 4.3e-09* Ank, 752 782 2.9e-04* Ank, 784 816 1.0e-06*	
ENSPTRP00000047639 	763	PRO25(plantrk)	 Pkinase, 26 281 1.5e-33* Ank, 361 393 9.3e-04* Ank, 394 426 1.1e-03* Ank, 427 458 2.3e-03* Ank, 459 491 2.1e-08* Ank, 492 523 4.2e-04* Ank, 524 556 1.4e-06* Ank, 557 589 8.3e-06* Ank, 590 622 1.3e-06* Ank, 623 655 2.0e-09* Ank, 656 688 2.0e-05* Ank, 689 721 6.8e-07*	
ENSPTRP00000034905 	539	Ctr1(raf)	 Pkinase, 18 290 9.7e-42* CARD, 436 523 6.1e-24*	
ENSPTRP00000010556 	518	Ctr1(raf)	 Pkinase, 21 282 2.5e-48*	
ENSPTRP00000047059 	426	Ctr1(raf)	 Pkinase, 21 282 2.5e-48*	
ENSPTRP00000030180 	671	DtSpk-1(ptk2)	 Pkinase, 17 285 3.7e-51* Death, 584 669 1.3e-22*	
ENSPTRP00000024574 	638	FER(ptk4)	 LIM, 12 68 1.4e-20* LIM, 72 129 1.6e-08* PDZ, 152 236 2.9e-20* Pkinase, 331 601 4.4e-46*	
ENSPTRP00000049722 	684	ELK(ptk11)	 LIM, 6 47 8.4e-06* LIM, 51 108 1.6e-08* PDZ, 131 215 2.9e-20* Pkinase, 310 613 6.8e-42*	
ENSPTRP00000032971 	640	1SRK(ptk1)	 LIM, 25 80 1.0e-22* LIM, 84 142 1.5e-21* PDZ, 165 255 5.3e-19* Pkinase, 339 597 2.5e-39*	
ENSPTRP00000001162 	571	M3K9_HUMAN/3-262(kinase)	 Pkinase, 59 309 4.4e-43*	
ENSPTRP00000042882 	542	Ctr1(raf)	 Pkinase, 59 335 4.1e-34*	
ENSPTRP00000035769 	626	KYK2_DICDI/108-364(kinase)	 Pkinase, 57 311 1.6e-45*	
ENSPTRP00000011015 	1104	PTK1(mlk)	 SH3_1, 56 115 3.1e-15* Pkinase, 130 389 1.6e-94*	
ENSPTRP00000006703 	847	PTK1(mlk)	 SH3_1, 44 103 3.9e-16* Pkinase, 117 376 4.2e-81*	
ENSPTRP00000003583 	1035	PTK1(mlk)	 SH3_1, 41 99 1.7e-08* Pkinase, 123 397 2.5e-78*	
ENSPTRP00000018851 	954	PTK1(mlk)	 SH3_1, 19 79 1.3e-15* Pkinase, 98 357 8.3e-77*	
ENSPTRP00000008543 	850	M3K9_HUMAN/3-262(kinase)	 Pkinase, 125 364 5.6e-71*	
ENSPTRP00000027014 	966	KYK2_DICDI/108-364(kinase)	 Pkinase, 168 407 3.3e-73*	
ENSPTRP00000021606 	800	M3K9_HUMAN/3-262(kinase)	 Pkinase, 16 259 1.2e-66* SAM_2, 335 410 3.7e-07*	
ENSPTRP00000049030 	453	M3K9_HUMAN/3-262(kinase)	 Pkinase, 16 259 1.2e-66*	
ENSPTRP00000031477 	579	KYK1_DICDI/1289-1559(kinase)	 Pkinase, 36 284 5.7e-65*	
ENSPTRP00000031478 	518	KYK1_DICDI/1289-1559(kinase)	 Pkinase, 36 284 5.7e-65*	
ENSPTRP00000001494 	936	KYK1_DICDI/1289-1559(kinase)	 Ank, 201 233 2.8e-07* Ank, 234 266 5.6e-09* Ank, 267 299 1.2e-07* Ank, 300 334 2.7e-06* Ank, 370 403 1.5e-03* Ank, 440 472 2.4e-07* Pkinase, 564 820 3.7e-62*	
ENSPTRP00000004727 	532	ALK-3(tgfb)	 Activin_recp, 59 138 6.9e-23* TGF_beta_GS, 204 232 2.3e-13* Pkinase, 234 521 2.5e-61*	153 175*	
ENSPTRP00000027988 	501	ALK-6(tgfb)	 Activin_recp, 30 110 1.7e-27* TGF_beta_GS, 173 201 2.2e-13* Pkinase, 203 490 4.6e-59*	126 148*	
ENSPTRP00000049145 	426	ALK-5(tgfb)	 Activin_recp, 34 114 2.3e-25* Pkinase, 128 415 7.3e-61*	12 34*	
ENSPTRP00000036200 	503	ALK-5(tgfb)	 Activin_recp, 34 114 2.3e-25* TGF_beta_GS, 175 203 1.2e-15* Pkinase, 205 492 7.3e-61*	12 34*126 148*	
ENSPTRP00000008474 	505	ALK-4(tgfb)	 Activin_recp, 32 109 7.9e-29* TGF_beta_GS, 177 205 4.9e-14* Pkinase, 207 494 2.5e-54*	127 149*	
ENSPTRP00000021454 	336	ALK-5(tgfb)	 Activin_recp, 26 100 1.1e-23*	
ENSPTRP00000021455 	493	ALK-5(tgfb)	 Activin_recp, 26 100 1.1e-23* TGF_beta_GS, 165 193 1.8e-14* Pkinase, 195 482 3.4e-44*	114 136*	
ENSPTRP00000021456 	413	ALK-5(tgfb)	 Activin_recp, 26 100 1.1e-23* Pkinase, 115 402 3.4e-44*	
ENSPTRP00000041638 	503	TSR-1(tgfb)	 Activin_recp, 32 103 6.7e-12* TGF_beta_GS, 172 200 2.0e-13* Pkinase, 202 489 6.8e-60*	119 141*	
ENSPTRP00000021457 	509	TskL7(tgfb)	 Activin_recp, 33 107 1.4e-23* TGF_beta_GS, 178 206 3.6e-15* Pkinase, 208 495 1.9e-77*	124 146*	
ENSPTRP00000048858 	1040	C14(tgfb)	 Activin_recp, 32 133 3.9e-27* Pkinase, 205 503 8.1e-40*	154 176*	
ENSPTRP00000008537 	573	C14(tgfb)	 Pkinase, 203 505 4.4e-27*	148 170*	
ENSPTRP00000038918 	592	tgfb(tgfb)	 ecTbetaR2, 72 190 1.6e-93* Pkinase, 269 563 7.7e-52*	192 214*	
ENSPTRP00000025375 	567	tgfb(tgfb)	 ecTbetaR2, 47 165 1.6e-93* Pkinase, 244 538 7.7e-52*	167 189*	
ENSPTRP00000025456 	512	ActRIIB(tgfb)	 Activin_recp, 27 117 2.3e-20* Pkinase, 190 478 4.0e-61*	138 160*	
ENSPTRP00000021405 	512	ActRIIA(tgfb)	 Activin_recp, 28 118 4.5e-08* Pkinase, 192 478 9.9e-73*	139 161*	
ENSPTRP00000052965 	846	Araf(raf)	 Pkinase, 560 824 2.4e-39*	
ENSPTRP00000015183 	892	Araf(raf)	 Pkinase, 582 846 2.4e-39*	
ENSPTRP00000009350 	826	Braf(raf)	 C1_1, 292 339 2.6e-05* Pkinase, 543 805 1.1e-39*	
ENSPTRP00000037507 	393	Araf(raf)	 RBD, 19 85 1.9e-25* Pkinase, 170 393 1.9e-46*	
ENSPTRP00000033866 	766	raf(raf)	 RBD, 155 227 8.3e-39* C1_1, 235 283 4.0e-17* Pkinase, 457 714 1.8e-83*	
ENSPTRP00000025252 	648	raf(raf)	 RBD, 56 131 2.0e-40* C1_1, 139 187 4.0e-18* Pkinase, 349 606 1.7e-84*	
ENSPTRP00000038647 	636	SR2(plantrk)	 Pkinase, 166 454 4.5e-25*	
ENSPTRP00000038648 	557	SR2(plantrk)	 Pkinase, 166 476 3.3e-20*	
ENSPTRP00000008285 	460	Pto(plantrk)	 Pkinase, 186 454 6.7e-48*	
ENSPTRP00000015697 	1243	Ctr1(raf)	 Pkinase, 174 432 1.1e-59*	
ENSPTRP00000007716 	2136	Ctr1(raf)	 Pkinase, 221 479 6.5e-62*	
ENSPTRP00000037688 	1799	Ctr1(raf)	 Pkinase, 147 405 3.1e-63*	
ENSPTRP00000036100 	1971	KMIL_AVIMH/82-339(kinase)	 Pkinase, 41 299 5.1e-61*	
ENSPTRP00000045346 	2044	KMIL_AVIMH/82-339(kinase)	 Pkinase, 41 299 5.1e-61*	
ENSPTRP00000036099 	1954	KMIL_AVIMH/82-339(kinase)	 Pkinase, 41 299 5.1e-61*	
ENSPTRP00000031399 	857	TTK_HUMAN/509-775(kinase)	 Pkinase, 525 791 6.2e-93*	
ENSPTRP00000008254 	2527	M3K9_HUMAN/3-262(kinase)	 Miro, 1336 1455 9.3e-35* Pkinase, 1879 2132 1.7e-35*	
ENSPTRP00000003173 	930	IIApl(agc2)	 Pkinase, 653 910 1.4e-36*	
ENSPTRP00000013476 	974	AKIN10(camk2)	 Pkinase, 568 829 1.6e-41* Ribonuc_2-5A, 834 960 3.1e-70*	
ENSPTRP00000034701 	346	KMOS_CERAE/60-338(kinase)	 Pkinase, 60 338 9.6e-71*	
ENSPTRP00000007718 	2382	Ctr1(raf)	 Pkinase, 221 479 6.5e-62*	
ENSPTRP00000037254 	674	2RSKN(agc6)	 Pkinase, 45 268 1.3e-59* Pkinase_C, 288 332 7.2e-10* Pkinase, 363 620 4.6e-110*	
ENSPTRP00000054908 	693	2RSKN(agc6)	 Pkinase, 28 287 5.5e-70* Pkinase_C, 307 351 7.2e-10* Pkinase, 382 639 4.6e-110*	
ENSPTRP00000000500 	581	kinase(kinase)	 Pkinase, 156 512 1.2e-15*	
ENSPTRP00000000553 	930	kinase(kinase)	 Ephrin_lbd, 31 204 3.3e-135* fn3, 329 422 1.6e-08* fn3, 435 522 1.7e-18* Pkinase, 633 817 4.0e-56* SAM_1, 856 917 1.1e-25*	539 561*	
ENSPTRP00000000659 	341	kinase(kinase)	 Pkinase, 8 329 1.1e-48*	
ENSPTRP00000000678 	228	kinase(kinase)	 Pkinase_C, 61 105 1.0e-12*	
ENSPTRP00000000948 	406	kinase(kinase)	 Pkinase, 40 299 8.5e-11*	
ENSPTRP00000001636 	574	kinase(kinase)	 Pkinase, 58 569 1.5e-38*	
ENSPTRP00000002674 	419	kinase(kinase)	 Pkinase, 23 304 4.7e-34* RRM_1, 345 401 3.7e-03*	
ENSPTRP00000003174 	885	kinase(kinase)	 Pkinase, 653 865 9.1e-16*	
ENSPTRP00000004266 	218	kinase(kinase)	 Pkinase, 26 217 1.3e-24*	
ENSPTRP00000005578 	513	kinase(kinase)	 Pkinase, 19 163 0.00022*	
ENSPTRP00000005579 	446	kinase(kinase)	 Pkinase, 19 163 0.00022*	
ENSPTRP00000011804 	1050	kinase(kinase)	 Mad3_BUB1_I, 55 179 1.4e-72*	
ENSPTRP00000012830 	1942	kinase(kinase)	 Miro, 640 760 1.8e-06* Pkinase, 1234 1563 1.2e-12*	
ENSPTRP00000013537 	373	kinase(kinase)	 Pkinase, 53 262 1.8e-19*	
ENSPTRP00000014475 	214	kinase(kinase)	 Pkinase, 6 208 6.5e-09*	
ENSPTRP00000014642 	798	kinase(kinase)	 Pkinase, 484 774 2.6e-03*	
ENSPTRP00000015235 	274	kinase(kinase)	 Pkinase, 107 274 3.0e-09*	
ENSPTRP00000015698 	661	kinase(kinase)	 Pkinase, 1 203 1.3e-13*	
ENSPTRP00000019777 	651	kinase(kinase)	 Pkinase, 1 188 1.0e-13*	
ENSPTRP00000020366 	294	kinase(kinase)			
ENSPTRP00000020635 	896	kinase(kinase)	 Pkinase, 77 341 1.2e-36*	
ENSPTRP00000021062 	1023	kinase(kinase)	 Pkinase, 6 321 3.9e-13* CNH, 710 1001 6.2e-110*	
ENSPTRP00000021143 	1087	kinase(kinase)	 Mad3_BUB1_I, 4 126 9.5e-58* Pkinase, 787 1075 5.5e-06*	
ENSPTRP00000022552 	401	kinase(kinase)	 Pkinase, 69 394 3.3e-52*	
ENSPTRP00000023360 	253	kinase(kinase)	 Kdo, 17 225 1.5e-06*	
ENSPTRP00000025360 	402	kinase(kinase)	 Pkinase, 3 180 1.2e-14*	
ENSPTRP00000025362 	713	kinase(kinase)	 Pkinase, 520 713 2.2e-12*	
ENSPTRP00000025492 	541	kinase(kinase)	 Pkinase, 4 280 2.8e-54*	
ENSPTRP00000026130 	332	kinase(kinase)	 Pkinase, 115 328 1.7e-45*	33 55*	
ENSPTRP00000026520 	1358	kinase(kinase)	 Pkinase, 26 310 3.6e-08* HEAT, 412 448 4.5e-05* HEAT, 457 493 9.7e-03* WD40, 983 1021 4.5e-09* WD40, 1229 1269 3.5e-03* WD40, 1319 1358 1.5e-04*	
ENSPTRP00000029749 	901	kinase(kinase)	 V-set, 19 102 3.6e-03* ig, 217 280 5.4e-08* ig, 412 487 3.7e-05* Pkinase, 582 839 1.7e-75*	515 537*	
ENSPTRP00000033503 	654	kinase(kinase)	 Pkinase, 80 652 1.7e-36*	
ENSPTRP00000034429 	322	kinase(kinase)	 Pkinase, 32 318 1.9e-29*	
ENSPTRP00000034734 	248	kinase(kinase)	 Pkinase, 53 248 9.4e-29*	
ENSPTRP00000038616 	497	kinase(kinase)	 Pkinase, 46 495 9.7e-38*	
ENSPTRP00000041696 	549	kinase(kinase)	 MIT, 49 117 5.4e-23* Pkinase, 116 516 5.4e-10*	
ENSPTRP00000044093 	580	kinase(kinase)	 MIT, 49 117 5.4e-23* Pkinase, 153 547 4.2e-09*	
ENSPTRP00000045239 	543	kinase(kinase)	 Pkinase, 29 209 0.00025*	
ENSPTRP00000045362 	309	kinase(kinase)	 Pkinase, 20 290 1.0e-38*	
ENSPTRP00000045837 	687	kinase(kinase)	 Pkinase, 80 685 4.9e-37*	
ENSPTRP00000046588 	530	kinase(kinase)	 Pkinase, 46 528 1.1e-38*	
ENSPTRP00000050289 	478	kinase(kinase)	 Pkinase, 203 410 3.3e-11*	148 170*	
