# Supplementary material for: Comparative kinomics of human and chimpanzee reveal unique kinship and functional diversity generated by new domain combinations
Source: BMC Genomics. 2008 Dec 23;9:625. doi: 10.1186/1471-2164-9-625 (PMC2651890; doi:10.1186/1471-2164-9-625)
Supplement: Additional file 2 — Sequence alignment between some of the closest chimp and human kinase pairs with difference in domain architecture. [file 1471-2164-9-625-s2.pdf]

Additional file 2: Sequence alignment between some of the closest chimp and human kinase pairs with difference in domain architecture.

A) Alignment showing Human and Chimp protein kinase C. The C1 domain which is present in the human PKC and not in Chimp PKC is highlighted in red. Moreover, human kinase has an insertion in the kinase catalytic domain which is highlighted in gray colour.

ENSPTRP00000000076 (Chimp protein) and ENSP00000367830 (Human protein)

```
Chimp      MPSRTGPKMEGSGGRVRLKAHYGGDIFITSVDAATTFEELCEEVRDMCRLHQQHPLTLKW
Human      MPSRTGPKMEGSGGRVRLKAHYGGDIFITSVDAATTFEELCEEVRDMCRLHQQHPLTLKW

Chimp      VDSEGDPCTVSSQMELEEAFRLARQCRDEGLIIHVFPSTPEQPGLPCPGEDKSIYRRGAR
Human      VDSEGDPCTVSSQMELEEAFRLARQCRDEGLIIHVFPSTPEQPGLPCPGEDKSIYRRGAR

Chimp      RWRKLYCANGHLFQAKRFNR----------------------------------------
Human      RWRKLYRANGHLFQAKRFNRRAYCGQCSERIWGLARQGYRCINCKLLVHKRCHGLVPLTC

Chimp      ----DSVMPSQEPPVDDKNEDADLPSEETDGIAYISSSRKHDSIKDDSEDLKPVIDGMDG
Human      RKHMDSVMPSQEPPVDDKNEDADLPSEETDGIAYISSSRKHDSIKDDSEDLKPVIDGMDG

Chimp      IKISQGLGLQDFDLIRVIGRGSYAKVLLVRLKKNDQIYAMKVVKKELVHDDE--------
Human      IKISQGLGLQDFDLIRVIGRGSYAKVLLVRLKKNDQIYAMKVVKKELVHDDEDIDWVQTE

Chimp      ---------------------TTSRLFLVIEYVNGGDLMFHMQRQRKLPEEHARFYAAEI
Human      KHVFEQASSNPFLVGLHSCFQTTSRLFLVIEYVNGGDLMFHMQRQRKLPEEHARFYAAEI

Chimp      CIALNFLHERGIIYRDLKLDNVLLDADGHIKLTDYGMCKEGLGPGDTTSTFCGTPNYIAP
Human      CIALNFLHERGIIYRDLKLDNVLLDADGHIKLTDYGMCKEGLGPGDTTSTFCGTPNYIAP

Chimp      EILRGEEYGFSVDWWALGVLMFEMMAGRSPFDIITDNPDMNTEDYLFQVILEKPIRIPRF
Human      EILRGEEYGFSVDWWALGVLMFEMMAGRSPFDIITDNPDMNTEDYLFQVILEKPIRIPRF

Chimp      LSVKASHVLKGFLNKDPKERLGCRPQTGFSDIKSHAFFRSIDWDLLEKKQALPPFQPQIT
Human      LSVKASHVLKGFLNKDPKERLGCRPQTGFSDIKSHAFFRSIDWDLLEKKQALPPFQPQIT

Chimp      DDYGLDNFDTQFTSEPVQLTPDDEDAIKRIDQSEFEGFEYINPLLLSTEESV
Human      DDYGLDNFDTQFTSEPVQLTPDDEDAIKRIDQSEFEGFEYINPLLLSTEESV
```

B) Alignment showing Chimp protein kinase and Human kinase which belong to AGC group. The putative transmembrane region which is present in the chimp protein kinase is highlighted in red. The human kinase has extra region which is highlighted in gray colour.

ENSPTRP00000019171 (Chimp proteins) and ENSP00000291270 (Human protein)

```
Chimp       MSAEVRLRRLQQLVLDPGFLGLEPLLDLLLGVHQELGASELAQDKYVADFLQWAEPIVVR
Human       MSAEVRLRRLQQLVLDPGFLGLEPLLDLLLGVHQELGASELAQDKYVADFLQWAEPIVVR

Chimp       LKEVRLQRDDFEILKVIGRGAFSEVAVVKMKQTGQVYAMKIMNKWDMLKRGEVSCFREER
Human       LKEVRLQRDDFEILKVIGRGAFSEVAVVKMKQTGQVYAMKIMNKWDMLKRGEVSCFREER

Chimp       DVLVNGDRRWITQLHFAFQDENYLYLVMEYYVGGDLLTLLSKFGERIPAEMARFYLAEIV
Human       DVLVNGDRRWITQLHFAFQDENYLYLVMEYYVGGDLLTLLSKFGERIPAEMARFYLAEIV

Chimp       MAIDSVHRLGYVHRDIKPDNILLDRCGHIRLADFGSCLKLRADGTVRSLVAVGTPDYLSP
Human       MAIDSVHRLGYVHRDIKPDNILLDRCGHIRLADFGSCLKLRADGTVRSLVAVGTPDYLSP

Chimp       EILQAVGGGPGTGSYGPECDWWALGVFAYEMFYGQTPFYADSTAETYGKIVHYKEHLSLP
Human       EILQAVGGGPGTGSYGPECDWWALGVFAYEMFYGQTPFYADSTAETYGKIVHYKEHLSLP

Chimp       LVDEGVPEEARDFIQRLLCPPETRLGRGGAGDFRTHPFFFGLDWDGLRDSVPPFTPDFEG
Human       LVDEGVPEEARDFIQRLLCPPETRLGRGGAGDFRTHPFFFGLDWDGLRDSVPPFTPDFEG

Chimp       ATDTCNFDLVEDGLTAMVSGGGETLSDIREGAPLGVHLPFVGYSYSCMALRDSEVPGPTP
Human       ATDTCNFDLVEDGLTAMVSGGGETLSDIREGAPLGVHLPFVGYSYSCMALRDSEVPGPTP

Chimp       MELEAEQLLEPHVQAPSLEPSVSPQDETAEVAVPAAVPAAEAEAEVTLRELQEALEEEVL
Human       MELEAEQLLEPHVQAPSLEPSVSPQDETAEVAVPAAVPAAEAEAEVTLRELQEALEEEVL

Chimp       TRQSLSREMEAIRTDNQNFASQLREAEARNRDLEAHVRQLQERMELLQAEGATAVTGVPS
Human       TRQSLSREMEAIRTDNQNFASQLREAEARNRDLEAHVRQLQERMELLQAEGATAVTGVPS

Chimp       PRATDPPSH------------------------------VPWPGLSXALSLLLFAVVLSR
Human       PRATDPPSHLDGPPAVAVGQCPLVGPGPMHRRHLLLPARVPRPGLSEALSLLLFAVVLSR

Chimp       AAALGCLGLVAPAGXLXAVWRRPGAARAPX
Human       AAALGCIGLVAHAGQLTAVWRRPGAARAP-
```

C) Alignment showing Chimp CK1 and Human CK1. The POLO-BOX domain which is present in Chimp kinase is highlighted in red. Moreover, the Chimp kinase has insertion between kinase domain and POLO-BOX domain which is highlighted in gray.

ENSPTRP00000001150 (Chimp protein) and ENSP00000324464 (Human protein)

```
Chimp      --------------------------------------------------
Human      MELRVGNRYRLGRKIGSGSFGDIYLGTDIAAGEEVAIKLECVKTKHPQLH

Chimp      ----------------------------------------SLAHIWKARH
Human      IESKIYKMMQGGVGIPTIRWCGAEGDYNVMVMELLGPSLEDLFNFCSRKF

Chimp      TLLEPEVRYYLRQILSGLKYLHQRGILHRDLKLGNFFI---TENMELKVG
Human      SL--KTVLLLADQMISRIEYIHSKNFIHRDVKPDNFLMGLGKKGNLVYII

Chimp      DFGLAARLEP-------PEQRKKTICGTPNYVAPEVLLRQGHGPEADVWS
Human      DFGLAKKYRDARTHQHIPYRENKNLTGTARYASINTHLGIEQSRRDDLES


Chimp      LGCVMYTLLCGSPPFE---TADLKETYRCIKQVHYTLPASLSLPA-----
Human      LGYVLMYFNLGSLPWQGLKAATKRQKYERISEKKMSTPIEVLCKGYPSEF

Chimp      ---RQLLAAILRASPRDRPSIDQILRHDFFTKGYTPDRLPISSCVTVPDL
Human      ATYLNFCRSLRFDDKPDYSYLRQLFRNLFHRQGFSYDYVFDWNMLKFGAS

Chimp      TPPNPARSLFAKVTKSLFGRKKKKSKNHAQESDEVSGLVSGLMRTSVGHQ
Human      RAADDAER----------ERRDREERLRHSRNPATRGLPSTASGRLRGTQ

Chimp      DARPEAP-------AASGPAPVSLVE----------------------TA
Human      EVAPPTPLTPTSHTANTSPRPVSGMERERKVSMRLHRGAPVNISSSDLTG

Chimp      PEDSSPRGTLASSGDGFEEGLTVATVVESALCALRNCVAFMPPAEQNPAP
Human      RQDTSRMSTSQIPGRVASSGL--QSVVHR---------------------

Chimp      LAQPEPLVWVSKWVDYGGDLPSVEEVEVPAPPLLLQWVKTDQALLMLFSD
Human      --------------------------------------------------

Chimp      GTVQVNFYGDHTKLILSGWEPLLVTFVARNRSACTYLASHLRQLGCSPDL
Human      --------------------------------------------------

Chimp      RQRLRYALRLLRDRSPA
Human      -----------------
```

D) Alignment showing Chimp kinase and Human kinase which belongs to CAMK group and AGC group respectively. Human kinase has longer protein kinase domain and protein kinase C domain detected in the C-terminal at stringent e-value which is highlighted in gray and red colour respectively.

ENSPTRP00000011569 (Chimp protein) and ENSP00000270202 (Human protein)

```
Chimp       MSDVAIVKEGWLHKRGEYIKTWRPRYFLLKNDGTFIGYKERPQDVDQREAPLNNFSVAQC
Human       MSDVAIVKEGWLHKRGEYIKTWRPRYFLLKNDGTFIGYKERPQDVDQREAPLNNFSVAQC

Chimp       QLMKTERPRPNTFIIRCLQWTTVIERTFHVETPEEREEWTTAIQTVADGLKKQEEEEMDF
Human       QLMKTERPRPNTFIIRCLQWTTVIERTFHVETPEEREEWTTAIQTVADGLKKQEEEEMDF

Chimp       RSGSPSDNSGAEEMEVSLAKPKHRVTMNEFEYLKLLGKGTFGKVILVKEKATGRYYAMKI
Human       RSGSPSDNSGAEEMEVSLAKPKHRVTMNEFEYLKLLGKGTFGKVILVKEKATGRYYAMKI


Chimp       LKKEVIVAKDEVAHTLTENRVLQNSRHPFLTALKYSFQTHDRLCFVMEYANGGELFFHLS
Human       LKKEVIVAKDEVAHTLTENRVLQNSRHPFLTALKYSFQTHDRLCFVMEYANGGELFFHLS

Chimp       RERVFSEDRARFYGAEIVSALDYLHSEKNVVYRDLKLENLMLDKDGHIKITDFGLCKEGI
Human       RERVFSEDRARFYGAEIVSALDYLHSEKNVVYRDLKLENLMLDKDGHIKITDFGLCKEGI

Chimp       KDGATMKTFCGTSEYLAP------------------------------------------
Human       KDGATMKTFCGTPEYLAPEVLEDNDYGRAVDWWGLGVVMYEMMCGRLPFYNQDHEKLFEL

Chimp       -----------------------------------------------------------R
Human       ILMEEIRFPRTLGPEAKSLLSGLLKKDPKQRLGGGSEDAKEIMQHRFFAGIVWQHVYEKK

Chimp       LSPPFKPQVTSETDTRYFDEEFTAQMITITPPDQDDSMECVDSERRPHFPQFSYSASGTA
Human       LSPPFKPQVTSETDTRYFDEEFTAQMITITPPDQDDSMECVDSERRPHFPQFSYSASGTA
```
