# Supplementary material for: Comparative kinomics of human and chimpanzee reveal unique kinship and functional diversity generated by new domain combinations
Source: BMC Genomics. 2008 Dec 23;9:625. doi: 10.1186/1471-2164-9-625 (PMC2651890; doi:10.1186/1471-2164-9-625)
Supplement: Additional file 3 — List of chimpanzee protein kinases and their putative orthologues in human. The Hanks and Hunter protein kinase subfamily to which chimp kinases belong is also mentioned. [file 1471-2164-9-625-S3.rtf]

Additional file 3: List of chimpanzee protein kinases and their putative orthologues in human. The Hank and Hunter protein kinase subfamily to which they belong is also mentioned beside the gene identifier.
Chimpanzee	Human	
ENSPTRP00000015275 CC15_YEAST/25-272(kinase)	ENSP00000261716 CC15_YEAST/25-272(kinase)	
ENSPTRP00000030121 Jnk1(cmgc2)	ENSP00000321410 Jnk1(cmgc2)	
ENSPTRP00000034032 5Cdk(cmgc1)	ENSP00000297518 5Cdk(cmgc1)	
ENSPTRP00000039686 PBS2(mek_ste7)	ENSP00000351997 PBS2(mek_ste7)	
ENSPTRP00000008632 2Cdk(cmgc1)	ENSP00000243067 2Cdk(cmgc1)	
ENSPTRP00000030075 cmgc5(cmgc5)	ENSP00000316948 cmgc5(cmgc5)	
ENSPTRP00000041645 STE20(pak)	ENSP00000262836 STE20(pak)	
ENSPTRP00000009043 2PCTAIRE(cmgc1)	ENSP00000261211 2PCTAIRE(cmgc1)	
ENSPTRP00000036008 TrkB(ptk19)	ENSP00000277120 TrkB(ptk19)	
ENSPTRP00000046543 YCK2(ck1)	ENSP00000334735 YCK2(ck1)	
ENSPTRP00000020330 mPKC(agc2)	ENSP00000234179 mPKC(agc2)	
ENSPTRP00000004592 IIgCaMK(camk1)	ENSP00000315599 IIgCaMK(camk1)	
ENSPTRP00000053409 ST20_YEAST/620-871(kinase)	ENSP00000365716 ST20_YEAST/620-871(kinase)	
ENSPTRP00000031617 FYN(ptk1)	ENSP00000229470 FYN(ptk1)	
ENSPTRP00000052236 Ror1(ptk19)	ENSP00000360120 Ror1(ptk19)	
ENSPTRP00000001359 AKIN10(camk2)	ENSP00000360290 AKIN10(camk2)	
ENSPTRP00000028140 IIdCaMK(camk1)	ENSP00000339740 IIdCaMK(camk1)	
ENSPTRP00000054505 YCK2(ck1)	ENSP00000353904 YCK2(ck1)	
ENSPTRP00000015201 6FpMPK(cmgc2)	ENSP00000262393 6FpMPK(cmgc2)	
ENSPTRP00000030610 ptk20(ptk20)	ENSP00000318217 ptk20(ptk20)	
ENSPTRP00000021058 NINC_DROME/16-282(kinase)	ENSP00000281111 NINC_DROME/16-282(kinase)	
ENSPTRP00000025456 ActRIIB(tgfb)	ENSP00000340361 ActRIIB(tgfb)	
ENSPTRP00000040410 DdMLCK(camk1)	ENSP00000352810 DdMLCK(camk1)	
ENSPTRP00000048213 AKIN10(camk2)	ENSP00000346148 AKIN10(camk2)	
ENSPTRP00000054137 sp|Q95KR7|LCK_SAISC(PTK8)	ENSP00000348295 sp|Q95KR7|LCK_SAISC(PTK8)	
ENSPTRP00000012334 mek(mek_ste7)	ENSP00000346493 mek(mek_ste7)	
ENSPTRP00000016135 IIdCaMK(camk1)	ENSP00000340800 IIdCaMK(camk1)	
ENSPTRP00000030913 HOG1(cmgc2)	ENSP00000229794 HOG1(cmgc2)	
ENSPTRP00000012237 CKId(ck1)	ENSP00000305777 CKId(ck1)	
ENSPTRP00000034600 1KIN(camk2)	ENSP00000369030 1KIN(camk2)	
ENSPTRP00000046608 sp|Q95KR7|LCK_SAISC(PTK8)	ENSP00000276497 sp|Q95KR7|LCK_SAISC(PTK8)	
ENSPTRP00000026868 iPKC(agc2)	ENSP00000295797 iPKC(agc2)	
ENSPTRP00000018062 agc1(agc1)	ENSP00000309591 agc1(agc1)	
ENSPTRP00000004325 PKG-I(agc1)	ENSP00000327642 PKG-I(agc1)	
ENSPTRP00000050982 agc3(agc3)	ENSP00000263826 agc3(agc3)	
ENSPTRP00000021059 NINC_DROME/16-282(kinase)	ENSP00000314363 NINC_DROME/16-282(kinase)	
ENSPTRP00000017483 IIdCaMK(camk1)	ENSP00000301264 IIdCaMK(camk1)	
ENSPTRP00000009752 FpCdc2(cmgc1)	ENSP00000370938 FpCdc2(cmgc1)	
ENSPTRP00000052141 cAPKb(agc1)	ENSP00000359715 cAPKb(agc1)	
ENSPTRP00000031615 sp|Q95KR7|LCK_SAISC(PTK8)	ENSP00000357656 sp|Q95KR7|LCK_SAISC(PTK8)	
ENSPTRP00000018824 YAK1(cmgc5)	ENSP00000312789 YAK1(cmgc5)	
ENSPTRP00000025375 tgfb(tgfb)	ENSP00000295754 tgfb(tgfb)	
ENSPTRP00000017496 MEK2(mek_ste7)	ENSP00000262948 MEK2(mek_ste7)	
ENSPTRP00000012302 mek(mek_ste7)	ENSP00000302486 mek(mek_ste7)	
ENSPTRP00000028713 DdMLCK(camk1)	ENSP00000339291 DdMLCK(camk1)	
ENSPTRP00000041638 TSR-1(tgfb)	ENSP00000267008 TSR-1(tgfb)	
ENSPTRP00000052142 cAPKb(agc1)	ENSP00000359716 cAPKb(agc1)	
ENSPTRP00000040515 HRR25(ck1)	ENSP00000352929 HRR25(ck1)	
ENSPTRP00000012102 3ERK(cmgc2)	ENSP00000261845 3ERK(cmgc2)	
ENSPTRP00000029020 CAK/MO15(cmgc1)	ENSP00000256443 CAK/MO15(cmgc1)	
ENSPTRP00000037496 1PCTAIRE(cmgc1)	ENSP00000276052 1PCTAIRE(cmgc1)	
ENSPTRP00000004589 IIgCaMK(camk1)	ENSP00000307082 IIgCaMK(camk1)	
ENSPTRP00000004591 IIgCaMK(camk1)	ENSP00000319060 IIgCaMK(camk1)	
ENSPTRP00000027855 PKG-II(agc1)	ENSP00000264399 PKG-II(agc1)	
ENSPTRP00000008170 agc_other(agc_other)	ENSP00000373683 agc_other(agc_other)	
ENSPTRP00000003028 NrkA(nima)	ENSP00000356355 NrkA(nima)	
ENSPTRP00000006793 1bARK(agc4)	ENSP00000312262 1bARK(agc4)	
ENSPTRP00000032698 IIdCaMK(camk1)	ENSP00000369096 IIdCaMK(camk1)	
ENSPTRP00000042220 DdMLCK(camk1)	ENSP00000354089 DdMLCK(camk1)	
ENSPTRP00000036200 ALK-5(tgfb)	ENSP00000364133 ALK-5(tgfb)	
ENSPTRP00000004326 PKG-I(agc1)	ENSP00000363092 PKG-I(agc1)	
ENSPTRP00000042330 cAPKb(agc1)	ENSP00000359719 cAPKb(agc1)	
ENSPTRP00000027906 Jnk1(cmgc2)	ENSP00000352157 Jnk1(cmgc2)	
ENSPTRP00000033866 raf(raf)	ENSP00000288602 raf(raf)	
ENSPTRP00000008631 2Cdk(cmgc1)	ENSP00000266970 2Cdk(cmgc1)	
ENSPTRP00000033192 6Cdk(cmgc1)	ENSP00000265734 6Cdk(cmgc1)	
ENSPTRP00000048210 KIN1_SCHPO/125-395(kinase)	ENSP00000296800 KIN1_SCHPO/125-395(kinase)	
ENSPTRP00000012230 M-MLCK(camk1)	ENSP00000261891 M-MLCK(camk1)	
ENSPTRP00000038918 tgfb(tgfb)	ENSP00000351905 tgfb(tgfb)	
ENSPTRP00000012335 mek(mek_ste7)	ENSP00000342101 mek(mek_ste7)	
ENSPTRP00000049145 ALK-5(tgfb)	ENSP00000364129 ALK-5(tgfb)	
ENSPTRP00000009841 ICamK(camk1)	ENSP00000353846 ICamK(camk1)	
ENSPTRP00000018823 H2-PSK(cmgc5)	ENSP00000221803 H2-PSK(cmgc5)	
ENSPTRP00000014082 PSK-H1(camk1)	ENSP00000291041 PSK-H1(camk1)	
ENSPTRP00000009350 Braf(raf)	ENSP00000339952 Braf(raf)	
ENSPTRP00000016085 agc6(agc6)	ENSP00000225577 agc6(agc6)	
ENSPTRP00000008474 ALK-4(tgfb)	ENSP00000257963 ALK-4(tgfb)	
ENSPTRP00000012238 CKId(ck1)	ENSP00000307753 CKId(ck1)	
ENSPTRP00000024313 cmgc2(cmgc2)	ENSP00000215832 cmgc2(cmgc2)	
ENSPTRP00000029734 HRR25(ck1)	ENSP00000367074 HRR25(ck1)	
ENSPTRP00000031595 PHO85(cmgc1)	ENSP00000357907 PfC2R(cmgc1)	
ENSPTRP00000030613 ptk20(ptk20)	ENSP00000259875 ptk20(ptk20)	
ENSPTRP00000023923 H2-PSK(cmgc5)	ENSP00000340373 H2-PSK(cmgc5)	
ENSPTRP00000009840 ICamK(camk1)	ENSP00000255448 ICamK(camk1)	
ENSPTRP00000039845 cmgc1(cmgc1)	ENSP00000257904 cmgc1(cmgc1)	
ENSPTRP00000030914 HOG1(cmgc2)	ENSP00000308669 HOG1(cmgc2)	
ENSPTRP00000031477 KYK1_DICDI/1289-1559(kinase)	ENSP00000358338 KYK1_DICDI/1289-1559(kinase)	
ENSPTRP00000011290 agc6(agc6)	ENSP00000261991 agc6(agc6)	
ENSPTRP00000047919 HRR25(ck1)	ENSP00000367072 HRR25(ck1)	
ENSPTRP00000053525 a'CKII(cmgc4)	ENSP00000262506 a'CKII(cmgc4)	
ENSPTRP00000022158 Ehk-1(ptk11)	ENSP00000281821 Ehk-1(ptk11)	
ENSPTRP00000051728 G1-PSK(cmgc5)	ENSP00000357345 G1-PSK(cmgc5)	
ENSPTRP00000016236 aPKC(agc2)	ENSP00000284384 aPKC(agc2)	
ENSPTRP00000020039 DM(agc_other)	ENSP00000317985 DM(agc_other)	
ENSPTRP00000000706 NPK1(mekk_ste11)	ENSP00000350195 NPK1(mekk_ste11)	
ENSPTRP00000024020 p78(camk2)	ENSP00000270162 p78(camk2)	
ENSPTRP00000047818 Dror(ptk19)	ENSP00000297293 Dror(ptk19)	
ENSPTRP00000005308 FGFR-4(ptk15)	ENSP00000309878 FGFR-4(ptk15)	
ENSPTRP00000041728 p78(camk2)	ENSP00000335347 p78(camk2)	
ENSPTRP00000007716 Ctr1(raf)	ENSP00000252477 Ctr1(raf)	
ENSPTRP00000029300 FER(ptk4)	ENSP00000281092 FER(ptk4)	
ENSPTRP00000026398 DmCamKII(camk1)	ENSP00000346122 DmCamKII(camk1)	
ENSPTRP00000023474 FUSE_DROME/4-254(kinase)	ENSP00000216911 FUSE_DROME/4-254(kinase)	
ENSPTRP00000009777 Flt1(ptk14)	ENSP00000282397 Flt1(ptk14)	
ENSPTRP00000038032 ptk2(ptk2)	ENSP00000308176 ptk2(ptk2)	
ENSPTRP00000048967 Nek1(nima)	ENSP00000362702 Nek1(nima)	
ENSPTRP00000006632 ST20_YEAST/620-871(kinase)	ENSP00000294066 ST20_YEAST/620-871(kinase)	
ENSPTRP00000048177 H2-PSK(cmgc5)	ENSP00000368301 H2-PSK(cmgc5)	
ENSPTRP00000052464 MHK(cmgc_other)	ENSP00000355304 MHK(cmgc_other)	
ENSPTRP00000021456 ALK-5(tgfb)	ENSP00000335178 ALK-5(tgfb)	
ENSPTRP00000017040 3ERK(cmgc2)	ENSP00000269463 3ERK(cmgc2)	
ENSPTRP00000023839 1+kin(camk2)	ENSP00000270112 1+kin(camk2)	
ENSPTRP00000048957 1+pck(agc2)	ENSP00000343325 1+pck(agc2)	
ENSPTRP00000005852 HsWee1(wee1)	ENSP00000299613 HsWee1(wee1)	
ENSPTRP00000010729 ST20_YEAST/620-871(kinase)	ENSP00000013125 ST20_YEAST/620-871(kinase)	
ENSPTRP00000032825 MPhKg(camk1)	ENSP00000297373 MPhKg(camk1)	
ENSPTRP00000049076 TorRTK(ptk19)	ENSP00000363571 TorRTK(ptk19)	
ENSPTRP00000006703 PTK1(mlk)	ENSP00000309597 PTK1(mlk)	
ENSPTRP00000030947 PIM1_HUMAN/38-290(kinase)	ENSP00000362606 PIM1_HUMAN/38-290(kinase)	
ENSPTRP00000042372 SPK1_YEAST/198-466(kinase)	ENSP00000354877 SPK1_YEAST/198-466(kinase)	
ENSPTRP00000020411 1BCePKC(agc2)	ENSP00000306124 1BCePKC(agc2)	
ENSPTRP00000028220 FUSE_DROME/4-254(kinase)	ENSP00000270861 FUSE_DROME/4-254(kinase)	
ENSPTRP00000041216 thPKC(agc2)	ENSP00000263125 thPKC(agc2)	
ENSPTRP00000026879 NINC_DROME/16-282(kinase)	ENSP00000349880 NINC_DROME/16-282(kinase)	
ENSPTRP00000013479 bPKC(agc2)	ENSP00000305355 bPKC(agc2)	
ENSPTRP00000010886 ePKC(agc2)	ENSP00000329127 ePKC(agc2)	
ENSPTRP00000002493 ptk16(ptk16)	ENSP00000357178 ptk16(ptk16)	
ENSPTRP00000021817 DdMLCK(camk1)	ENSP00000263955 DdMLCK(camk1)	
ENSPTRP00000038191 STE20(pak)	ENSP00000353864 STE20(pak)	
ENSPTRP00000047442 IIgCaMK(camk1)	ENSP00000367405 IIgCaMK(camk1)	
ENSPTRP00000049722 ELK(ptk11)	ENSP00000339916 ELK(ptk11)	
ENSPTRP00000008285 Pto(plantrk)	ENSP00000349096 Pto(plantrk)	
ENSPTRP00000011852 ALK(ptk17)	ENSP00000263800 ALK(ptk17)	
ENSPTRP00000040121 Nek1(nima)	ENSP00000347767 Nek1(nima)	
ENSPTRP00000031081 sp|Q13308|PTK7_HUMAN(PTK18)	ENSP00000325462 sp|Q13308|PTK7_HUMAN(PTK18)	
ENSPTRP00000047337 Dsrc28(ptk2)	ENSP00000361946 Dsrc28(ptk2)	
ENSPTRP00000003266 PSK-H1(camk1)	ENSP00000009105 PSK-H1(camk1)	
ENSPTRP00000013108 wee1(wee1)	ENSP00000262300 wee1(wee1)	
ENSPTRP00000043904 NINC_DROME/16-282(kinase)	ENSP00000284483 NINC_DROME/16-282(kinase)	
ENSPTRP00000007339 p78(camk2)	ENSP00000305976 p78(camk2)	
ENSPTRP00000047612 H2-PSK(cmgc5)	ENSP00000343108 H2-PSK(cmgc5)	
ENSPTRP00000049582 NPK1(mekk_ste11)	ENSP00000356742 NPK1(mekk_ste11)	
ENSPTRP00000031079 sp|Q13308|PTK7_HUMAN(PTK18)	ENSP00000325992 sp|Q13308|PTK7_HUMAN(PTK18)	
ENSPTRP00000026129 Ehk-2(ptk11)	ENSP00000374323 Ehk-2(ptk11)	
ENSPTRP00000021530 ST20_YEAST/620-871(kinase)	ENSP00000348278 ST20_YEAST/620-871(kinase)	
ENSPTRP00000031658 ptk18(ptk18)	ENSP00000357494 ptk18(ptk18)	
ENSPTRP00000022716 STE20(pak)	ENSP00000322957 STE20(pak)	
ENSPTRP00000005302 FGFR-4(ptk15)	ENSP00000358056 FGFR-4(ptk15)	
ENSPTRP00000035593 G1-PSK(cmgc5)	ENSP00000328452 G1-PSK(cmgc5)	
ENSPTRP00000002496 ptk19(ptk19)	ENSP00000351486 ptk19(ptk19)	
ENSPTRP00000042082 HEK2(ptk11)	ENSP00000354191 HEK2(ptk11)	
ENSPTRP00000042420 MAK(cmgc_other)	ENSP00000313021 MAK(cmgc_other)	
ENSPTRP00000021455 ALK-5(tgfb)	ENSP00000243349 ALK-5(tgfb)	
ENSPTRP00000037557 PIM1_HUMAN/38-290(kinase)	ENSP00000365692 PIM1_HUMAN/38-290(kinase)	
ENSPTRP00000001943 H2-PSK(cmgc5)	ENSP00000340956 H2-PSK(cmgc5)	
ENSPTRP00000043214 Flg(ptk15)	ENSP00000311337 Flg(ptk15)	
ENSPTRP00000027637 ckit(ptk14)	ENSP00000288135 ckit(ptk14)	
ENSPTRP00000002684 TKT(ptk20)	ENSP00000356898 TKT(ptk20)	
ENSPTRP00000001162 M3K9_HUMAN/3-262(kinase)	ENSP00000361158 M3K9_HUMAN/3-262(kinase)	
ENSPTRP00000026525 Nek1(nima)	ENSP00000372857 Nek1(nima)	
ENSPTRP00000001607 1+pck(agc2)	ENSP00000354857 1+pck(agc2)	
ENSPTRP00000027008 HEK2(ptk11)	ENSP00000332118 HEK2(ptk11)	
ENSPTRP00000040692 Jnk1(cmgc2)	ENSP00000353483 Jnk1(cmgc2)	
ENSPTRP00000023967 pelle(plantrk)	ENSP00000332454 pelle(plantrk)	
ENSPTRP00000031633 sp|Q95KR7|LCK_SAISC(PTK8)	ENSP00000357615 sp|Q95KR7|LCK_SAISC(PTK8)	
ENSPTRP00000011800 GCN2(translationk)	ENSP00000372174 GCN2(translationk)	
ENSPTRP00000035490 JAK2(ptk7)	ENSP00000371067 JAK2(ptk7)	
ENSPTRP00000027683 Ehk-1(ptk11)	ENSP00000273854 Ehk-1(ptk11)	
ENSPTRP00000034767 Sgk(agc_other)	ENSP00000262211 Sgk(agc_other)	
ENSPTRP00000028937 polo(polo)	ENSP00000274289 polo(polo)	
ENSPTRP00000027268 Bek(ptk15)	ENSP00000339824 Bek(ptk15)	
ENSPTRP00000026393 M-MLCK(camk1)	ENSP00000320622 M-MLCK(camk1)	
ENSPTRP00000036065 Ror2(ptk19)	ENSP00000364867 Ror2(ptk19)	
ENSPTRP00000021061 NINC_DROME/16-282(kinase)	ENSP00000343658 NINC_DROME/16-282(kinase)	
ENSPTRP00000001213 K6A1_MOUSE/407-664(kinase)	ENSP00000339197 KCC4_MOUSE/42-296(kinase)	
ENSPTRP00000001494 KYK1_DICDI/1289-1559(kinase)	ENSP00000359928 KYK1_DICDI/1289-1559(kinase)	
ENSPTRP00000009430 KIN1_SCHPO/125-395(kinase)	ENSP00000312741 KIN1_SCHPO/125-395(kinase)	
ENSPTRP00000034905 Ctr1(raf)	ENSP00000220751 Ctr1(raf)	
ENSPTRP00000004727 ALK-3(tgfb)	ENSP00000224764 ALK-3(tgfb)	
ENSPTRP00000004033 Sgk(agc_other)	ENSP00000365107 Sgk(agc_other)	
ENSPTRP00000049030 M3K9_HUMAN/3-262(kinase)	ENSP00000340257 M3K9_HUMAN/3-262(kinase)	
ENSPTRP00000049753 DdMLCK(camk1)	ENSP00000372023 DdMLCK(camk1)	
ENSPTRP00000019871 FUSE_DROME/4-254(kinase)	ENSP00000302898 FUSE_DROME/4-254(kinase)	
ENSPTRP00000015508 ErbB2(ptk10)	ENSP00000269571 ErbB2(ptk10)	
ENSPTRP00000029704 cAPKg(agc1)	ENSP00000303165 cAPKg(agc1)	
ENSPTRP00000041743 sp|Q14289|FAK2_HUMAN(PTK9)	ENSP00000346424 sp|Q14289|FAK2_HUMAN(PTK9)	
ENSPTRP00000025450 ST20_YEAST/620-871(kinase)	ENSP00000311713 ST20_YEAST/620-871(kinase)	
ENSPTRP00000003352 p78(camk2)	ENSP00000355885 p78(camk2)	
ENSPTRP00000027269 Bek(ptk15)	ENSP00000231803 Bek(ptk15)	
ENSPTRP00000031907 agc_other(agc_other)	ENSP00000253339 agc_other(agc_other)	
ENSPTRP00000000680 2RSKN(agc6)	ENSP00000363277 2RSKN(agc6)	
ENSPTRP00000002874 ARG(ptk5)	ENSP00000339209 Dabl(ptk5)	
ENSPTRP00000014968 MEK2(mek_ste7)	ENSP00000262445 MEK2(mek_ste7)	
ENSPTRP00000003206 H2-PSK(cmgc5)	ENSP00000356076 H2-PSK(cmgc5)	
ENSPTRP00000021606 M3K9_HUMAN/3-262(kinase)	ENSP00000364361 M3K9_HUMAN/3-262(kinase)	
ENSPTRP00000047441 IIgCaMK(camk1)	ENSP00000367421 IIgCaMK(camk1)	
ENSPTRP00000032646 CHED(cmgc_other)	ENSP00000181839 CHED(cmgc_other)	
ENSPTRP00000017320 SNF1(camk2)	ENSP00000324856 SNF1(camk2)	
ENSPTRP00000028331 DmCamKII(camk1)	ENSP00000303887 DmCamKII(camk1)	
ENSPTRP00000042882 Ctr1(raf)	ENSP00000343940 Ctr1(raf)	
ENSPTRP00000012792 ptk16(ptk16)	ENSP00000268035 ptk16(ptk16)	
ENSPTRP00000043617 cmgc5(cmgc5)	ENSP00000349935 cmgc5(cmgc5)	
ENSPTRP00000031479 Ehk-1(ptk11)	ENSP00000358309 Ehk-1(ptk11)	
ENSPTRP00000011798 GCN2(translationk)	ENSP00000263791 GCN2(translationk)	
ENSPTRP00000011929 TTBK2_Mm(ck1)	ENSP00000263802 CKId(ck1)	
ENSPTRP00000044900 Jnk1(cmgc2)	ENSP00000362875 Jnk1(cmgc2)	
ENSPTRP00000053410 ST20_YEAST/620-871(kinase)	ENSP00000365730 ST20_YEAST/620-871(kinase)	
ENSPTRP00000007718 Ctr1(raf)	ENSP00000313059 Ctr1(raf)	
ENSPTRP00000018979 3bGSK(cmgc3)	ENSP00000222330 3bGSK(cmgc3)	
ENSPTRP00000012456 G1-PSK(cmgc5)	ENSP00000344112 G1-PSK(cmgc5)	
ENSPTRP00000046634 M-MLCK(camk1)	ENSP00000353452 M-MLCK(camk1)	
ENSPTRP00000031790 Sgk(agc_other)	ENSP00000237305 Sgk(agc_other)	
ENSPTRP00000049774 2bARK(agc4)	ENSP00000317578 2bARK(agc4)	
ENSPTRP00000033165 3PCTAIRE(cmgc1)	ENSP00000302071 1PCTAIRE(cmgc1)	
ENSPTRP00000005306 Bek(ptk15)	ENSP00000358061 Bek(ptk15)	
ENSPTRP00000026645 6GRK(agc4)	ENSP00000264952 6GRK(agc4)	
ENSPTRP00000010577 mPKC(agc2)	ENSP00000333568 mPKC(agc2)	
ENSPTRP00000005305 FGFR-4(ptk15)	ENSP00000358054 FGFR-4(ptk15)	
ENSPTRP00000031791 Sgk(agc_other)	ENSP00000356831 Sgk(agc_other)	
ENSPTRP00000039533 PBS2(mek_ste7)	ENSP00000319139 PBS2(mek_ste7)	
ENSPTRP00000028863 CDR1_SCHPO/12-258(kinase)	ENSP00000313572 CDR1_SCHPO/12-258(kinase)	
ENSPTRP00000019126 p78(camk2)	ENSP00000262893 p78(camk2)	
ENSPTRP00000025528 p78(camk2)	ENSP00000296088 p78(camk2)	
ENSPTRP00000042756 p78(camk2)	ENSP00000303698 p78(camk2)	
ENSPTRP00000027988 ALK-6(tgfb)	ENSP00000264568 ALK-6(tgfb)	
ENSPTRP00000049596 Sgk(agc_other)	ENSP00000356832 Sgk(agc_other)	
ENSPTRP00000003207 MAPKAP2(camk_other)	ENSP00000356070 MAPKAP2(camk_other)	
ENSPTRP00000033588 ptk21(ptk21)	ENSP00000317272 ptk21(ptk21)	
ENSPTRP00000028467 Nek1(nima)	ENSP00000336715 Nek1(nima)	
ENSPTRP00000034983 ST20_YEAST/620-871(kinase)	ENSP00000347009 ST20_YEAST/620-871(kinase)	
ENSPTRP00000009145 SNF1(camk2)	ENSP00000261402 SNF1(camk2)	
ENSPTRP00000021405 ActRIIA(tgfb)	ENSP00000241416 ActRIIA(tgfb)	
ENSPTRP00000035769 KYK2_DICDI/108-364(kinase)	ENSP00000338127 KYK2_DICDI/108-364(kinase)	
ENSPTRP00000030929 agc_other(agc_other)	ENSP00000229812 agc_other(agc_other)	
ENSPTRP00000016134 IIdCaMK(camk1)	ENSP00000275780 IIdCaMK(camk1)	
ENSPTRP00000036748 ARG(ptk5)	ENSP00000361423 ARG(ptk5)	
ENSPTRP00000029494 KKIALRE(cmgc_other)	ENSP00000265334 KKIALRE(cmgc_other)	
ENSPTRP00000031399 TTK_HUMAN/509-775(kinase)	ENSP00000230510 TTK_HUMAN/509-775(kinase)	
ENSPTRP00000004353 CDC2Hs(cmgc1)	ENSP00000306043 CDC2Hs(cmgc1)	
ENSPTRP00000035264 sp|Q14289|FAK2_HUMAN(PTK9)	ENSP00000341189 sp|Q14289|FAK2_HUMAN(PTK9)	
ENSPTRP00000044443 NPK1(mekk_ste11)	ENSP00000348104 NPK1(mekk_ste11)	
ENSPTRP00000039569 sp|Q13308|PTK7_HUMAN(PTK18)	ENSP00000352840 sp|Q13308|PTK7_HUMAN(PTK18)	
ENSPTRP00000031080 sp|Q13308|PTK7_HUMAN(PTK18)	ENSP00000230419 sp|Q13308|PTK7_HUMAN(PTK18)	
ENSPTRP00000027164 STE20(pak)	ENSP00000314067 STE20(pak)	
ENSPTRP00000052704 HEK2(ptk11)	ENSP00000363761 HEK2(ptk11)	
ENSPTRP00000034416 sp|Q14289|FAK2_HUMAN(PTK9)	ENSP00000332816 sp|Q14289|FAK2_HUMAN(PTK9)	
ENSPTRP00000012469 SPK1_YEAST/198-466(kinase)	ENSP00000268074 SPK1_YEAST/198-466(kinase)	
ENSPTRP00000011923 TTBK2_Mm(ck1)	ENSP00000267890 CKId(ck1)	
ENSPTRP00000025842 MAPKAP2(camk_other)	ENSP00000350639 MAPKAP2(camk_other)	
ENSPTRP00000029317 IVCaMK(camk1)	ENSP00000282356 IVCaMK(camk1)	
ENSPTRP00000022026 ErbB4(ptk10)	ENSP00000342235 ErbB4(ptk10)	
ENSPTRP00000003293 KIN3(nima)	ENSP00000355966 KIN3(nima)	
ENSPTRP00000009705 agc_other(agc_other)	ENSP00000372035 agc_other(agc_other)	
ENSPTRP00000025789 RON(ptk21)	ENSP00000341325 RON(ptk21)	
ENSPTRP00000030194 prp1+(cmgc5)	ENSP00000369540 prp1+(cmgc5)	
ENSPTRP00000005141 ST20_YEAST/620-871(kinase)	ENSP00000336824 ST20_YEAST/620-871(kinase)	
ENSPTRP00000031083 ptk18(ptk18)	ENSP00000324119 ptk18(ptk18)	
ENSPTRP00000027639 Flk1(ptk14)	ENSP00000263923 Flk1(ptk14)	
ENSPTRP00000008537 C14(tgfb)	ENSP00000257863 C14(tgfb)	
ENSPTRP00000016137 IIdCaMK(camk1)	ENSP00000316512 IIdCaMK(camk1)	
ENSPTRP00000018854 agc3(agc3)	ENSP00000309428 agc3(agc3)	
ENSPTRP00000030180 DtSpk-1(ptk2)	ENSP00000259808 DtSpk-1(ptk2)	
ENSPTRP00000011851 ALK(ptk17)	ENSP00000347293 ALK(ptk17)	
ENSPTRP00000025252 raf(raf)	ENSP00000251849 raf(raf)	
ENSPTRP00000030916 1FpMPK(cmgc2)	ENSP00000211287 1FpMPK(cmgc2)	
ENSPTRP00000025422 ICamK(camk1)	ENSP00000295943 ICamK(camk1)	
ENSPTRP00000002495 ptk19(ptk19)	ENSP00000357180 ptk19(ptk19)	
ENSPTRP00000049246 M-MLCK(camk1)	ENSP00000350785 M-MLCK(camk1)	
ENSPTRP00000034701 KMOS_CERAE/60-338(kinase)	ENSP00000310722 KMOS_CERAE/60-338(kinase)	
ENSPTRP00000012756 ptk4(ptk4)	ENSP00000331504 ptk4(ptk4)	
ENSPTRP00000021171 c-Eyk(ptk12)	ENSP00000295408 c-Eyk(ptk12)	
ENSPTRP00000006052 H2-PSK(cmgc5)	ENSP00000304226 H2-PSK(cmgc5)	
ENSPTRP00000033166 3PCTAIRE(cmgc1)	ENSP00000369390 1PCTAIRE(cmgc1)	
ENSPTRP00000005303 FGFR-4(ptk15)	ENSP00000358055 FGFR-4(ptk15)	
ENSPTRP00000004034 Sgk(agc_other)	ENSP00000343446 Sgk(agc_other)	
ENSPTRP00000001210 PSK-H1(camk1)	ENSP00000361013 PSK-H1(camk1)	
ENSPTRP00000052114 ST20_YEAST/620-871(kinase)	ENSP00000361892 ST20_YEAST/620-871(kinase)	
ENSPTRP00000034768 Sgk(agc_other)	ENSP00000331816 Sgk(agc_other)	
ENSPTRP00000016847 DM(agc_other)	ENSP00000261535 DM(agc_other)	
ENSPTRP00000027312 IT11(agc4)	ENSP00000264763 IT11(agc4)	
ENSPTRP00000027681 Ehk-1(ptk11)	ENSP00000346899 Ehk-1(ptk11)	
ENSPTRP00000028468 Nek1(nima)	ENSP00000309742 Nek1(nima)	
ENSPTRP00000016724 sp|Q95KR7|LCK_SAISC(PTK8)	ENSP00000324740 sp|Q95KR7|LCK_SAISC(PTK8)	
ENSPTRP00000001185 Mast205(agc_other)	ENSP00000361078 Mast205(agc_other)	
ENSPTRP00000003885 ICamK(camk1)	ENSP00000368124 ICamK(camk1)	
ENSPTRP00000007593 SNF1(camk2)	ENSP00000278916 SNF1(camk2)	
ENSPTRP00000000847 AKIN10(camk2)	ENSP00000362634 AKIN10(camk2)	
ENSPTRP00000025788 RON(ptk21)	ENSP00000296474 RON(ptk21)	
ENSPTRP00000051478 ARG(ptk5)	ENSP00000356595 ARG(ptk5)	
ENSPTRP00000008798 K-MLCK(camk1)	ENSP00000329967 K-MLCK(camk1)	
ENSPTRP00000032807 ptk10(ptk10)	ENSP00000275493 ptk10(ptk10)	
ENSPTRP00000008819 H2-PSK(cmgc5)	ENSP00000342105 H2-PSK(cmgc5)	
ENSPTRP00000021008 ZAP70(ptk6)	ENSP00000264972 ZAP70(ptk6)	
ENSPTRP00000003319 agc6(agc6)	ENSP00000355927 PKD1_DICDI/36-291(kinase)	
ENSPTRP00000041316 M-MLCK(camk1)	ENSP00000365093 M-MLCK(camk1)	
ENSPTRP00000018851 PTK1(mlk)	ENSP00000253055 PTK1(mlk)	
ENSPTRP00000021858 cmgc5(cmgc5)	ENSP00000326830 cmgc5(cmgc5)	
ENSPTRP00000035184 nim1+(camk2)	ENSP00000312150 nim1+(camk2)	
ENSPTRP00000026114 HEK(ptk11)	ENSP00000337451 HEK(ptk11)	
ENSPTRP00000024574 FER(ptk4)	ENSP00000332687 FER(ptk4)	
ENSPTRP00000001085 Tek(ptk13)	ENSP00000361554 Tek(ptk13)	
ENSPTRP00000021454 ALK-5(tgfb)	ENSP00000335139 ALK-5(tgfb)	
ENSPTRP00000006610 agc6(agc6)	ENSP00000294261 agc6(agc6)	
ENSPTRP00000013475 polo(polo)	ENSP00000300093 polo(polo)	
ENSPTRP00000031089 TTBK2_Hs(ck1)	ENSP00000307357 CKId(ck1)	
ENSPTRP00000048858 C14(tgfb)	ENSP00000363708 C14(tgfb)	
ENSPTRP00000037688 Ctr1(raf)	ENSP00000346667 Ctr1(raf)	
ENSPTRP00000034417 sp|Q14289|FAK2_HUMAN(PTK9)	ENSP00000342242 sp|Q14289|FAK2_HUMAN(PTK9)	
ENSPTRP00000045541 Nek1(nima)	ENSP00000368363 Nek1(nima)	
ENSPTRP00000001941 H2-PSK(cmgc5)	ENSP00000358567 H2-PSK(cmgc5)	
ENSPTRP00000020876 PKR(translationk)	ENSP00000307235 PKR(translationk)	
ENSPTRP00000008254 M3K9_HUMAN/3-262(kinase)	ENSP00000298910 M3K9_HUMAN/3-262(kinase)	
ENSPTRP00000009370 DM(agc_other)	ENSP00000261833 DM(agc_other)	
ENSPTRP00000012458 G1-PSK(cmgc5)	ENSP00000323106 G1-PSK(cmgc5)	
ENSPTRP00000035809 p78(camk2)	ENSP00000298048 p78(camk2)	
ENSPTRP00000001211 PSK-H1(camk1)	ENSP00000361014 PSK-H1(camk1)	
ENSPTRP00000030195 YAK1(cmgc5)	ENSP00000283003 YAK1(cmgc5)	
ENSPTRP00000012676 TrkC(ptk19)	ENSP00000354207 TrkC(ptk19)	
ENSPTRP00000027232 polo_real(polo)	ENSP00000314499 polo_real(polo)	
ENSPTRP00000013638 CC15_YEAST/25-272(kinase)	ENSP00000310094 CC15_YEAST/25-272(kinase)	
ENSPTRP00000001425 JAK1(ptk7)	ENSP00000294423 JAK1(ptk7)	
ENSPTRP00000032664 DdMLCK(camk1)	ENSP00000319192 DdMLCK(camk1)	
ENSPTRP00000003354 p78(camk2)	ENSP00000355884 p78(camk2)	
ENSPTRP00000026315 3bGSK(cmgc3)	ENSP00000324806 3bGSK(cmgc3)	
ENSPTRP00000046906 ST20_YEAST/620-871(kinase)	ENSP00000325748 ST20_YEAST/620-871(kinase)	
ENSPTRP00000048901 JAK2(ptk7)	ENSP00000222246 JAK2(ptk7)	
ENSPTRP00000016424 3Cdk(cmgc1)	ENSP00000293215 3Cdk(cmgc1)	
ENSPTRP00000009429 KIN1_SCHPO/125-395(kinase)	ENSP00000321230 KIN1_SCHPO/125-395(kinase)	
ENSPTRP00000049751 DdMLCK(camk1)	ENSP00000372021 DdMLCK(camk1)	
ENSPTRP00000009277 IVCaMK(camk1)	ENSP00000202788 IVCaMK(camk1)	
ENSPTRP00000035268 ptk9(ptk9)	ENSP00000342839 ptk9(ptk9)	
ENSPTRP00000021693 Titen(camk1)	ENSP00000343764 Titen(camk1)	
ENSPTRP00000013693 TPhKg(camk1)	ENSP00000329968 TPhKg(camk1)	
ENSPTRP00000024456 DdMLCK(camk1)	ENSP00000329178 DdMLCK(camk1)	
ENSPTRP00000005139 ST20_YEAST/620-871(kinase)	ENSP00000358770 ST20_YEAST/620-871(kinase)	
ENSPTRP00000031242 MAK(cmgc_other)	ENSP00000308912 MAK(cmgc_other)	
ENSPTRP00000033846 H2-PSK(cmgc5)	ENSP00000263551 H2-PSK(cmgc5)	
ENSPTRP00000045362 kinase(kinase)	ENSP00000351781 1RSKN(agc6)	
ENSPTRP00000021060 NINC_DROME/16-282(kinase)	ENSP00000313644 NINC_DROME/16-282(kinase)	
ENSPTRP00000015697 Ctr1(raf)	ENSP00000246914 Ctr1(raf)	
ENSPTRP00000006810 agc6(agc6)	ENSP00000308413 agc6(agc6)	
ENSPTRP00000020055 IIdCaMK(camk1)	ENSP00000155926 IIdCaMK(camk1)	
ENSPTRP00000004070 ST20_YEAST/620-871(kinase)	ENSP00000263056 ST20_YEAST/620-871(kinase)	
ENSPTRP00000009862 HRR25(ck1)	ENSP00000322723 HRR25(ck1)	
ENSPTRP00000003173 IIApl(agc2)	ENSP00000356130 IIApl(agc2)	
ENSPTRP00000025169 ICamK(camk1)	ENSP00000256460 ICamK(camk1)	
ENSPTRP00000023666 sp|Q95KR7|LCK_SAISC(PTK8)	ENSP00000217188 sp|Q95KR7|LCK_SAISC(PTK8)	
ENSPTRP00000023198 Sgk(agc_other)	ENSP00000340608 Sgk(agc_other)	
ENSPTRP00000029829 Itk/Tsk(ptk2)	ENSP00000231189 Itk/Tsk(ptk2)	
ENSPTRP00000033933 EPH(ptk11)	ENSP00000275815 EPH(ptk11)	
ENSPTRP00000021457 TskL7(tgfb)	ENSP00000263640 TskL7(tgfb)	
ENSPTRP00000052113 ST20_YEAST/620-871(kinase)	ENSP00000361887 ST20_YEAST/620-871(kinase)	
ENSPTRP00000027601 ptk2(ptk2)	ENSP00000258070 ptk2(ptk2)	
ENSPTRP00000026882 NINC_DROME/16-282(kinase)	ENSP00000345352 NINC_DROME/16-282(kinase)	
ENSPTRP00000012464 ptk3(ptk3)	ENSP00000220003 ptk3(ptk3)	
ENSPTRP00000025935 Nek1(nima)	ENSP00000233027 Nek1(nima)	
ENSPTRP00000036573 KIN28(cmgc1)	ENSP00000362362 KIN28(cmgc1)	
ENSPTRP00000003583 PTK1(mlk)	ENSP00000355583 PTK1(mlk)	
ENSPTRP00000035635 Tek(ptk13)	ENSP00000369375 Tek(ptk13)	
ENSPTRP00000024203 AKIN10(camk2)	ENSP00000329016 AKIN10(camk2)	
ENSPTRP00000045357 YAK1(cmgc5)	ENSP00000352687 YAK1(cmgc5)	
ENSPTRP00000014877 KAKT_MLVAT/171-429(kinase)	ENSP00000313950 KAKT_MLVAT/171-429(kinase)	
ENSPTRP00000030173 M-MLCK(camk1)	ENSP00000274643 M-MLCK(camk1)	
ENSPTRP00000045642 M-MLCK(camk1)	ENSP00000354004 M-MLCK(camk1)	
ENSPTRP00000036064 Ror2(ptk19)	ENSP00000364860 Ror2(ptk19)	
ENSPTRP00000042674 3PCTAIRE(cmgc1)	ENSP00000342589 3PCTAIRE(cmgc1)	
ENSPTRP00000021569 NINC_DROME/16-282(kinase)	ENSP00000263810 NINC_DROME/16-282(kinase)	
ENSPTRP00000029005 Mast205(agc_other)	ENSP00000261569 Mast205(agc_other)	
ENSPTRP00000014648 p78(camk2)	ENSP00000371188 YCL24(camk2)	
ENSPTRP00000017670 INS.R(ptk16)	ENSP00000303830 INS.R(ptk16)	
ENSPTRP00000027267 ptk3(ptk3)	ENSP00000354170 ptk3(ptk3)	
ENSPTRP00000014651 YCL24(camk2)	ENSP00000158166 YCL24(camk2)	
ENSPTRP00000008543 M3K9_HUMAN/3-262(kinase)	ENSP00000267079 M3K9_HUMAN/3-262(kinase)	
ENSPTRP00000049168 byr2(mekk_ste11)	ENSP00000365004 byr2(mekk_ste11)	
ENSPTRP00000025492 kinase(kinase)	ENSP00000264459 unclassified(kinase);ENSP00000307581unclassified(kinase)	
ENSPTRP00000018861 YAK1(cmgc5)	ENSP00000291823 YAK1(cmgc5)	
ENSPTRP00000010533 SNF1(camk2)	ENSP00000339179 SNF1(camk2)	
ENSPTRP00000010556 Ctr1(raf)	ENSP00000216274 KSYK_PIG/364-619(kinase)	
ENSPTRP00000013476 AKIN10(camk2)	ENSP00000256797 AKIN10(camk2)	
ENSPTRP00000020323 PKR(translationk)	ENSP00000341954 PKR(translationk)	
ENSPTRP00000010028 Nek1(nima)	ENSP00000258597 Nek1(nima)	
ENSPTRP00000009773 bPDGFR(ptk14)	ENSP00000370369 bPDGFR(ptk14)	
ENSPTRP00000048865 KIN28(cmgc1)	ENSP00000363726 KIN28(cmgc1)	
ENSPTRP00000043649 KAB7_YEAST/1096-1354(kinase)	ENSP00000351475 KAB7_YEAST/1096-1354(kinase)	
ENSPTRP00000020319 PKR(translationk)	ENSP00000233057 PKR(translationk)	
ENSPTRP00000025934 Nek1(nima)	ENSP00000373227 Nek1(nima)	
ENSPTRP00000010728 KKIALRE(cmgc_other)	ENSP00000216378 KKIALRE(cmgc_other)	
ENSPTRP00000035898 cAPKb(agc1)	ENSP00000297734 cAPKb(agc1)	
ENSPTRP00000023968 pelle(plantrk)	ENSP00000330161 pelle(plantrk)	
ENSPTRP00000049167 byr2(mekk_ste11)	ENSP00000365005 byr2(mekk_ste11)	
ENSPTRP00000006811 agc6(agc6)	ENSP00000365923 agc6(agc6)	
ENSPTRP00000048966 NrkA(nima)	ENSP00000362703 NrkA(nima)	
ENSPTRP00000031088 TTBK2_Hs(ck1)	ENSP00000259750 CKId(ck1)	
ENSPTRP00000032971 1SRK(ptk1)	ENSP00000336740 LYN(ptk1)	
ENSPTRP00000027014 KYK2_DICDI/108-364(kinase)	ENSP00000265026 M3K9_HUMAN/3-262(kinase)	
ENSPTRP00000021901 3PCTAIRE(cmgc1)	ENSP00000260967 3PCTAIRE(cmgc1)	
ENSPTRP00000042821 Titen(camk1)	ENSP00000353530 2CRSK(camk1)	
ENSPTRP00000041788 NINC_DROME/16-282(kinase)	ENSP00000347427 NINC_DROME/16-282(kinase)	
ENSPTRP00000021572 NINC_DROME/16-282(kinase)	ENSP00000314650 NINC_DROME/16-282(kinase)	
ENSPTRP00000044542 byr2(mekk_ste11)	ENSP00000351140 byr2(mekk_ste11)	
ENSPTRP00000035632 Tek(ptk13)	ENSP00000343716 Tek(ptk13)	
ENSPTRP00000014699 NINC_DROME/16-282(kinase)	ENSP00000269296 NINC_DROME/16-282(kinase)	
ENSPTRP00000014805 ptk8(ptk8)	ENSP00000312309 ptk8(ptk8)	
ENSPTRP00000042257 NINC_DROME/16-282(kinase)	ENSP00000335100 NINC_DROME/16-282(kinase)	
ENSPTRP00000043392 IIaCaMK(camk1)	ENSP00000320754 IIaCaMK(camk1)	
ENSPTRP00000036652 1+pck(agc2)	ENSP00000291906 1+pck(agc2)	
ENSPTRP00000047639 PRO25(plantrk)	ENSP00000306678 KYK2_DICDI/108-364(kinase)	
ENSPTRP00000022091 FUSE_DROME/4-254(kinase)	ENSP00000295709 FUSE_DROME/4-254(kinase)	
ENSPTRP00000007788 H2-PSK(cmgc5)	ENSP00000010132 H2-PSK(cmgc5)	
ENSPTRP00000047059 Ctr1(raf)	ENSP00000371997 KSYK_PIG/364-619(kinase)	
ENSPTRP00000053553 CDC5(polo)	ENSP00000264889 CDC5(polo)	
ENSPTRP00000034602 SPK1_YEAST/198-466(kinase)	ENSP00000339151 SPK1_YEAST/198-466(kinase)	
ENSPTRP00000021280 NPK1(mekk_ste11)	ENSP00000343463 NPK1(mekk_ste11)	
ENSPTRP00000013797 M-MLCK(camk1)	ENSP00000268446 M-MLCK(camk1)	
ENSPTRP00000052022 KKIALRE(cmgc_other)	ENSP00000368080 KKIALRE(cmgc_other)	
ENSPTRP00000011015 PTK1(mlk)	ENSP00000005198 PTK1(mlk)	
ENSPTRP00000029935 ST20_YEAST/620-871(kinase)	ENSP00000176763 ST20_YEAST/620-871(kinase)	
ENSPTRP00000020357 ST20_YEAST/620-871(kinase)	ENSP00000263881 ST20_YEAST/620-871(kinase)	
ENSPTRP00000020358 ST20_YEAST/620-871(kinase)	ENSP00000345434 ST20_YEAST/620-871(kinase)	
ENSPTRP00000022925 sp|Q95KR7|LCK_SAISC(PTK8)	ENSP00000262651 sp|Q95KR7|LCK_SAISC(PTK8)	
ENSPTRP00000045305 agc1(agc1)	ENSP00000344220 agc1(agc1)	
ENSPTRP00000040804 p78(camk2)	ENSP00000351687 p78(camk2)	
ENSPTRP00000048521 sp|Q95KR7|LCK_SAISC(PTK8)	ENSP00000259089 sp|Q95KR7|LCK_SAISC(PTK8)	
ENSPTRP00000022498 nim1+(camk2)	ENSP00000217233 nim1+(camk2)	
ENSPTRP00000052965 Araf(raf)	ENSP00000268763 Araf(raf)	
ENSPTRP00000046231 agc1(agc1)	ENSP00000346895 agc1(agc1)	
ENSPTRP00000022910 K-MLCK(camk1)	ENSP00000365152 K-MLCK(camk1)	
ENSPTRP00000000400 ptk11(ptk11)	ENSP00000351209 ptk11(ptk11)	
ENSPTRP00000000842 sp|Q95KR7|LCK_SAISC(PTK8)	ENSP00000328213539 sp|Q95KR7|LCK_SAISC(ptk8)	
ENSPTRP00000003178 SNF1(camk2)	ENSP00000356125 SNF1(camk2)	
ENSPTRP00000008008 BFR2_HUMAN/367-643(kinase)	ENSP00000075503 BFR2_HUMAN/367-643(kinase)	
ENSPTRP00000011128 Nek1(nima)	ENSP00000238616 Nek1(nima)	
ENSPTRP00000011806 STE20(pak)	ENSP00000260404 STE20(pak)	
ENSPTRP00000014478 Dcdrk(cmgc_other)	ENSP00000338673 Dcdrk(cmgc_other)	
ENSPTRP00000015133 7FpMPK(cmgc2)	ENSP00000299612 7FpMPK(cmgc2)	
ENSPTRP00000015183 Araf(raf)	ENSP00000323178 Araf(raf)	
ENSPTRP00000016161 NPK1(mekk_ste11)	ENSP00000354927 NPK1(mekk_ste11)	
ENSPTRP00000016162 NPK1(mekk_ste11)	ENSP00000354485 NPK1(mekk_ste11)	
ENSPTRP00000017707 MEK2(mek_ste7)	ENSP00000314228 MEK2(mek_ste7)	
ENSPTRP00000018244 Mast205(agc_other)	ENSP00000262811 Mast205(agc_other)	
ENSPTRP00000018925 Ark(ptk12)	ENSP00000301178 Ark(ptk12)	
ENSPTRP00000019125 p78(camk2)	ENSP00000300843 p78(camk2)	
ENSPTRP00000019170 DM(agc_other)	ENSP00000366979 DM(agc_other)	
ENSPTRP00000019171 DM(agc_other)	ENSP00000291270 DM(agc_other)	
ENSPTRP00000019172 DM(agc_other)	ENSP00000342201 DM(agc_other)	
ENSPTRP00000019651 agc2(agc2)	ENSP00000263431 agc2(agc2)	
ENSPTRP00000020517 VRK2_Hs(ck1)	ENSP00000342381 CKId(csnk)	
ENSPTRP00000022508 DdCKIIa(cmgc4)	ENSP00000371400 FpCKII(cmgc4)	
ENSPTRP00000026572 Nyk/RYK(ptk23)	ENSP00000296084 Nyk/RYK(ptk23)	
ENSPTRP00000027635 bPDGFR(ptk14)	ENSP00000257290 bPDGFR(ptk14)	
ENSPTRP00000029750 ckit(ptk14)	ENSP00000261799 ckit(ptk14)	
ENSPTRP00000029757 IIaCaMK(camk1)	ENSP00000305090 IIaCaMK(camk1)	
ENSPTRP00000032056 BYR2_SCHPO/394-658(kinase)	ENSP00000297332 BYR2_SCHPO/394-658(kinase)	
ENSPTRP00000032290 translationk(translationk)	ENSP00000199389 translationk(translationk)	
ENSPTRP00000032700 IIbCaMK(camk1)	ENSP00000326375 IIbCaMK(camk1)	
ENSPTRP00000032702 IIbCaMK(camk1)	ENSP00000326544 IIbCaMK(camk1)	
ENSPTRP00000036039 3PCTAIRE(cmgc1)	ENSP00000286878 3PCTAIRE(cmgc1)	
ENSPTRP00000036043 3PCTAIRE(cmgc1)	ENSP00000322343 3PCTAIRE(cmgc1)	
ENSPTRP00000037059 cAPKb(agc1)	ENSP00000310643 agc1(agc1)	
ENSPTRP00000037204 KKIALRE(cmgc_other)	ENSP00000369327 KKIALRE(cmgc_other)	
ENSPTRP00000037953 2RSKN(agc6)	ENSP00000262752 2RSKN(agc6)	
ENSPTRP00000041330 BYR2_SCHPO/394-658(kinase)	ENSP00000265125 BYR2_SCHPO/394-658(kinase)	
ENSPTRP00000045934 sp|Q95KR7|LCK_SAISC(PTK8)	ENSP00000362665 sp|Q95KR7|LCK_SAISC(ptk8)	
ENSPTRP00000047028 STE20(pak)	ENSP00000278568 STE20(pak)	
ENSPTRP00000047628 p78(camk2)	ENSP00000364449 p78(camk2)	
ENSPTRP00000053614 1ERK(cmgc2)	ENSP00000327293 1ERK(cmgc2)	
ENSPTRP00000053615 1ERK(cmgc2)	ENSP00000263025 1ERK(cmgc2)	
ENSPTRP00000053729 DdK2(agc_other)	ENSP00000282908 DdK2(agc_other)	
ENSPTRP00000054908 2RSKN(agc6)	ENSP00000368884 2RSKN(agc6)	
ENSPTRP00000055043 cAPKb(agc1)	ENSP00000262848 cAPKa(agc1)	
