# Supplementary material for: Comparative kinomics of human and chimpanzee reveal unique kinship and functional diversity generated by new domain combinations
Source: BMC Genomics. 2008 Dec 23;9:625. doi: 10.1186/1471-2164-9-625 (PMC2651890; doi:10.1186/1471-2164-9-625)
Supplement: Additional file 4 — The table representing different gene expression levels for human and chimp protein kinases in five different tissue types. [file 1471-2164-9-625-S4.rtf]

Additional file 4: The table representing different gene expression level for human and chimp protein kinase in five different tissue types. Abbreviations followed in the table: RMABRAIN, mean squared species difference in RMA-values for brain; RMAHEART, mean squared species difference in RMA-values for heart; RMAKIDNEY, mean squared species difference in RMA-values for kidney; RMALIVER, mean squared species difference in RMA-values for liver; RMATESTIS, mean squared species difference in RMA-values for testis. Data presented in this table has been obtained from reference [36].

Gene id	Protein kinase subfamily	RMABRAIN	RMAHEART	RMAKIDNEY	RMALIVER	RMATESTIS	
ENSG00000011566	(Unclassified)	0.0819	0.1232	0.2481	0.1994	0.5262	
ENSG00000012983	(Unclassified)	0.0764	0.104	0.327	0.2761	0.3018	
ENSG00000013441	(cmgc5)	0.1929	0.2861	0.394	0.729	0.5749	
ENSG00000027075	ePKC(agc2)	0.0673	0.0841	0.2576	0.1133	 	
ENSG00000028116	(ck1)	0.1126	0.2488	0.0507	0.0972	 	
ENSG00000038382	(camk1)	0.8694	0.1038	0.1419	0.095	0.1597	
ENSG00000050748	Jnk1(cmgc2)	0.0835	0.0895	0.0528	0.0986	0.0677	
ENSG00000055332	(translationk)	0.0381	0.0208	0.0483	0.0224	0.0715	
ENSG00000058091	1PCTAIRE(cmgc1)	1.2189	0.0498	0.0392	0.1437	0.0819	
ENSG00000059758	2PCTAIRE(cmgc1)	0.3815	0.0909	0.3138	0.0522	0.1144	
ENSG00000060140	(Unclassified)	0.013	0.1959	0.008	0.0172	0.0102	
ENSG00000065559	MEK2(mek_ste7)	0.5109	0.1213	0.2103	0.1054	0.2011	
ENSG00000065613	(Unclassified)	1.4657	0.1192	0.2528	0.1559	0.4042	
ENSG00000066056	Tek(ptk13)	0.0904	0.3348	 	 	 	
ENSG00000066468	FGFR-4(ptk15)	0.4197	0.1474	 	 	 	
ENSG00000068078	Bek(ptk15)	0.1067	0.7105	0.1071	0.1567	0.0916	
ENSG00000070770	a'CKII(cmgc4)	0.0755	0.044	0.15	0.1319	0.1663	
ENSG00000071054	(Unclassified)	1.1679	0.0955	0.4477	0.0573	0.1156	
ENSG00000071575	IIdCaMK(camk1)	7.482	0.3922	0.1204	0.2663	0.1087	
ENSG00000072062	agc1(agc1)	0.0243	0.0825	0.1582	0.0173	 	
ENSG00000072786	(Unclassified)	0.9443	0.1409	0.1748	0.142	0.409	
ENSG00000077782	Flg(ptk15)	0.0766	0.1088	0.075	0.0308	 	
ENSG00000083290	(Unclassified)	0.1016	0.0952	0.0119	0.0366	 	
ENSG00000086015	Mast205(agc_other)	0.0839	0.0543	0.0631	0.2542	0.3489	
ENSG00000092445	Brt/Sky(ptk12)	0.0502	0.0407	 	 	 	
ENSG00000096968	JAK2(ptk7)	0.0841	0.7191	0.1073	0.0404	 	
ENSG00000097007	ARG(ptk5)	0.5275	0.0456	0.3023	0.0853	0.0888	
ENSG00000101109	(Unclassified)	0.2364	0.0527	0.034	0.0259	0.1279	
ENSG00000102225	1PCTAIRE(cmgc1)	0.0544	0.074	0.0329	0.0491	 	
ENSG00000102572	(Unclassified)	1.3705	0.0474	0.1245	0.0449	0.1399	
ENSG00000102755	Flt1(ptk14)	0.1407	0.3497	0.4157	0.7971	0.1035	
ENSG00000104365	1KIN(camk2)	0.1041	0.175	0.2279	0.1089	 	
ENSG00000104936	DM(agc_other)	0.1423	0.0616	0.132	0.1166	0.0354	
ENSG00000105221	agc3(agc3)	0.0321	0.0481	0.1187	0.0326	 	
ENSG00000105810	6Cdk(cmgc1)	0.0683	0.0837	0.2098	0.0808	 	
ENSG00000106799	ALK-5(tgfb)	0.2748	1.2512	0.5214	0.3604	0.2048	
ENSG00000107140	(Unclassified)	0.0522	0.0427	 	 	 	
ENSG00000107643	Jnk1(cmgc2)	0.2186	0.075	0.0224	0.0904	0.4974	
ENSG00000107779	ALK-3(tgfb)	0.2037	0.3498	0.1364	0.1799	 	
ENSG00000109339	Jnk1(cmgc2)	0.0221	0.033	0.1161	0.0616	0.0112	
ENSG00000111816	(ptk8)	0.0909	0.1925	 	 	 	
ENSG00000113240	cmgc5(cmgc5)	0.241	0.3137	0.3159	0.4891	0.2163	
ENSG00000113263	Itk/Tsk(ptk2)	0.0086	0.0055	0.0428	0.022	0.0342	
ENSG00000114670	Nek1(nima)	0.1047	0.0506	0.0984	0.046	 	
ENSG00000114739	ActRIIB(tgfb)	0.3573	0.027	0.0457	0.2159	0.0606	
ENSG00000114904	Nek1(nima)	0.236	0.144	0.1934	0.2724	0.1177	
ENSG00000115170	TskL7(tgfb)	0.3326	0.0829	0.1253	0.2133	0.2146	
ENSG00000115694	(Unclassified)	0.073	0.1597	0.0573	0.0723	 	
ENSG00000115825	mPKC(agc2)	0.1999	0.3298	0.1934	0.1048	0.3887	
ENSG00000118046	SNF1(camk2)	0.0346	0.0229	 	 	 	
ENSG00000119408	NrkA(nima)	0.2953	0.342	0.1797	0.5481	0.2179	
ENSG00000120539	Sgk(agc_other)	0.702	0.0523	0.0395	0.0608	0.034	
ENSG00000126934	MEK2(mek_ste7)	0.1063	0.0346	0.0452	0.0631	0.2225	
ENSG00000130413	IIaCaMK(camk1)	0.2508	0.0658	0.1597	0.1223	0.0931	
ENSG00000132155	raf(raf)	0.2793	0.0988	0.1756	0.0317	 	
ENSG00000132356	(Unclassified)	0.2803	0.0195	0.1096	0.1271	0.4151	
ENSG00000133275	YCK2(csnk)	0.0298	0.0161	0.0423	0.0501	0.2154	
ENSG00000135333	Ehk-1(ptk11)	0.0557	0.2764	0.1632	0.0817	 	
ENSG00000135341	(Unclassified)	0.0837	0.0498	0.1776	0.3025	0.244	
ENSG00000136807	KIN28(cmgc1)	0.1191	0.1219	0.1577	0.266	0.0972	
ENSG00000137193	(Unclassified)	0.082	0.0667	0.1173	0.0793	0.38	
ENSG00000137275	DtSpk-1(ptk2)	0.0117	0.0845	0.0614	0.0127	 	
ENSG00000137843	STE20(pak)	0.1441	0.1243	0.0832	0.1841	 	
ENSG00000138756	(polo)	0.3607	0.0273	0.1806	0.0376	0.043	
ENSG00000139625	(Unclassified)	0.0159	0.0127	 	 	 	
ENSG00000140443	(ptk16)	0.1159	0.1456	0.2509	0.6626	0.0724	
ENSG00000140795	M-MLCK(camk1)	0.0132	0.0663	 	 	 	
ENSG00000140992	agc1(agc1)	0.067	0.3549	0.1221	0.2669	 	
ENSG00000141551	HRR25(csnk)	0.1278	0.3854	0.2327	0.035	 	
ENSG00000141736	ErbB2(ptk10)	0.0593	0.0803	 	 	 	
ENSG00000142149	1+kin(camk2)	0.0529	0.0093	 	 	 	
ENSG00000142875	cAPKb(agc1)	0.3515	0.0267	0.3024	0.2687	0.6691	
ENSG00000143674	PTK1(mlk)	0.2807	0.0132	0.0506	0.1137	0.085	
ENSG00000145242	Ehk-1(ptk11)	0.0057	0.1454	 	 	 	
ENSG00000145632	(polo)	0.1182	0.225	0.7634	0.0687	 	
ENSG00000146648	ptk10(ptk10)	0.275	0.1465	0.1601	0.0361	 	
ENSG00000147044	IIgCaMK(camk1)	0.0249	0.0202	0.0321	0.0981	0.1448	
ENSG00000147507	(ptk8)	0.3422	0.2682	0.1722	0.319	 	
ENSG00000148053	TrkB(ptk19)	0.6538	0.0311	0.7279	0.2385	 	
ENSG00000149930	(Unclassified)	0.0378	0.0088	 	 	 	
ENSG00000150457	(agc_other)	0.1971	0.3177	0.555	0.1618	 	
ENSG00000151292	YCK2(csnk)	0.0607	0.0553	0.1013	0.2013	0.3655	
ENSG00000153208	c-Eyk(ptk12)	1.1164	1.511	0.405	0.3262	0.4739	
ENSG00000162302	(agc6)	0.0061	0.027	0.0248	0.0194	0.0211	
ENSG00000162409	AKIN10(camk2)	0.0161	0.2681	0.1882	0.03	 	
ENSG00000162889	MAPKAP2(camk_other)	0.4417	0.0862	0.3578	0.0899	 	
ENSG00000163788	p78(camk2)	0.0972	0.0562	0.1737	0.2413	0.1067	
ENSG00000164715	Dror(ptk19)	0.3958	0.0969	0.1186	0.063	0.0808	
ENSG00000167657	IIdCaMK(camk1)	1.2975	0.0971	0.6945	0.0562	0.0941	
ENSG00000169032	mek(mek_ste7)	0.0991	0.1035	0.1343	0.0632	0.3076	
ENSG00000169118	CKId(csnk)	0.4939	0.0265	0.1298	0.0493	0.0604	
ENSG00000170145	p78(camk2)	0.0102	0.039	0.3724	0.0323	0.2791	
ENSG00000172071	PKR(translationk)	1.0238	0.1673	1.0743	0.4291	0.4833	
ENSG00000172939	(Unclassified)	0.3916	0.0717	0.1113	0.0858	0.1214	
ENSG00000173327	(mlk)	0.0748	0.0196	0.0148	0.0465	0.1562	
ENSG00000173846	(polo)	0.0754	0.0237	 	 	 	
ENSG00000174292	(ptk8)	0.0409	0.0148	 	 	 	
ENSG00000177169	(Unclassified)	0.2291	0.0457	0.0547	0.0566	0.29	
ENSG00000180370	STE20(pak)	0.2418	0.0767	0.389	0.0987	0.4525	
ENSG00000184216	(plantrk)	1.192	0.0318	0.3293	0.1873	0.4036	
