# Supplementary material for: Comparative kinomics of human and chimpanzee reveal unique kinship and functional diversity generated by new domain combinations
Source: BMC Genomics. 2008 Dec 23;9:625. doi: 10.1186/1471-2164-9-625 (PMC2651890; doi:10.1186/1471-2164-9-625)
Supplement: Additional file 5 — Population and distribution of chimpanzee protein kinases into various subfamilies. [file 1471-2164-9-625-S5.rtf]

Additional file 5: 
Population and distribution of chimpanzee protein kinases into various subfamilies
The chimpanzee genome encodes 587 PPKs and 98 protein kinase-like, non-kinases (PKLNKs) which are homologous to protein kinases but lack the crucial aspartate residue in the catalytic loop of the kinase catalytic domain. Hence these domains with good sequence similarity with kinase catalytic domains are unlikely function as a kinase. Of the 587 PPKs, 540 proteins are annotated in the published chimp genome dataset as “novel peptides” and not studied individually. Serine/Threonine kinases occur more than the Tyrosine protein kinases. 19% of the total PPKs (117 PPKs) could not be placed into any known families of Hanks and Hunter and hence are unclassified.

Many distinct protein kinase subfamilies belonging to the category of Serine/Threonine kinases have representatives from yeast to human, supporting the idea of early evolution of these kinase groups and their requirement for basic cellular functions. However members of protein tyrosine kinase group have been identified only in metazoan, reflecting their roles in complex signal transduction pathway. The distribution and diversity of various protein kinase subfamilies are mentioned in Table 1, and are discussed below.
Classification of chimpanzee PPKs
AGC group of protein kinases
  	AGC group is composed of PKA (cAMP dependent kinase), PKG (cGMP dependent kinase) and PKC (DAG dependent kinase), all of which are second messenger regulated protein kinases. We identified 81 PPKs in chimpanzee which are close homologues of AGC group of kinases. There are 11 putative c-AMP dependent protein kinases (PKA) identified in the chimpanzee. PKA are holoenzymes that consist of two catalytic subunits and two regulatory subunits. When the level of cAMP rises, the conformational change helps in the release of the catalytic subunit of PKA but when the level of cAMP is low, both the subunits remain intact and hence inactive. There are 6 putative PKA regulatory subunits (ENSPTRP00000045750, ENSPTRP00000004325, ENSPTRP00000004326, ENSPTRP00000047706, ENSPTRP00000025724 and ENSPTRP00000025721) which have been identified in the chimpanzee genome. cGMP dependent protein kinases (PKGs) are activated by cyclic Guanosine Mono Phosphate (cGMP). PKG phosphorylates a number of biologically important proteins which are implicated in the regulation of smooth muscle relaxation, nucleic acid synthesis etc. PKG is a single chain enzyme that includes the two copies of the c-NMP binding domain in the N-terminal. In the current analysis, three putative PKGs (ENSPTRP00000004325, ENSPTRP00000004326 and ENSPTRP00000027855) have been identified. The sub-family of Diacylglycerol-activated/phospholipid-dependent protein kinase C (PKC) has approximately 10 isoforms which can be categorized into three classes: conventional PKC, novel PKC and atypical PKC. The structure of all PKC consists of a regulatory domain and a catalytic domain. The requirement of second messenger differs for each isoform of PKC because of regulatory region, which is similar within class but different across classes. There are 16 PPKs in the chimpanzee genome which are close homologues of PKC. There are two PKC-like (ENSPTRP00000000076 and ENSPTRP00000026868) which have PB1 domain N-terminal to the kinase domain suggesting their role in ubiquitin pathway. Although the sequences of these catalytic domains are most closely resembling PKC these kinases have hybrid features of PKCs and those involved in degradation pathways.

There are two proteins (ENSPTRP00000019171 and ENSPTRP00000019170) which belong to AGC group, having transmembrane segments predicted C-terminal to the kinase domain which is very unusual. Like many other AGC group of kinases these kinases are likely to be localized near the membrane with C-terminal stretch embedded in the membrane.

Protein kinases involved in calcium signaling (CAMK group):
 	The calcium/calmodulin regulated kinases are present in fungi, plants and metazoa, suggesting this is quite an ancient regulatory signaling mechanism.  Many of the intracellular actions of Ca2+ in eukaryotic cells are mediated by the activation of CAMK family of protein kinases. The CAMK group consists of Ca2+/Calmodulin (CaM) activated protein kinases [CAMK1, CAMK2, DAPK, MLCK (Myosine Light Chain Kinase), CASK and PHK (Phosphorylase Kinase) families], and other related kinases that are not CaM regulated like CAMK-like (CAMKL).  The CAMKs share common structural and regulatory features. The kinase catalytic domain (which is relatively conserved) lies in the N-terminal region which is followed by C-terminal regulatory region. 

CAMK group is the most populated protein kinase subfamily identified in the chimpanzee genome. There are 91 putative protein kinases in chimpanzee which are closely related to CAMK subfamily. In the CAMK group, the most abundant subfamilies include CAMK1, CAMK2 and MLCK. CAMKs are maintained in an inactive state when cells do not need them by an intrasteric mechanism [1] and are activated by a high affinity interaction of the Ca2+/CaM complex with the regulatory domain which removes the autoinhibitory sequence from the active site. Calmodulin is the protein which regulates CAMKs by acting as a mediator of Ca2+ functions. There are 42 putative calmodulins identified in the chimpanzee genome. 
 
Of these 91 PPKs, 9 PPKs have calcium/calmodulin dependent protein kinase II Association (CaMKII_AD) domain which follows the kinase domain in the primary structure. The function of the CaMKII association domain is in the assembly of the single proteins into large (8 to 14 subunits) multimers [2]. Copies of DCX (Doublecortin) domain have been found tethered to the N-terminal of PK domain which is known to bind to the microtubule cytoskeleton, stabilizes microtubules and causes bundling.

CMGC group of protein kinases
 	CMGC group of protein kinase expands for the CDK (Cyclin Dependent protein Kinase), MAPK (Mitogen Activated Protein Kinase), GSK (Glycogen Synthase protein Kinase) and CK2 (Casein Kinase 2) subfamilies. We detected 75 PPKs which are close homologues of CMGC group in the chimpanzee genome.
Cyclin-dependent protein Kinase (CDKs): CDKs belong to a group of protein kinases involved in the regulation of cell cycle and also in the regulation of transcription and mRNA processing. CDKs are activated by physical association with cyclin. The CDK-cyclin complex is regulated by various other proteins kinases and phosphatases, including Wee and CDK activation kinase (CAK). Any deregulation of CDK in the cell cycle can lead to many diseases, mainly cancer hence CDKs are considered potential target for anti-cancer medication [3].
Chimpanzee has 20 putative CDKs which includes CDK2, CDK3, CDK5, CDK7 and CDK8. There are 39 close homologues of cyclins which might be involved in the regulation of chimpanzee CDKs, identified in the current analysis.
Mitogen Activated Protein Kinase (MAPK):   MAP kinases regulate cell cycle, cell proliferation, differentiation and apoptosis [4]. Disregulation in the MAPK signaling cascade is responsible for cancer [5]. Chimpanzee has 15 close homologues of MAP Kinases. There are various upstream effectors of MAPK, which are involved in the regulation of MAPK cascade. These effectors e.g MAPKK (MEK), MAPKKK (MEKK) are discussed elsewhere in this paper.
Glycogen Synthase protein Kinase 3 (GSK): GSK was first identified as a regulator of insulin synthesis via the regulation of glycogen but is also involved in cell differentiation, motility and apoptosis [6]. GSK is thus a target in cancer, diabetes, Alzheimer's disease and bipolar disorder [7, 8, 9, 10]. GSK is encoded by two related genes in mammalians: GSK3 alpha and GSK3 beta. Our chimpanzee kinome survey identified two putative GSKs: ENSPTRP00000026315 (close homologue of GSK3 beta) and ENSPTRP00000018979 (close homologue of GSK3 alpha).
Casein Kinase2 (CK2): CK2 is a ubiquitous enzyme present in the cytoplasm and nucleus, and has a very large number of known protein substrates. It is found usually as a tetrameric complex which consists of two catalytic subunits (alpha and/or alpha') and two regulatory subunits which stimulate the activity of the enzyme and affects its specific interactions with substrates and inhibitors. CK2 participates in a complex series of cellular processes like cell growth, differentiation and suppression of apoptosis in cells [11]. The anti-apoptotic property of CK2 can be exploited very well in the cancer therapeutics [12]. We identified two PPKs in chimpanzee (ENSPTRP00000022508 and ENSPTRP00000053525) which are close homologues of casein kinase 2.
STE group
   	The involvement of various protein kinases like MAPKK, MAPKKK regulate the MAPK cascade which play pivotal role in transmission of extracellular signal to the intracellular targets which further initiates various cellular processes such as cell growth, differentiation and development [13]. In the present analysis, we have identified 8 putative MEK/ste7 and 10 putative MEKK/ste11. In one of the MEKs (ENSPTRP00000012334) and three MEKK (ENSPTRP00000016161, ENSPTRP00000016162 and ENSPTRP00000021280) PB1 (Phox and Bem1p) domain is present N -terminal to the kinase domain. It is related to ubiquitin superfamily, and it might have role in ubiquitin pathway. One of the MEKKs (ENSPTRP00000048240) has SWIM domain N-terminal to the PK domain which is a Zn-chelating domain and found in a variety of prokaryotic and eukaryotic proteins [14].
Casein Kinase 1: CK1 protein kinases are conserved from yeast to higher eukaryotes. They are involved in diverse and important cellular functions like cell division, cell repair, circadian rhythms and nuclear localization [15]. In the cytosol, CK1 is known to act in conjunction with c-AMP dependent protein kinase (PKA) to phosphorylate and downregulate the activity of glycogen synthase [16]. There are 17 close homologues of CK1 identified in the chimpanzee. A putative CK1 (ENSPTRP00000020517) has a C-terminal transmembrane helix, predicted with high confidence, which is very unusual since CK1 is known to be a cytoplasmic protein. A putative CK1 (ENSPTRP00000001150) is closely related to polo kinase in terms of the domain combination because it has POLO-BOX domain tethered N-terminal to the protein kinase domain. The dendrogram (Figure 1) generated for the CK1 subfamily members of chimpanzee and human clearly shows how diverse this putative protein kinase is. These close homologues of CK1 are likely to be sharing properties of other kinases and hence should be considered as hybrid kinases rather than CK1. 
Protein Tyrosine Kinase (PTK) group
	PTK group encompasses 23 subfamilies which can be broadly grouped into receptor PTK and non-receptor PTK. Receptor PTK are transmembrane proteins composed of an extracellular ligand-binding domain, transmembrane domain and an intracellular PK domain. Some of the receptor PTKs such as insulin receptor, are known to form dimers. The binding of ligand on the extracellular domain promotes the dimerization of the receptor, which in turn results in the activation by trans-autophosphorylation of the PK domains [17].

 The activation of PTK triggers many intra-cellular reactions, involving the MAPK cascade, that ultimately control cell proliferation and survival. It has been shown that some PTK, e. g.,  insulin-like growth factor-I receptor, vascular endothelial growth factor receptor and epidermal growth factor receptor  are either over-expressed or constitutively activated in lung tumors and acute myeloid leukemia cells. These PTKs thus constitute popular drug targets for cancer treatment [18, 19]. Non-receptor PTKs do not possess any transmembrane segment but can bind to membrane receptors. PTKs are involved in cell growth and differentiation, and are targets of cancer treatments [20]. There are 64 close homologues of receptor PTK and 52 close homologues of non-receptor PTK identified in the chimpanzee. The expansion of receptor PTK in human occurs in the ptk15 subfamily (platelet -derived growth factor receptor family), with 8 PPKs in chimpanzee and 12 in human.


Other protein kinases encoded in the chimpanzee genome
 	Raf protein kinase is a part and upstream activator of MAPK cascade. It is a conserved component of eukaryotic genomes. Many mutations in raf genes lead to cancer [21]. There are 14 close homologues of raf protein kinases identified in the chimpanzee genome. A raf homologue (ENSPTRP00000034905) has caspase-recruitment domain (CARD) C-terminal to the kinase domain and hence this protein might have role in apoptotic signaling. Two raf homologues (ENSPTRP00000033866 and ENSPTRP00000025252) have typical raf kinase like architecture in which RBD (Ras Binding Domain) domain is followed by C1_1 domain (Phorbol esters/diacylglycerol binding domain) which is followed by a catalytic kinase domain. 

Six homologues of p-21 activated kinase (PAK) have also been identified. PAK signaling is implicated in angiogenesis, cell proliferation, survival and/or death pathway; all hallmarks of oncogenic transformation. Six close homologues of translation kinases (involved in the regulation of translation) have been identified in the current analysis. 14 genes are encoding for NimA protein kinase in the chimpanzee genome. The NimA kinases are involved in cell cycle regulation [22]. We identified 16 close homologues of activin/tgf beta receptor kinases, which are known to be implied in various diseases such as angiogenesis and renal disease [23].

Interestingly 6 PPKs have similarity with flowering plant putative receptor kinase family (plantrk). Surprisingly, none of these six PPKs posses transmembrane region. We identified 4 close homologues of polo kinases. Polo kinases are involved in a variety of mitotic processes including spindle assembly, kinetochore function and in cytokinesis. The general architecture of polo kinase is, kinase domain followed by polo-box domain. Polo-box targets the kinase to specific substrates and subcellular locations. But polo-box domain could not be detected in two putative polo kinases (ENSPTRP00000053553 and ENSPTRP00000027232), which leaves an open question of their function. Two close homologues of wee1 kinase have been identified which might have role in cell cycle regulation. Wee1 kinase adds phosphate group to CDK which inhibits the CDK action. 

Unclassified protein kinases
Apart from the protein kinase subfamilies which have been discussed above, there are protein kinases which could not be placed into any of the known Hanks and Hunter subfamily. These 117 kinases are thus labeled as unclassified kinases. A majority of unclassified kinases display architecture without any accessory domain: 82 out of 117 PPKs are only composed of only one protein kinase domain. Among the remaining 35 unclassified PPKs, 16 PPKs have the citron homology domain found C-terminal to the protein kinase domain. The citron homology domain is a regulatory domain known to be involved in macromolecular interaction. In our kinome survey, the citron homology domain was found tethered to AGC kinases also.

Four unclassified chimpanzee PPKs (ENSPTRP00000042257, ENSPTRP00000021569, ENSPTRP00000021572 and ENSPTRP00000004014) encompass a Myosin head domain, accompanied by the IQ calmodulin-binding motifs in three cases (ENSPTRP00000042257, ENSPTRP00000021569 and ENSPTRP00000004014). These domains suggest role of kinase in motor activity. Two chimpanzee PPKs (ENSPTRP00000011804 and ENSPTRP00000021143) have been identified with a Mad3/BUB1 homology domain. This domain is usually seen in proteins controlling cell division [24], suggesting a potential role of these two PPKs in cell cycle. We have identified a PPK (ENSPTRP00000026520) containing HEAT and WD40 repeats. Both these repeats occur in a number of proteins, and are involved in protein-protein interaction and in human disease like Huntington's disease [25, 26].

A chimpanzee PPK (ENSPTRP00000028220) and its human counterpart (ENSP00000270861) posses a Sak_polo domain following the catalytic protein kinase domain in the sequence. This domain is known to be involved in mitotic sub-cellular localization [27], suggesting a role of PPK in cell division control. Further, the multiple sequence alignment of the kinase domains of the unclassified protein kinases have been used to generate sequenced similarity-based dendrogram (Figure 2). 

References cited in this additional information file:

1.  Soderling TR: Structure and regulation of calcium/calmodulin-dependent protein kinases II and IV. Biochim Biophys Acta 1996, 1297:131-138.

2. Gangopadhyay SS, Barber AL, Gallant C, Grabarek Z, Smith JL, Morgan KG: Differential functional properties of calmodulin-dependent protein kinase I gamma  variants isolated from smooth muscle. Biochem J 2003, 372:347-357. 

3. Garrett MD, Fattaey A: CDK inhibition and cancer therapy. Curr Opin Genet Dev 1999, 9:104-11. 

4. Avruch J: MAP kinase pathways: the first twenty years. Biochim Biophys Acta 2007, 1773:1150-1160.

5. Dhillon AS, Hagan S, Rath O, Kolch W: MAP kinase signalling pathways in cancer. Oncogene 2007, 26:3279-3290. 

6. Forde JE, Dale TC:  Glycogen synthase kinase 3: a key regulator of cellular fate.
Cell Mol Life Sci 2007, 64:1930-1944. 
7. Gould TD, Zarate CA, Manji HK: Glycogen synthase kinase-3: a target for novel bipolar disorder treatments. J Clin Psychiatry 2004, 65:10-21.  

8. Henriksen EJ, Dokken BB: Role of glycogen synthase kinase-3 in insulin resistance and type 2 diabetes. Curr Drug Targets 2006, 7:1435-1441.

9. Huang HC, Klein PS: Multiple roles for glycogen synthase kinase-3 as a drug target in Alzheimer's disease. Curr Drug Targets 2006, 7:1389-1397. 
10. Garcea G, Manson MM, Neal CP, Pattenden CJ, Sutton CD, Dennison AR, Berry DP: Glycogen synthase kinase-3 beta; a new target in pancreatic cancer? Curr Cancer Drug Targets 2007, 7:209-215. 

11. Litchfield DW: Protein kinase CK2: structure, regulation and role in cellular decisions of life and death. Biochem J 2003, 369:1-15.

12. Unger GM, Davis AT, Slaton JW, Ahmed K: Protein kinase CK2 as regulator of cell survival: implications for cancer therapy. Curr Cancer Drug Targets 2004, 4:77-84. 

13. Seger R, Krebs EG:  The MAPK signaling cascade. FASEB J 1995, 9:726-735.

14. Makarova KS, Aravind L, Koonin EV: SWIM, a novel Zn-chelating domain present in bacteria, archaea and eukaryotes. Trends Biochem Sci 2002, 27:384-386.

15. Gross SD, Anderson RA: Casein kinase I: spatial organization and positioning of a multifunctional protein kinase family. Cell Signal 1998, 10:699-711.

16. Flotow H, Roach PJ: Synergistic phosphorylation of rabbit muscle glycogen synthase by cyclic AMP-dependent protein kinase and casein kinase I. Implications for hormonal regulation of glycogen synthase. J Biol Chem 1989, 264:9126-9128.

17. Schlessinger J: Cell signaling by receptor tyrosine kinases.Cell2000, 103:-225.

18. Fischer B, Marinov M, Arcaro A: Targeting receptor tyrosine kinase signalling in small cell lung cancer (SCLC): what have we learned so far?Cancer Treat Rev2007, 33:391-406.

19. Doepfner KT, Boller D, Arcaro A: Targeting receptor tyrosine kinase signaling in acute myeloid leukemia. Crit Rev Oncol Hematol 2007, 63:215-230. 

20. Smith JK, Mamoon NM, Duhe RJ: Emerging roles of targeted small molecule protein-tyrosine kinase inhibitors in cancer therapy. Oncol Res 2004, 14:175-225.

21. Wellbrock C, Karasarides M, Marais R: The RAF proteins take centre stage. Nat Rev Mol Cell Biol 2004, 5:875-885. 

22. Fry AM, Nigg EA: Cell cycle. The NIMA kinase joins forces with Cdc2. Curr Biol 1995, 5:1122-1125. 

23. Graham H, Peng C:  Activin receptor-like kinases: structure, function and clinical implications. Endocr Metab Immune Disord Drug Targets 2006, 6:45-58. 

24. Hardwick KG, Johnston RC, Smith DL, Murray AW: MAD3 encodes a novel component of the spindle checkpoint which interacts with Bub3p, Cdc20p, and Mad2p.J Cell Biol2000, 148:-882.  

25. Andrade MA, Bork P: HEAT repeats in the Huntington's disease protein.Nat Genet1995, 11:-6. 

26. Li D, Roberts R: WD-repeat proteins: structure characteristics, biological function, and their involvement in human diseases. Cell Mol Life Sci2001, 58:-2097.

27. Leung GC, Hudson JW, Kozarova A, Davidson A, Dennis JW, Sicheri F: The Sak polo-box comprises a structural domain sufficient for mitotic subcellular localization. Nat Struct Biol2002, 9:-724.
